# Inverse Molecular Design for the Discovery of Organic Energy Transfer Photocatalysts: Bridging Global and Local Chemical Space Exploration

**DOI:** 10.1021/jacs.5c20087

**Published:** 2026-02-06

**Authors:** Leon Schlosser, Nils H. Rendel, Julius Gemen, Frank Glorius, Kjell Jorner

**Affiliations:** † Organisch-Chemisches Institut, 9185 University of Münster, Corrensstraße 36, 48149 Münster, Germany; ‡ Institute of Chemical and Bioengineering, Department of Chemistry and Applied Biosciences, 27219 ETH Zurich, Vladimir-Prelog-Weg 1, 8093 Zürich, Switzerland; § NCCR Catalysis, ETH Zurich, Switzerland

## Abstract

The discovery of new organic photocatalysts (PCs) for energy transfer (EnT) catalysis remains a significant challenge, largely due to the vast and underexplored chemical space and the delicate balance of the photocatalytic properties. While transition-metal catalysts are effective, their high cost and environmental impact necessitate the development of metal-free alternatives. In this work, we present a hybrid inverse molecular design strategy that combines global exploration with targeted local optimization to discover highly efficient organic PCs. Our approach leverages a generative model, guided by machine learning predictions and semiempirical simulations, to efficiently navigate chemical space and identify promising molecular scaffolds. We demonstrate the utility of this strategy by rediscovering known PCs and, more importantly, exploring uncharted structural regions, leading to the identification of novel candidates with favorable photophysical properties. A subsequent local exploration stage, using quantum mechanical calculations, allows refinement of the properties as well as control of the synthetic complexity. The practical applicability of the approach is demonstrated by performing a local exploration of one of the identified scaffolds and successfully synthesizing four candidate PCs. We showcase their catalytic aptitude in three different EnT-mediated reactions, including a challenging aza-photocycloaddition, where one of our designed PCs achieved 90% yield, a performance comparable to a state-of-the-art iridium-based catalyst. This study highlights the power of a data-driven inverse design framework to bridge computational discovery and experimental validation, accelerating the identification of novel PCs and expanding the scope of EnT catalysis.

## Graphical abstract

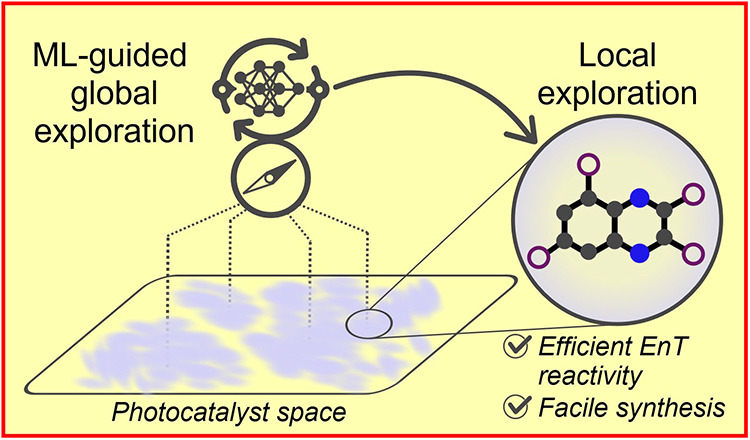

## Introduction

Energy transfer (EnT) photocatalysis has emerged as a powerful tool in synthetic organic chemistry, unlocking the rich reactivity of triplet excited states under mild conditions. This unique reactivity enables a wealth of transformations for the construction of valuable scaffolds, which are challenging to achieve by other means, e.g., ground-state reactivity.^1,2^ The choice of employed photocatalyst (PC) is paramount to promote efficient and selective reactivity. There are two distinct classes of PCs: transition-metal-based and organic PCs. The former, particularly those containing ruthenium or iridium, are very established due to their apt photophysical properties, such as high absorption in the visible light spectrum as well as efficient intersystem crossing (ISC) to populate long-lived triplet excited states. However, the high cost and environmental impact associated with the metals’ mining and processing pose a major challenge, particularly for an industrial application.^3^ Moreover, issues related to catalyst separation and potential toxicities further increase the barrier of employing metal-based PCs, particularly in pharmaceutical production.^4^ Meanwhile, organic PCs overcome these limitations and are attractive choices for enabling more environmentally benign EnT-mediated reactions.

Despite their promise, the design of efficient organic PCs remains a significant challenge. The success of EnT catalysis relies on a delicate balance of photophysical properties, such as high triplet energy, efficient ISC to populate long-lived triplet excited states, and high absorption in the visible light spectrum, which must be simultaneously optimized to an optimal trade-off. Traditionally, the design of new PCs has relied on trial-and-error as well as rational design-based approaches.^5^ However, the exploration of chemical space has remained confined due to human bias and the slow, iterative nature of traditional discovery approaches.^6^ As a result, only few structural classes have emerged as particularly successful and are now widely used, e.g., aryl ketones and cyanoarenes.^7,8^ However, these represent only a small fraction of the vast chemical space potentially suitable for enabling EnT reactivity (Figure 1A). Many regions remain unexplored, and existing organic PCs exhibit limitations such as limited photostability,^9,10^ side reactivity due to hydrogen atom abstraction of aryl ketones,^11,12^ or triplet depopulation through reverse ISC in cyanoarenes.^13^ Therefore, identifying novel molecular scaffolds that can act as efficient EnT catalysts is highly desirable to overcome these limitations and push the boundaries of EnT catalysis.

**Figure 1:**
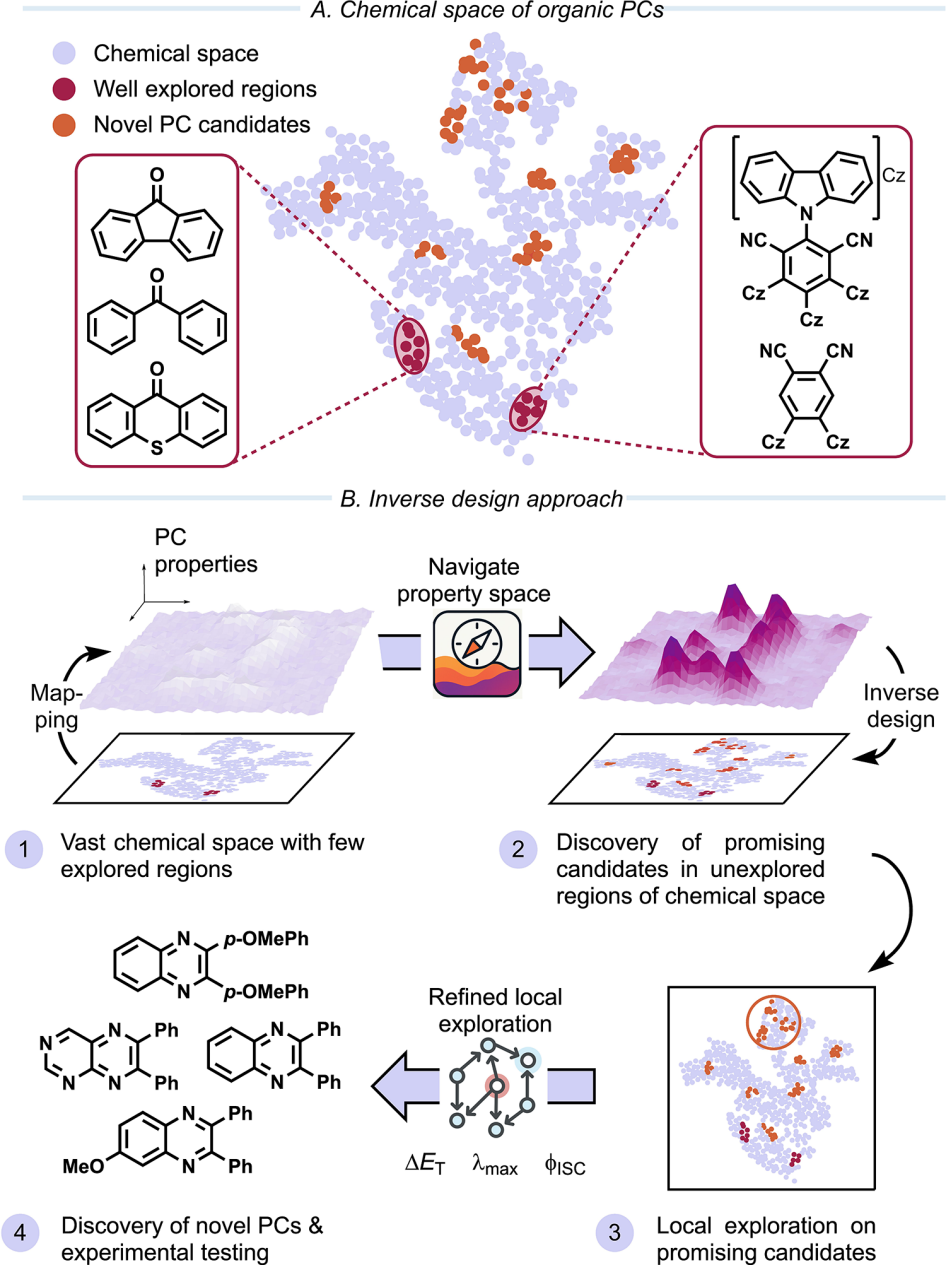
(A) Schematic depiction of the chemical space of the organic PCs. Explored regions of organic PCs are scarce and centered around a few well-explored scaffolds, such as aryl ketones or cyanoarenes. Many regions remain unexplored, and efficient exploration strategies are required to identify novel PC candidates. (B) This can be achieved through an inverse design approach, where the chemical space is mapped to the space of photocatalytic properties (1). Navigating this space enables the discovery of promising candidates in unexplored regions of the chemical space (2). A focused local exploration can be performed on promising candidates from the global exploration (3), enabling the discovery of novel PC scaffolds for EnT catalysis (4), which are subsequently synthesized and experimentally tested.

Recently, data-driven inverse design approaches have emerged as powerful tools to accelerate the exploration by navigating the property space rather than the structural space.^14^ Among these inverse design strategies, generative modeling has gained particular attention for its ability to produce novel molecular structures and explore underrepresented regions of chemical space.^15,16^ By capturing the underlying structure–property distributions, generative models can efficiently propose new molecules optimized for multiple, and often competing, properties.^17^ Despite the tremendous potential of generative modeling to discover novel organic PCs, its application in photocatalysis remains unexplored. Previous machine learning (ML) efforts in this field have instead focused on predictive or screening-based approaches where manually constructed virtual libraries were systematically explored using data-driven strategies.^18,19^ While these reports are landmark contributions and demonstrate the power of data-driven approaches in photocatalysis, the chemical space was inherently constrained by the need to define an initial virtual library.

In this work, we instead propose an inverse design approach aimed at identifying molecules with an optimal set of properties for efficient EnT-mediated reactivity. To achieve this goal, the strategy is twofold: combining global exploration with targeted local optimization. First, a generative model is utilized to enable an exhaustive chemical space exploration and facilitate the discovery of novel, previously unconsidered molecular scaffolds with optimal properties for EnT catalysis. This phase relies on computationally efficient methods to rapidly evaluate large numbers of candidates, enabling streamlined navigation in the property space and facilitating the discovery of new organic PC scaffolds.

Based on promising structures identified in the global exploration stage, a subsequent focused local search allows the refinement of the photocatalytic properties using quantum mechanical (QM) calculations (Figure 1B). Crucially, this phase also enables us to control the synthesizability, which is a major obstacle in applying generative models to practical use cases and experimentally evaluating their outcomes.^15,20^ The presented inverse design approach enables efficient chemical space navigation, thereby facilitating the discovery of new organic PC scaffolds with promising photocatalytic properties. The effectiveness and direct applicability of this approach are finally demonstrated by the synthesis and successful application of four identified PCs in three EnT-mediated reactions.

## Results and Discussion

### Generative Model

In EnT catalysis, the efficiency of a PC is governed by a delicate interplay of properties, such as the adiabatic triplet energy (Δ*E*_T_), the maximum absorption wavelength (λ_max_), the efficiency of the ISC (*Φ*_ISC_) to populate the triplet state, or the lifetime of the triplet state. In principle, any molecule having a favorable combination of these properties could serve as an effective EnT PC. However, identifying such molecules within the vast and sparsely mapped chemical space remains a challenge. To enable the generation of diverse structures with suitable properties for EnT catalysis, we utilized a data-driven generative workflow that integrates the prediction of properties with molecular design. Through a user-defined reward function, a recurrent neural network (called “agent” from now on) progressively learns to generate molecules that maximize the reward, thereby guiding the exploration of chemical space to regions with desired photocatalytic properties. By applying a scaffold similarity filter, the broad exploration of diverse structures is promoted (see Supporting Information). To enable simultaneous optimization of multiple objectives, the predicted property values are transformed between 0 and 1 using sigmoid and step functions. Those transformed values are weighted and aggregated to yield a final score (Figure 2a). For the generative modeling, the open-source platform REINVENT 4 served as the framework to enable flexible molecular design.^21^

**Figure 2:**
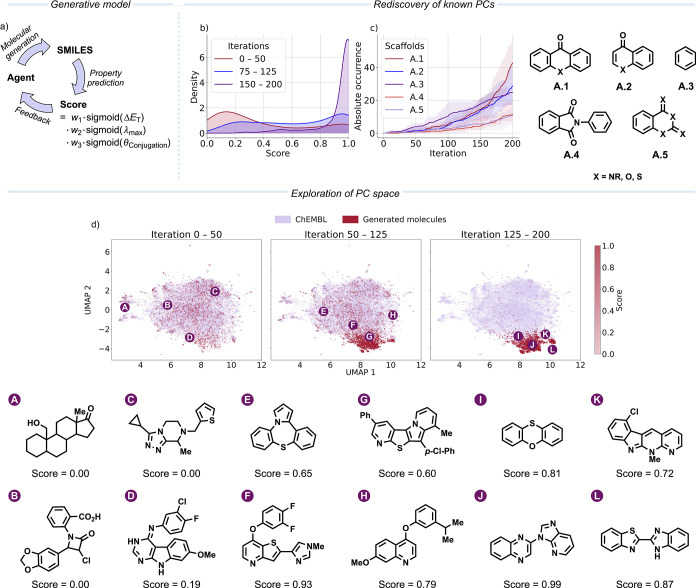
(a) Schematic depiction of the generative model. An agent is trained to generate molecules as SMILES. Those molecules are evaluated, and a score is computed by the weighted (*w*_i_) multiplication of the different components. This score is fed back to the agent, which updates its molecular generation policy to generate SMILES that maximize the score. (b) Density plot of the scores of the top 10 molecules from each generation over the course of the iterative generation. (c) Cumulative occurrence of generalized scaffolds averaged over five independent runs. The mean occurrence is plotted as the solid line, and the area indicates the minimum and maximum values observed. (d) UMAP projection of a subset of ChEMBL (50,000 molecules) and the generated molecules (10,834 molecules) at different steps during the generative process. Exemplary structures with corresponding scores from the chemical space are depicted.

For an exhaustive chemical space exploration, the photophysical properties need to be efficiently predicted to guide the model toward the generation of candidates with suitable properties. To achieve this, ML models trained to predict the desired quantities are particularly interesting, as they can provide rapid predictions with high accuracy, given that the model can generalize sufficiently well to molecules not seen during training. For the prediction of triplet energies, the EnTdecker model was employed.^22^ This graph-based model was trained on a diverse set of 34,850 molecules and demonstrated a predictive performance suitable for the desired task (MAE: 2.17 kcal/mol, *R* ^2^: 0.925 on a scaffold split, see Supporting Information for additional benchmarks). For the prediction of *λ*_max_, a multifidelity model proposed by Gómez-Bombarelli et al. was used.^23^ In comparison to Δ*E*_T_, *λ*_max_ is more solvent-dependent, which makes the data-curation as well as the prediction more challenging, resulting in a lower but still adequate accuracy (MAE: 27.78 nm, *R* ^2^: 0.80 on a scaffold split). Moreover, computationally efficient semiempirical calculations based on the simplified Tamm–Dancoff approach (sTDA) were used to predict *λ*_max_ (denoted as *λ*_max‑sTDA_).^24^ Benchmarking this method on 1326 experimental absorption spectra revealed a moderate accuracy (MAE: 39.22 nm, *R* ^2^: 0.404, see Supporting Information). Despite the inferior performance of using sTDA, it provides a complementary approach to the ML prediction due to its physical grounding, making it particularly valuable for scaffolds outside the ML training domain. Owing to its favorable computational efficiency, however, *λ*_max_ is obtained using ML predictions unless indicated otherwise. In addition to Δ*E*_T_ and*λ*_max_, the fraction of conjugated bonds in the molecule (θ_conjugation_) served as an additional property in the reward function to favor rigid scaffolds that impede intramolecular relaxation of the triplet state, which reduces the molecules’ triplet state lifetime.^25^

### Exploration of Photocatalyst Space

With those models at hand, the ability of the generative model to explore the chemical PC space was probed. First, it was investigated whether known organic PCs could be rediscovered by the model, considering only Δ*E*_T_, λ_max_, and θ_conjugation_ in the reward function, as *Φ*_ISC_ and the triplet lifetime are difficult to predict. This would serve as a confirmation that the property prediction models are suitable for the task of discovering organic PCs for EnT catalysis. Indeed, by optimizing for molecules with Δ*E*_T_ > 58 kcal/mol, λ_max_ > 365 nm, and θ_conjugation_ > 0.5 using (double) sigmoid functions, the model progressively learns to generate molecules that maximize the score (Figure 2b), among which structures containing scaffolds of established PCs, most notably aryl ketones such as thioxanthone and benzophenone,^7,9^ occur across all five independent runs. Analysis of the scaffold frequency throughout the iterative generation process further revealed that the model increasingly generates structures containing aryl ketones (**A.1**, **A.2**, Figure 2c), indicating that these structures tend to maximize the multiobjective reward function and are thus preferentially generated by the agent. In contrast, structures containing a benzene core (**A.3**) showed only a linear increase in the cumulative occurrence. While this scaffold is very abundant, it does not systematically maximize the reward, wherefore its occurrence progresses at a lower rate. It is important to point out that both **A.2** and **A.3** are also substructures of **A.1**. However, since the scaffolds are obtained as generalized Murcko scaffolds,^26^ only one (the largest) scaffold is assigned per generated molecule, avoiding redundant counting. Moreover, for phthalimide (**A.4**) and bicyclic heteroarenes (**A.5**), a moderate exponential increase in the total occurrence can tentatively be observed, suggesting favorable properties for these scaffolds.

Having confirmed that the generative workflow is successful in rediscovering established organic PCs, we next directed it toward the exploration of PC space and the discovery of novel molecular scaffolds to promote EnT reactivity. To guide the exploration beyond known motifs, structures containing carbonyl groups, a prevalent substructure in the rediscovery campaign, were excluded to steer the model toward uncharted regions in chemical PC space.

This also serves the purpose of mitigating the risk of undesired hydrogen atom transfer (HAT) reactions that are common among aryl ketones.^11,12^ Moreover, additional structural motifs that are prone to potential side reactivity or photodecomposition, such as bonds between two heteroatoms,^27^ aliphatic amines,^28^ alcohols,^29^ or olefins,^30^ were excluded by assigning molecules that contain these substructures a score of zero. Guided by these design principles, the generative run was performed to explore uncharted regions of PC space to generate molecules with Δ*E*_T_ > 55 kcal/mol, λ_max_ > 330 nm, and θ_conjugation_ > 0.5, yielding 10,834 unique structures. Overlaying these molecules onto a representative subset of the ChEMBL 25 database (50,000 molecules) reveals that, during the first iterations, the chemical space gets broadly explored (Figure 2d). However, most regions exhibit low scores owing to the applied design criteria. During the iterative generative process, the agent progressively learns to create molecules with higher scores and avoid the excluded structural motives. As a result, chemical space exploration becomes increasingly focused on regions consistent with the targeted structural requirements, while scaffold-based diversity filtering plays a complementary role. This leads to the generation of a wide variety of aryl-substituted fused heterocycles (Figure 2d), demonstrating the effectiveness of the generative model to navigate PC space and providing a powerful route toward identifying new classes of PCs with tailored EnT properties.

### Estimation of ISC Rate

While the generated molecules resemble promising candidates as organic PCs based on their predicted Δ*E*_T_ and λ_max_, another crucial prerequisite is an efficient population of the triplet state to promote the desired reactivity. We therefore decided to include the ISC quantum yield (*Φ*_ISC_) in the reward function. This property quantifies the efficiency with which absorbed photons lead to triplet state formation. In contrast to Δ*E*_T_ and λ_max_, however, generalizable ML prediction models are not available for *Φ*_ISC_, making it computationally costly to include this property in the molecular design process. To mitigate the computational cost, we exploited an approximation of *Φ*_ISC_ using Fermi’s golden rule, which establishes a relationship between the rate of ISC (from S_1_ to T_1_), the spin–orbit coupling (SOC) between singlet and triplet states, and their energy difference.^31^

$${k_{\mathrm{ISC}}\propto \frac{\left\langle S_{1}\left|H_{\mathrm{SO}}\right|T_{1}\right\rangle }{{\left(\Delta E_{S_{1}-T_{1}}\right)}^{2}}}^{2}$$

When describing both S_1_ and T_1_ states as single-electron HOMO→LUMO excitations, Δ*E*_S_1_–T_1__ is largely governed by the electron exchange interaction, which decreases with reduced spatial overlap between the orbitals.^32^ As calculation of the SOC is computationally demanding, we decided to focus on the HOMO–LUMO overlap (*O*_FMO_). Minimizing this overlap not only reduces Δ*E*_S_1_–T_1__ but also suppresses radiative decay from S_1_ to S_0_, favoring *Φ*_ISC_.^33^ Furthermore, SOC is enhanced when the transition involves a change in electronic character, such as from a charge-transfer singlet to a locally excited triplet.^34^ Capturing these relationships to maximize *k*_ISC_ within the molecular design process requires computationally efficient methods to enable large-scale generative modeling. For this, semiempirical methods offer a practical compromise between computational cost and a sufficiently accurate description of electronic character. Pleasingly, a moderately strong correlation (*R* ^2^: 0.631) could be achieved between reported Δ*E*_S_1_–T_1__ and *O*_FMO_ computed with GFN2-xTB.^35^ Moreover, by using *O*_FMO_, the reported electronic character, e.g., charge-transfer vs local excitation, of S_1_ and T_1_ could be correctly classified in 79% of the cases (see Supporting Information). Despite the remarkable predictive accuracy of this computationally efficient approach, it should be noted that estimating the nature of excited states using frontier molecular orbitals (FMO) can give only a qualitative picture. Moreover, ISC from S_1_ to higher lying triplets may contribute to *Φ*_ISC_, which cannot be described in this approach.^36^ Nonetheless, for the desired task of broadly exploring chemical space, this approach serves as a computationally tractable proxy to guide the generative model toward molecules with an efficient population of the triplet state to mediate EnT reactivity.

When performing five independent generative runs to optimize for molecules with Δ*E*_T_ > 55 kcal/mol, λ_max_ > 330 nm, *O*_FMO_ > 0.5, and θ_conjugation_ > 0.5, a diverse set of molecular scaffolds is obtained. Among the most occurring scaffolds are aryl-substituted mono- and bicyclic N-heterocycles such as aryl-substituted quinolines and pyridines (Figure 3a). Notably, in comparison to the previous chemical space exploration without *O*_FMO_, the inclusion of *O*_FMO_ in the generative process increased the frequency of nonfused ring structures that were generated by the model (Figure 3b,c). On average, a higher score for *O*_FMO_ (e.g., a lower HOMO–LUMO overlap) is obtained for molecules containing nonfused rings, which favors their generation (Figure 3d,e). Through the twisted structure between the monocyclic core and aryl substituents, *O*_FMO_ is minimized by spatially separating the donor and acceptor moieties in these molecules (see Supporting Information). This is also a common design principle in thermally activated delayed fluorescence (TADF) molecules.^37^ While molecules with nonfused rings achieve a higher score for *O*_FMO_, the reduced *p*-orbital overlap of the π-system in aryl-substituted monocyclic arenes simultaneously leads to a lower mean λ_max_, which is undesired. On the other hand, higher Δ*E*_T_ scores are obtained for these molecules, underscoring the challenge of delicately balancing the desired properties in multiobjective optimization.

**Figure 3:**
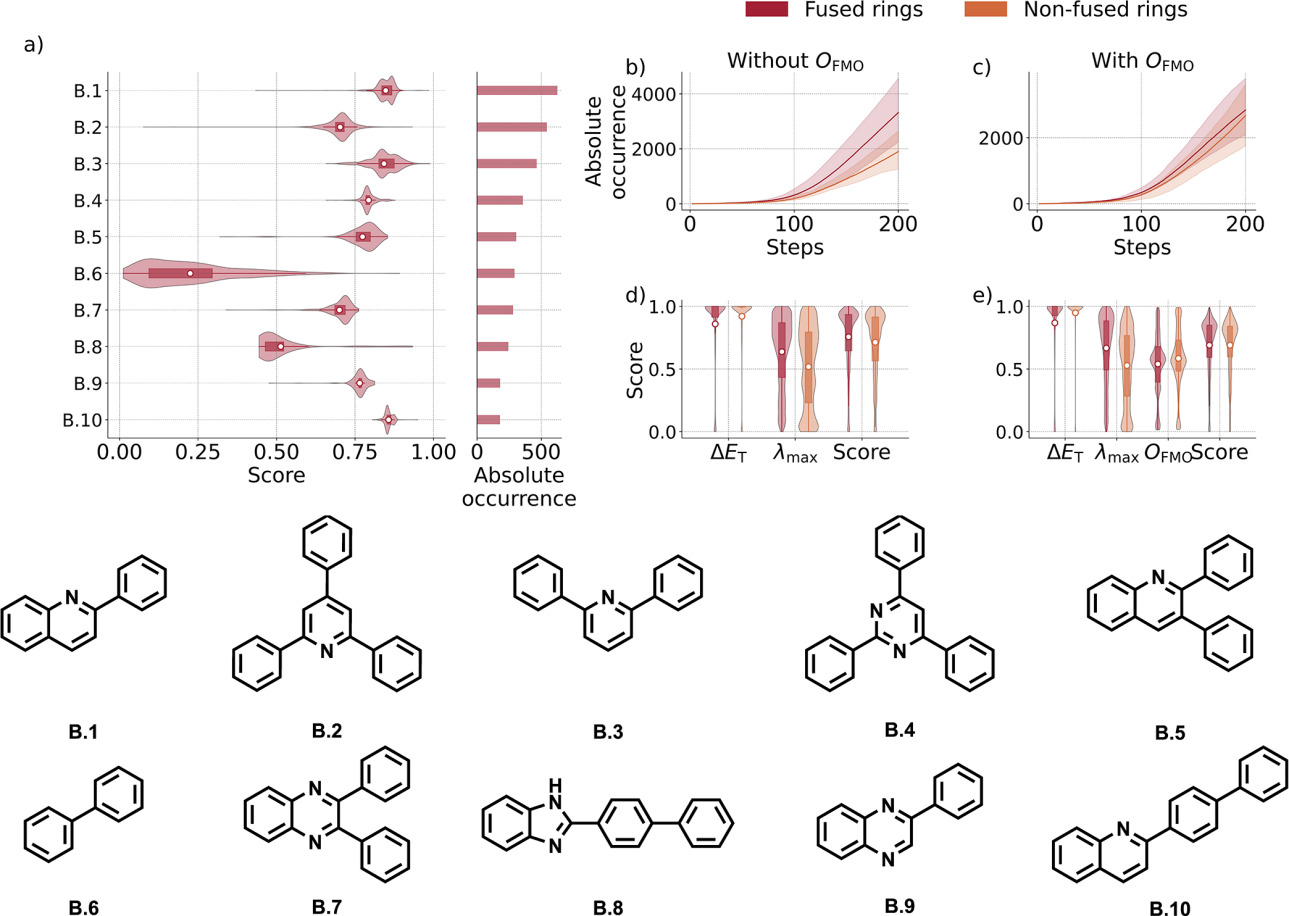
(a) Most occurring scaffolds over five independent runs using Δ*E*_T,_ λ_max_, *O*_FMO_, and θ_conjugation_ in the reward function. The distribution of scores obtained for molecules containing the respective scaffolds is indicated by violin plots, and white markers denote mean values. Moreover, the absolute occurrence per scaffold is represented in a bar diagram. (b–e) Evolution of the absolute occurrence of molecules containing fused and nonfused rings during the generative process, averaged over five independent runs, is shown in subplots. Results are shown (b) without inclusion of *O*_FMO_ in the reward function and (c) with *O*_FMO_. Solid lines represent the mean, while shaded areas indicate the minimum–maximum range observed across runs. (d, e) Distributions of molecular property scores (Δ*E*_T_, λ_max_, *O*_FMO_, and total score) for all generated molecules with score >0, again averaged over five runs. Panel (d) displays the results without *O*_FMO_, and panel (e) displays the results with *O*_FMO_. Violin plots indicate the distribution shape, while markers denote mean values.

### Computational Validation of Global Exploration and Local Exploration of Focused Chemical Space

With these efficient proxies to guide the design of organic PCs suitable to mediate EnT reactivity efficiently, we performed an exhaustive global exploration of chemical space, employing a multipronged approach by varying the composition of the scoring function to achieve optimal exploration with high diversity. Out of the vast number of generated molecules (2,031,964 molecules obtained in 103 generative runs, see Supporting Information for details), we identified 11,775 candidate molecules that met the criteria of Δ*E*_T_ > 60 kcal/mol, λ_max_ > 350 nm, and λ_max‑sTDA_ > 325 nm, respectively. Since the number of promising molecules exceeds the capacity of computational validation, a diverse set of candidates (432 molecules) was selected based on maximizing the predicted properties (Δ*E*_T_, λ_max_/λ_max‑sTDA_, and *O*_FMO_) as well as ensuring structural diversity by employing clustering using molecular fingerprint (Figure 4a, see Supporting Information). Using an automated computational workflow based on time-dependent density functional theory (TD-DFT) and excited-state dynamics as implemented in the ORCA software package,^38^ the photophysical properties (fluorescence rate (*k*_f_), ISC rate (*k*_ISC_), Δ*E*_T_, and λ_max_) of the candidates were determined. Surprisingly, the screening revealed many molecules with a low triplet energy (Δ*E*_T_ < 50 kcal/mol), all of which being diverse aza azulene derivatives. While high λ_max_ values are computed in line with the ML predictions of the generative model, the ML predicted values for Δ*E*_T_ show a large deviation (MAE: 28.0 kcal/mol) from the computed values.

**Figure 4:**
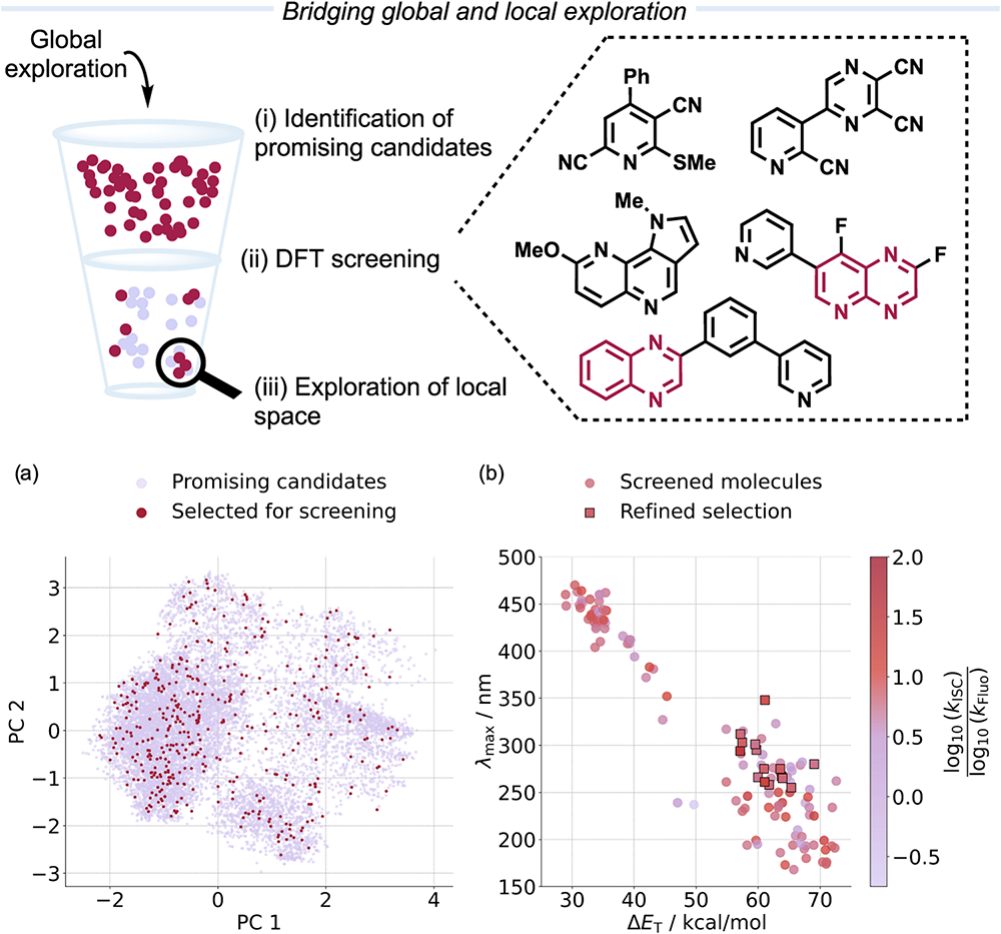
Schematic depiction of the workflow to bridge global and local exploration of chemical space. Promising candidates from the global exploration are selected (a) and screened using QM calculations (b). Analysis of the refined selection reveals promising structures for which the local space can be explored. The inset shows exemplary structures from the refined selection, and the bicyclic pyrazine scaffold is highlighted in red.

This exemplifies the challenge associated with using ML predictions for generative modeling, as high generalizability is required to provide reliable chemical space exploration. Despite the overestimation of Δ*E*_T_ for aza azulene derivatives, many candidates with a balanced set of properties and thus potentially suitable as PCs for EnT reactions could be identified. Property thresholds (Δ*E*_T_ > 55 kcal/mol, λ_max_ > 250 nm, $\frac{\log_{10}\left(k_{\mathrm{ISC}}\right)}{\log_{10}\left(k_{f}\right)}$ > 0.75, Figure 4b) were applied to refine the selection and identify the most promising candidates from the computational screening. The molecules in this refined selection contain diverse N-heteroarenes, such as substituted mono- and bicyclic pyridine, bicyclic pyrazine, or tricyclic naphthyridine derivatives. To the best of our knowledge, these scaffolds have not previously been considered as PCs for EnT catalysis. Notably, however, some scaffolds, such as cyanopyridine,^18^ cyanopyrazine,^39^ as well as quinoxaline,^40^ have previously been investigated for their application in photoredox catalysis.

This demonstrates how our global exploration strategy can lead to the identification of promising scaffolds with balanced photophysical properties for EnT catalysis. Rather than directly synthesizing and testing all molecules that were proposed by the generative model, global exploration targets the identification of promising scaffolds that can be further investigated in a localized exploration. This enables fine-tuning of the photocatalytic properties, and more importantly, to control the synthetic complexity, which is often a major challenge in generative modeling.^20^ While a variety of promising scaffolds emerged in the global exploration, the local exploration and subsequent experimental validation were focused on one exemplary scaffold. For this, bicyclic pyrazine derivatives were selected as promising scaffolds because of their prevalence among refined selections in global exploration (20% bicyclic pyrazine derivatives). Seminal reports about the application of quinoxaline and pyridopyrazine derivatives in optical materials,^41^ photoinitiators for polymerization,^42^ aggregation-induced reactive oxygen sensitization,^43^ as well as photoredox catalysis^40^ make it particularly intriguing to explore their aptitude for EnT catalysis. Owing to the robust and modular synthetic access from directly available building blocks, a tractable library was built up using six diamines and three diketones, affording 18 candidate PCs (Figure 5A). These candidates were subsequently evaluated using the same screening workflow based on QM calculations as that used for the global exploration.

**Figure 5:**
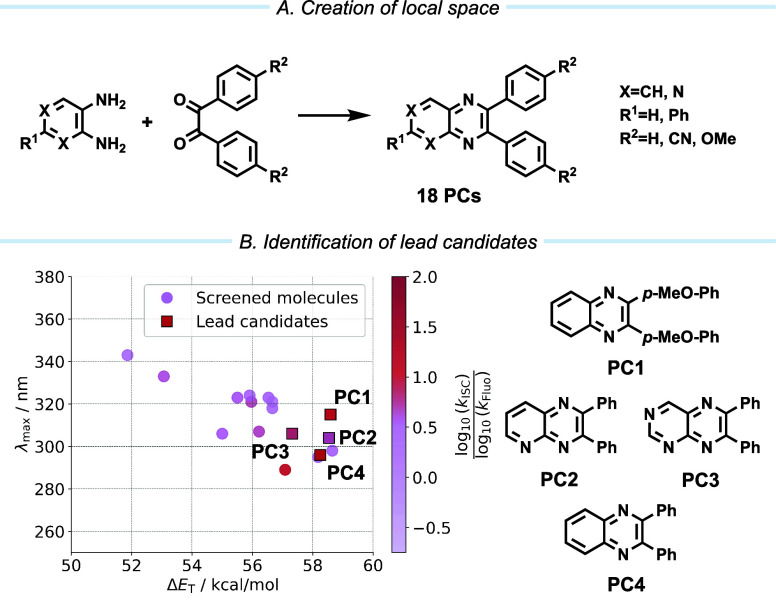
(A) For the construction of the local space, the bicyclic pyrazine scaffold was identified in the DFT screening, and a local space encompassing 18 PCs is created. (B) Computational exploration of this space yields the lead candidates (**PC1–4**) for further evaluation.

### Application of Selected Candidates

Out of the 18 screened candidates from the local bicyclic pyrazine space, four candidates with the most promising properties (Δ*E*_T_ > 57 kcal/mol, λ_max_ > 290 nm, $\frac{\log_{10}\left(k_{\mathrm{ISC}}\right)}{\log_{10}\left(k_{f}\right)}$ > 0.65) were synthesized to probe their aptitude to enable EnT-mediated reactivity (Figure 5B). All PCs could be obtained in good to excellent yields in a single step from commercially available starting materials. UV–vis absorption studies showed that all PCs, except for **PC4**, showed extinction in the visible range ( > 400 nm, see Supporting Information). Next, to probe the molecules’ ability to enable EnT-mediated reactivity, the photoisomerization of *trans*-methyl cinnamate (**1a**) to the *cis*-isomer (**1b**) was first investigated as a well-studied intramolecular reaction (Figure 6).^2^ All PCs yielded a mixture of *cis*/*trans-*isomers, indicating the successful sensitization of **1a**. No significant isomerization was observed in the absence of a PC, ruling out thermal isomerization or isomerization through direct excitation.

**Figure 6:**
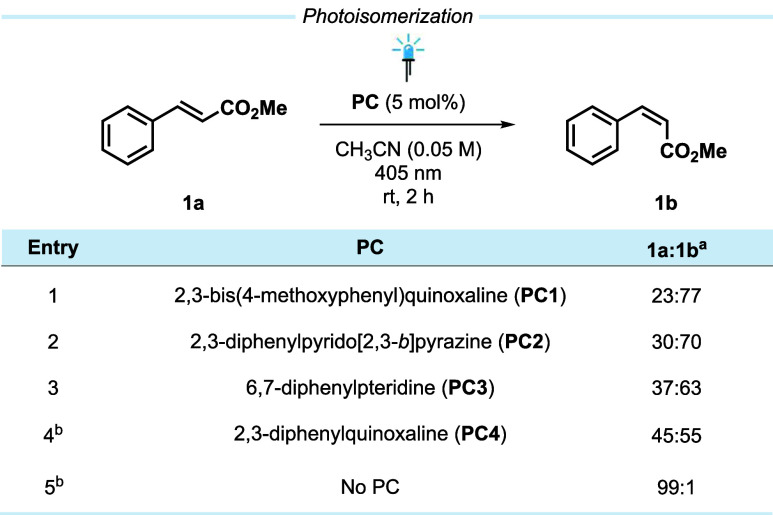
Photoisomerization of *trans*-methyl cinnamate (**1a**) to *cis*-methyl cinnamate (**1b**) employing **PC1–4** (^a^ ratio determined by ^1^H NMR of the crude reaction mixture, ^b^ 365 nm used for excitation).

Inspired by these promising results, we turned our focus to more challenging intermolecular EnT-mediated reactivity.

For this, the [2+2]-photocycloaddition of phenyl maleimide (**2**) with styrene (**3**) was investigated using the newly designed PCs (Figure 7A). Due to the absorbance of **2** in the UV region (< 400 nm), however, **PC4** was not employed for this reaction, as direct excitation of **2** would compete with EnT from **PC4**. Pleasingly, **PC1**, **PC2**, and **PC3** yielded the product **4** in moderate to good yields (80%, 68%, and 46%, respectively), demonstrating their aptitude as effective PCs. This catalytic performance surpasses the results of the original report by Kokotos et al., who used thioxanthone as the PC (75% yield of **4**),^44^ showing the capacity of our method to discover PCs to challenge the state-of-the-art.

**Figure 7:**
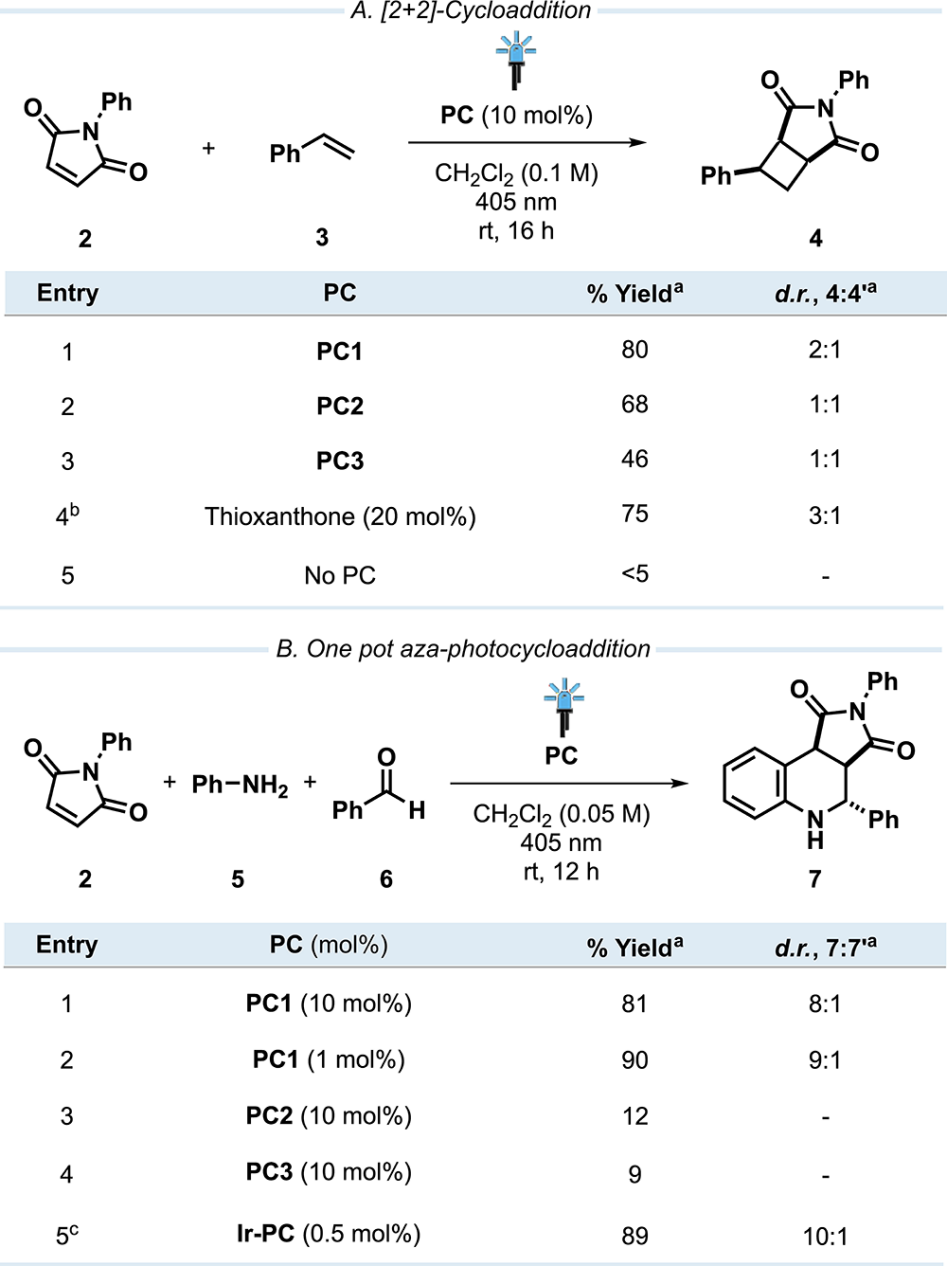
(A) [2+2]-Photocycloaddition of phenyl maleimide (**2**) with styrene (**3**) employing **PC1–3**. (B) One-pot aza-photocycloaddition employing **PC1–3** (^a^ determined by ^1^H NMR analysis using CH_2_Br_2_ as an internal standard, ^b^ results taken from the original reports of Kokotos et al. (CH_2_Cl_2_ (0.1 M), 440 nm, 16 h),^44^ ^c^ and Liu et al. (CH_2_Cl_2_ (0.05 M), 405 nm, 8 h, isolated yield),^45^ **Ir-PC**: [Ir­(dF­(CF_3_)­ppy)_2_(ppzpy)]­PF_6_).

Subsequently, the aza-photocyclization of **2** with *N*-aryl imine to yield the tetrahydroquinoline derivative (**7**) was investigated. In the original report, Liu et al. emphasized that with commonly used transition-metal-based PCs, such as Ir­(ppy)_3_ (0%), and Ir­[dF­(CF_3_)­ppy]_2_(dtbbpy)­PF_6_ (66%), as well as the organic PCs thioxanthone (54%), benzophenone (0%), and 4CzIPN (17%), only poor to moderate yields of **7** could be obtained.^45^ By tailoring the ligands of Ir-based PCs to the reaction, they were able to increase the yield of **7** to 93%. Due to the mediocre performance of commonly employed PCs, we were intrigued to investigate the catalytic performance of our PCs and performed the reaction in a one-pot process with *in situ* formation of the imine. While **PC2** and **PC3** did not enable efficient reactivity (12% and 9% yields, respectively), using **PC1** enabled the formation of **7** in a high yield (90%, Figure 7B). This makes the performance of **PC1** comparable to the best-performing Ir-based PC from Liu et al., where **7** is obtained in 89% yield in the one-pot reaction. Again, our PCs are able to match or exceed the current state-of-the-art, demonstrating the promise of the inverse design approach.

Overall, these results exemplify that our proposed inverse design strategy enables the discovery of efficient EnT PCs. Owing to the hybrid approach of global exploration followed by focused structure refinement, data-driven molecular designs could be directly translated into experimentally testable candidates using a one-step reaction, thereby overcoming challenges associated with synthesizability, which represents a major bottleneck in generative modeling. Remarkably, all synthesized structures **PC1–4** demonstrated their aptitude to promote efficient EnT reactivity, underscoring the potential of the inverse design strategy as a powerful route to accelerate the discovery of novel or underexplored PCs.

## Conclusions

In this work, we developed a data-driven inverse design strategy for the discovery of organic PCs tailored to EnT-mediated reactions. Our approach encompasses a global exploration strategy, leveraging the REINVENT generative AI framework that guides the chemical space navigation by reinforcement learning using ML predictions and semiempirical proxies of photocatalytic properties as the reward. This allowed for a broad exploration of chemical space, leading to the rediscovery of known PC scaffolds, like thioxanthone, which acted as a validation of the workflow to identify efficient PCs. Moreover, by setting structural constraints to guide the model away from the well-explored regions of PC space, unprecedented structures with suitable properties to mediate EnT reactivity could be identified. A subsequent local exploration allows to refine the photocatalytic properties using (TD)-DFT calculations and control the molecules’ synthesizability. The success and direct experimental applicability of this hybrid strategy are successfully demonstrated for bicyclic pyrazine derivatives. Four promising PCs were successfully synthesized in one-step reactions, and their catalytic performances in three different EnT-mediated reactions matched or exceeded state-of-the-art organic PCs.

Taken together, these results underscore the utility and power of our proposed inverse design strategy, which effectively bridges computational discovery with experimental validation. By combining global exploration with targeted local optimization, our framework offers an efficient and readily applicable pathway to identifying and evaluating organic PCs for EnT catalysis. This demonstrates how generative modeling can augment chemists’ creativity in molecular design and enable the exploration of uncharted chemical space to push the boundaries of photocatalysis. We anticipate that the ongoing development of more efficient and accurate proxies to predict photocatalytic properties, particularly *Φ*_ISC_, will further enhance the utility of the proposed strategy by enabling both more accurate and efficient chemical space exploration.^46^ While this study is focused on EnT catalysis, the workflow can be readily expanded to related applications, such as the design of organic photoredox PCs, or optical materials by integrating suitable proxies, e.g., redox potentials or reverse ISC rates (*k*_rISC_).

## Supplementary Material



## Data Availability

The code for the generative model and all generated molecules can be found at https://github.com/le-schlo/InvEnT and deposited on Zenodo: 10.5281/zenodo.18215365. Different artists at flaticon.com are acknowledged for the icons used in Figures 1, 6, and 7 and the graphical abstract.

## Notes

Additional details of the generative model, description of methods used for the quantum mechanical calculations, and experimental details, materials, and methods. (PDF)
