# Supplementary material for: Inverse Molecular Design for the Discovery of Organic Energy Transfer Photocatalysts: Bridging Global and Local Chemical Space Exploration
Source: J Am Chem Soc. 2026 Feb 6;148(6):6451–61. doi: 10.1021/jacs.5c20087 (PMC12921867; doi:10.1021/jacs.5c20087)
Supplement: Supplementary file 1 [file ja5c20087_si_001.pdf]

Supplementary Information

Inverse Molecular Design for the Discovery of Organic Energy Transfer Photocatalysts: Bridging Global and Local Chemical Space Exploration

Leon Schlosser,[1,2] Nils Rendel,[1] Julius Gemen,[1] Frank Glorius,[1*] and Kjell Jorner[2,3*]

[1]Organisch-Chemisches Institut, Universität Münster, Münster, Germany.

[2]Institute of Chemical and Bioengineering, Department of Chemistry and Applied Biosciences, ETH Zurich, Vladimir-Prelog-Weg 1, Zürich, Switzerland.

[3]NCCR Catalysis, Switzerland

*Correspondence to: glorius@uni-muenster.de, kjell.jorner@chem.ethz.ch

TABLE OF CONTENTS

1. GENERATIVE MODEL ... 3
1.1. Model details ... 3
1.2. Scoring components ... 4
1.2.1. Triplet energy prediction ... 4
1.2.2. Maximum absorption wavelength prediction ... 6
1.2.3. FMO analysis ... 8
1.2.4. Degree of conjugation ... 17
1.2.5. Rigidity ... 17
1.2.6. SA score ... 17
1.3. Aggregation functions ... 18
1.4. Production runs ... 18
1.4.1. Rediscovery ... 18
1.4.2. Chemical space exploration ... 19
1.4.3. Evaluation of O_{FMO} ... 20
1.4.4. Overview of run parameters ... 22
1.5. Candidate selection ... 51
2. QUANTUM MECHANICAL CALCULATIONS ... 67
2.1. Validation of predicted ISC yield ... 68
2.2. Automated workflow ... 69
3. LOCAL EXPLORATION ... 76
3.1. Virtual library ... 76
4. GENERAL EXPERIMENTAL ... 78
4.1. Glassware, Solvents and Reagents ... 78
4.2. Photochemical set-up and light sources ... 78
4.3. Chromatography and Data Analysis ... 79
4.4. Naming of Compounds ... 79
5. EXPERIMENTAL DATA ... 80
5.1. Synthesizing of the photocatalysts ... 80
5.2. Establishing the photocatalyst ... 83
5.2.1. Photoisomerization ... 83
5.2.2. [2+2]-Cycloaddition ... 83
5.2.3. One pot aza-photocycloaddition ... 85
5.3. UV/vis Absorption Spectroscopy ... 86
5.4. Cyclic Voltammetry ... 87
6. SPECTROSCOPIC DATA ... 90
7. REFERENCES ... 97

1. GENERATIVE MODEL

1.1. Model details

The default prior ("agent") of REINVENT for *de novo* molecular generation was used for this work. Details of the algorithm can be found in the original publication.[1] For all runs, the SMILES were internally randomized and a batch size of 64 was used. The agent was optimized using the Difference between Augmented and Posterior (DAP) strategy, where a loss is computed as the difference between the agent's likelihood and the augmented likelihood (prior likelihood combined with a reward scaled by the user-defined parameter σ), guiding the agent to generate molecules that balance chemical plausibility and task-specific optimization. In all experiments, σ was set to 128 and the learning rate to 0.0001. A scaffold-based diversity filter was applied during reinforcement learning to promote chemical diversity and avoid mode collapse. Specifically, a *ScaffoldSimilarity* diversity filter was used with a bucket size of 100 compounds and a minimum reward threshold of 0.4, meaning only molecules scoring above this threshold were added to the diversity memory. Molecules with a scaffold similarity above 0.4 were grouped together, and new molecules falling into full buckets were assigned a score of zero. Molecular scaffolds were generated by decomposing each molecule into ring systems, linkers, and side chains. Linkers were defined as non-ring paths connecting two ring systems, while side chains comprised all remaining non-ring atoms. The scaffold was defined as the union of ring systems and linkers. This helped maintain scaffold diversity in the generated molecules throughout training. The reinforcement learning optimization was carried out in a single stage and terminated after 250 iterations.

During production runs, certain structural motives were penalized to avoid unwanted synthetic complexity and undesired reactivity. **Table S1** gives an overview of the penalized substructure in terms of their SMARTS patterns. Generated molecules that match any of these SMARTS get assigned a score of zero.

Table S1: Overview of SMARTS that are penalized in the generative run.

SMARTS	Description	SMARTS	Description
[*;r3-4,r7-17]	3-4-, and 7-17-membered rings	C=C	Exclude non-aromatic carbon-carbon double bonds
[N;X3]	Exclude aliphatic amines	[!#6][!#6]	Exclude bonds between two non-carbon atoms
[#6]=O	Exclude carbonyl	[CH2]	Exclude aliphatic carbons with two hydrogens
[OH]	Exclude alcohols	[r](=N)	Exocyclic imines

Besides the pretrained agent from the original REINVENT publication, two other agents were trained based on a subset of the ZINC dataset and a specialized dataset on TADF-based molecules. For the subset of the ZINC22 database,[2] all molecules up to 24 heavy atoms were compiled and filtered for a degree of conjugation > 0.7 and the presence of at least one aromatic ring. From each reduced tranche of the dataset, a maximum of 1000 molecules were selected randomly to compile the final dataset (707,490 molecules) to train the agent for 75 epochs with a batch size of 64 using a 70:30 train/validation random split. Moreover, a specialized agent

finetuned for TADF-based molecules was trained. For this, a dataset for finetuning was compiled from Cole *et al.*[3] (1,318 molecules) and a randomly sampled subset (50 molecules) of the dataset published by Cooper *et al.*[4] which yielded the final finetuning set (1,368 molecules). Starting from the default agent, the specialized agent was trained for 60 epochs with a batch size of 64 using a 70:30 train/validation random split of the finetuning set.

1.2. Scoring components

1.2.1. Triplet energy prediction

For the prediction of triplet energies with EnTdecker, the Chemprop model was used as reported in the original publication.[5] Since the underlying training data of EnTdecker are adiabatic triplet energies using DFT calculations, the model's performance was additionally investigated on a benchmark set of experimental triplet energies. The benchmark set consists of a diverse set of molecules, including many important chromophores, as well as organic photocatalysts such as thioxanthone.[6] The triplet energies were predicted with a MAE of 4.33 kcal/mol and R^2 of 0.766 compared to the experimental values (**Figure S1**). Importantly, the greatest deviations are observed for aromatic polycycles, such as porphyrin, phthalocyanine and pentacene, which are outside the training domain of EnTdecker. However, due to their low triplet energies, these structures are less important for the desired application of identifying efficient organic PC, as generally PCs with high triplet energies enable a wider variety of transformations.

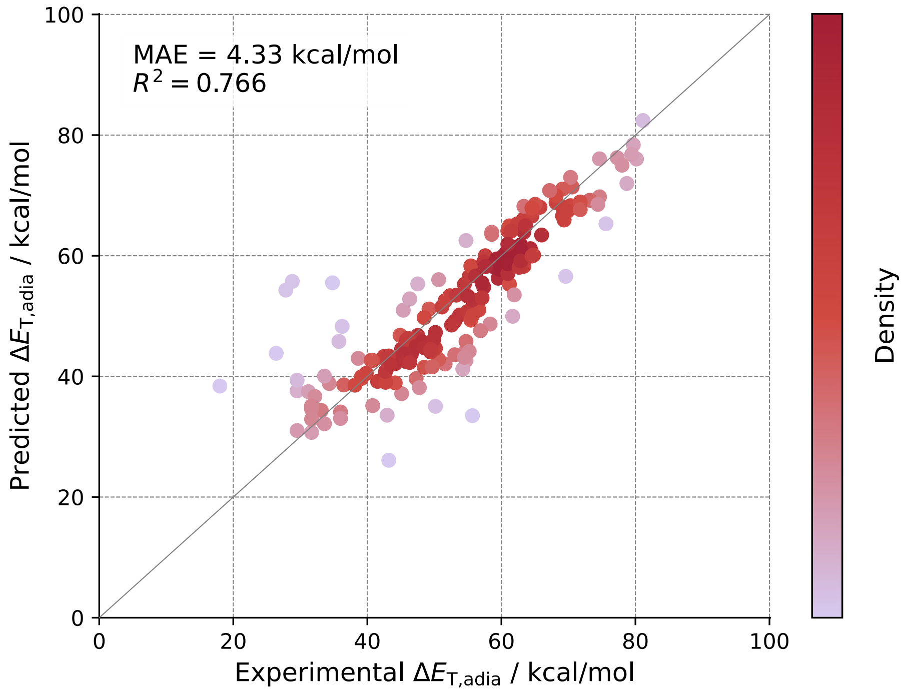

Figure S1: Parity plot of predicted triplet energies with EnTdecker and the experimental triplet energies (202 molecules).

To probe the predictive accuracy of EnTdecker on a set of organic PCs outside the training domain, two families of thermally activated delayed fluorescence (TADF) based PCs (carbazolyl-cyanobenzenes,

imidazophenothiazines) with reported triplet energies were evaluated.[7,8] EnTdecker predicted the triplet energies for this set of 19 molecules with an MAE of 4.86 kcal/mol. Notably, this set exclusively contains molecules with far more atoms (34 – 73 atoms) than in the training data of EnTdecker (up to 25 atoms).

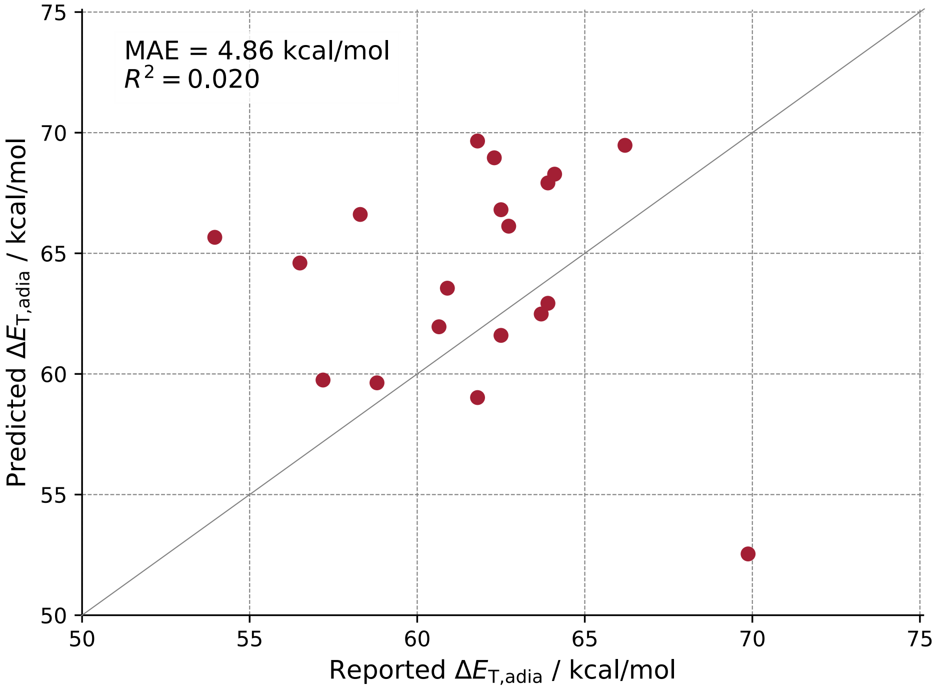

Figure S2: Parity plot of predicted triplet energies with EnTdecker and the reported triplet energies of TADF molecules derivatives (19 molecules).

It was further investigated if substitution effects of thioxanthone derivatives can be captured by EnTdecker.[9] While a systematic underestimation of around 3 kcal/mol is observed (MAE = 3.12 kcal/mol; MSE = 3.12 kcal/mol) a very high correlation of the predicted and the experimentally obtained triplet energies is observed (R^2: 0.954).

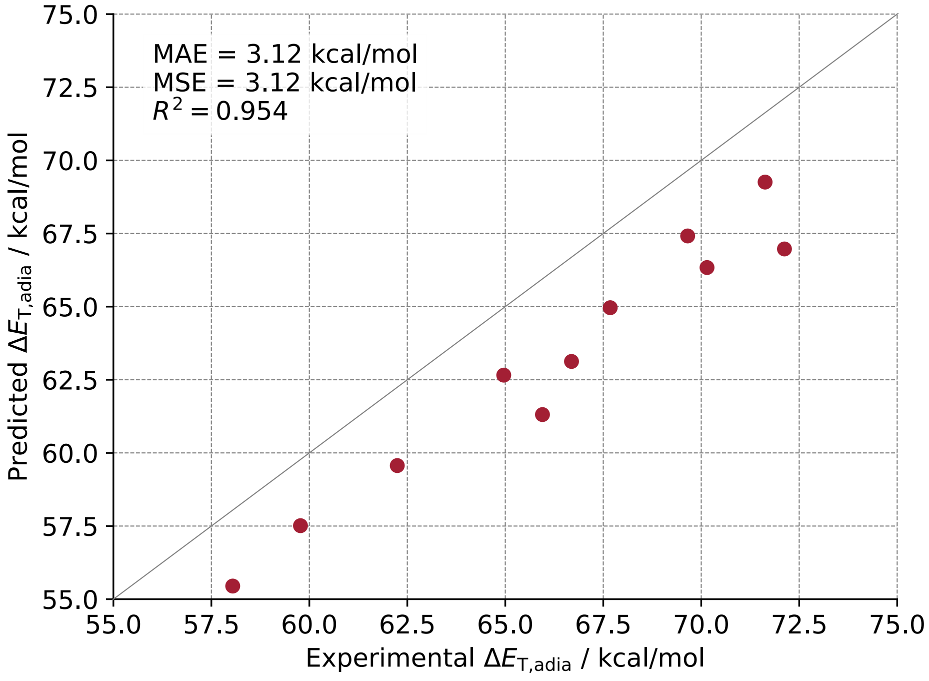

Figure S3: Parity plot of predicted triplet energies with EnTdecker and the experimental triplet energies of thioxanthone derivatives (11 molecules).

All benchmark sets are available in the GitHub repository.[10]

1.2.2. Maximum absorption wavelength prediction

Machine-learning based prediction

For the prediction of the maximum absorption wavelength, the models developed by Gómez-Bombarelli et al. are employed. In a first stage the vertical S_1 energy is predicted with an ensemble of models as available in the Zenodo repository.[11] This value gets averaged and serves as an input to the second ensemble of models which predicts the maximum absorption wavelengths in acetonitrile.

Computational prediction

Absorption properties were calculated using the semi-empirical extended tight-binding (xTB) method combined with the simplified Tamm–Dancoff approximation (sTDA).[12,13] Molecular structures were generated from SMILES strings using RDKit. The conformational ensemble average was approximated by the molecule's lowest energy conformer using the ETKDGv3 algorithm and the MMFF94 forcefield.[14] The structure was further optimized using GFN-FF.[15] Vertical excitation energies were then computed with sTDA. From the resulting output, excitation wavelengths and oscillator strengths were extracted. Each transition was broadened using Gaussian functions, simulating the absorption spectrum. The maximum absorption wavelength was obtained using a threshold for peak identification of 0.00001.

This computational approach was evaluated on a benchmark set of 1,239 experimentally determined maximum absorption wavelengths in acetonitrile (**Figure S4**). The benchmark set was obtained from Gómez-Bombarelli *et al.*[16]

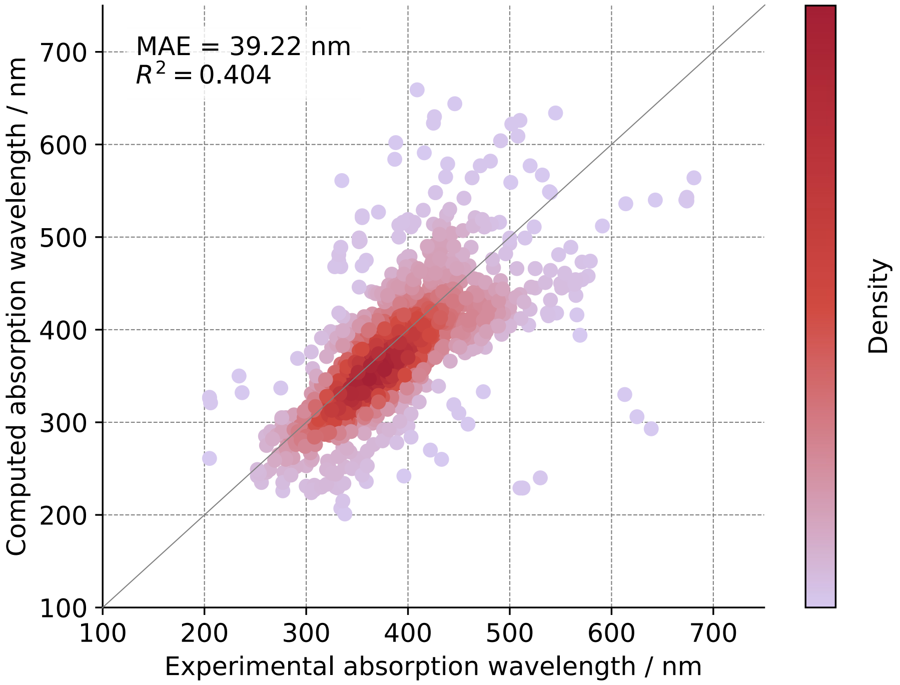

Figure S4: Parity plot of computed maximum absorption wavelength and experimental values (1,239 molecules in acetonitrile).

Evaluation on literature data

Two literature datasets were compiled to assess the predictive performance of both approaches on a common molecular scaffold. For a series of thioxanthone derivatives (**Figure S5**), the reported maximum absorption wavelengths were compared with predictions from the ML model and sTDA calculations. A similar comparison was carried out for a set of 20 indole derivatives (**Figure S6**).[17]

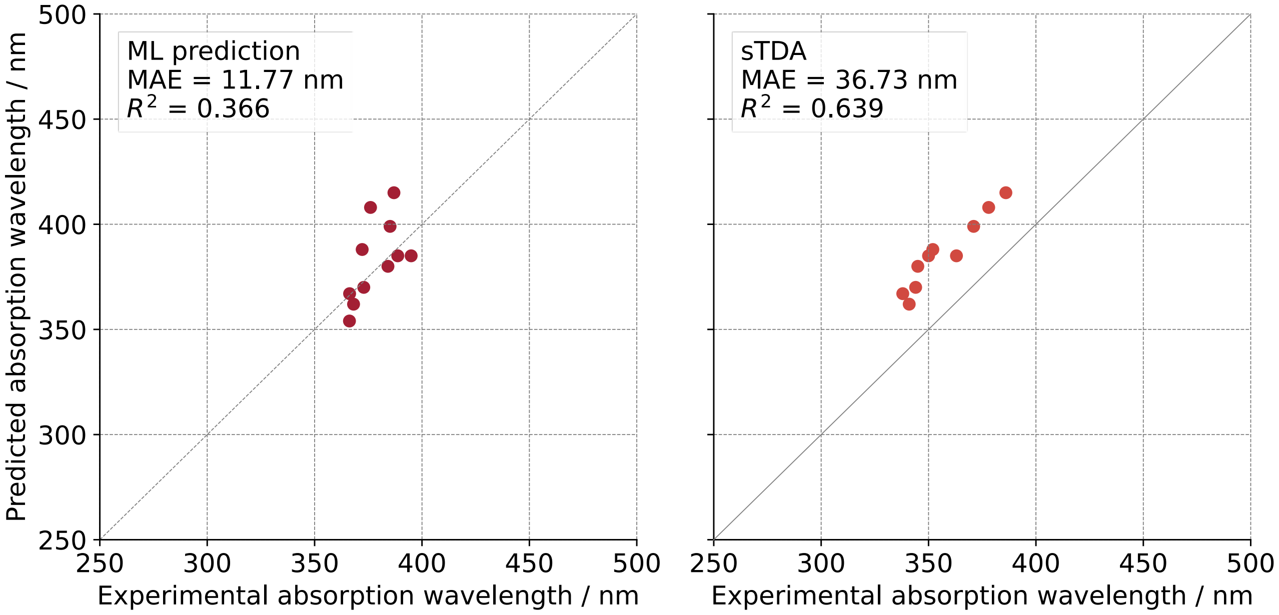

Figure S5: Parity plot of reported maximum absorption wavelength and values obtained with the ML model (left) and computed with sTDA (right) for a set of 11 thioxanthone derivatives.

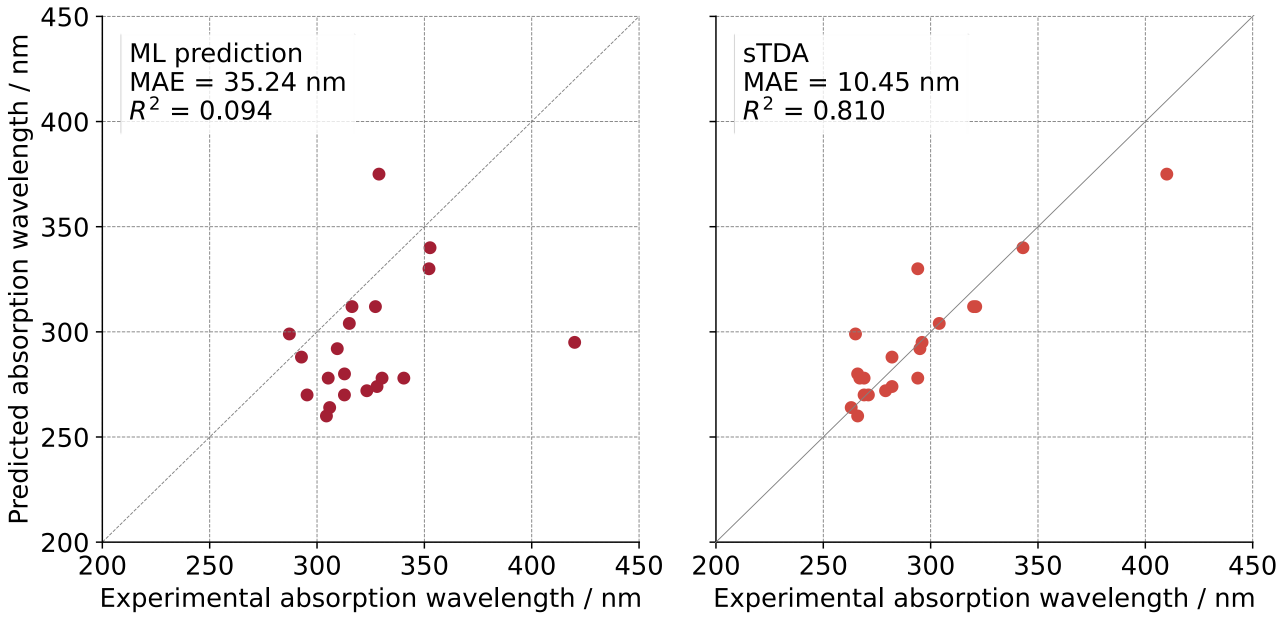

Figure S6: Parity plot of reported maximum absorption wavelength and values obtained with the ML model (left) and computed with sTDA (right) for a set of 20 indoles derivatives.

1.2.3. FMO analysis

Optimized structures in singlet and triplet multiplicities were obtained using xTB according to the workflow for computing the maximum absorption wavelength (*vide supra*). Frontier molecular orbitals were computed using GFN2-xTB and orbital overlap analysis was performed with Multiwfn 3.7.[18]

Estimation of singlet-triplet gap $\Delta E_{S_1-T_1}$

A benchmark set of 50 molecules with reported $\Delta E_{S_1-T_1}$ was compiled from literature (**Table S2**).[8,19–22] The parity plot between the $\Delta E_{S_1-T_1}$ and the HOMO-LUMO overlap (O_{FMO}) showed a moderately strong correlation (**Figure S7**, R^2: 0.631).

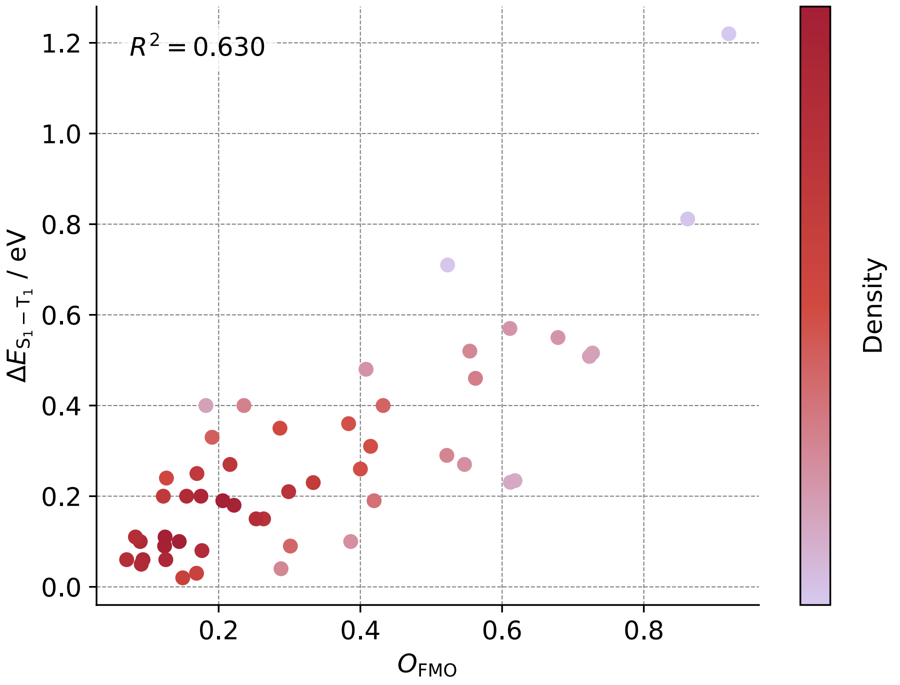

Figure S7: Correlation between $\Delta E_{S_1-T_1}$ and HOMO-LUMO overlap (O_{FMO}) for a benchmark set (50 molecules).

Table S2: Benchmark set of molecules with reported $\Delta E_{S_1-T_1}$ and computed HOMO-LUMO overlap (O_{FMO}).

Molecule	SMILES	$\Delta E_{S_1-T_1}$ (eV)	O_{FMO}
NPh3[20]	C1(N(C2=CC=CC=C2)C3=CC=CC=C3)=CC=CC=C1	0.57	0.61
ACRFLCN[20]	N#CC(C=C1)=CC2=C1C3=C(C24C(C=CC=C5)=C5N(C6=CC=CC=C6)C7=C4C=CC=C7)C=C(C#N)C=C3	0.24	0.13
CBP[20]	C1(N(C2=CC=C(C3=CC=C(N(C4=C5C=CC=C4)C6=C5C=CC=C6)C=C3)C=C2)C7=C8C=CC=C7)=C8C=CC=C1	0.71	0.52
2PXZ-OXD[20]	C1(OC(C=CC=C2)=C2N3C4=CC=C(C5=NN=C(C(C=C6)=CC=C6N7C8=C(C=CC=C8)OC9=C7C=CC=C9)O5)C=C4)=C3C=CC=C1	0.15	0.26
DPA-DPS[20]	O=S(C1=CC=C(N(C2=CC=CC=C2)C3=CC=CC=C3)C=C1)(C4=CC=C(N(C5=CC=CC=C5)C6=CC=CC=C6)C=C4)=O	0.52	0.55
SpiroCN[20]	CC(C=C1)=CC=C1N(C2=CC=C(C)C=C2)C3=CC(C4=CC=C(N(C5=CC=C(C)C=C5)C6=CC=C(C)C=C6)C=C4C78C9=CC(C#N)=CC=C9C%10=C7C=C(C#N)C=C%10)=C8C=C3	0.06	0.07
CC2TA[20]	C1(N(C2=C3C=CC=C2)C4=C3C=C(N(C5=C6C=CC=C5)C7=C6C=CC=C7)C=C4)=NC(C8=CC=CC=C8)=NC(N(C9=C%10C=CC=C9)C%11=C%10C=C(N(C%12=C%13C=CC=C%12)C	0.2	0.15

	%14=C%13C=CC=C%14)C=C%11)=N1		
4CzIPN[20]	N#CC1=C(N2C3=CC=CC=C3C4=CC=CC=C42)C(N5C6=CC=CC=C6C7=CC=CC=C75)=C(N8C9=CC=CC=C9C%10=CC=CC=C%108)C(C#N)=C1N%11C(C=CC=C%12)=C%12C%13=C%11C=CC=C%13	0.1	0.14
2CzPN[20]	N#CC1=CC(N2C3=C(C4=C2C=CC=C4)C=CC=C3)=C(N5C6=C(C=CC=C6)C7=C5C=CC=C7)C=C1C#N	0.31	0.41
PhCz[20]	C1(N(C2=CC=CC=C2)C3=C4C=CC=C3)=C4C=CC=C1	0.55	0.68
CzT[20]	C1(N(C2=C3C=C(C4=CC5=C(N(C6=CC=CC=C6)C7=C5C=CC=C7)C=C4)C=C2)C8=C3C=CC=C8)=NC(C9=CC=CC=C9)=NC(C%10=CC=CC=C%10)=N1	0.1	0.09
DMAC-DPS[20]	O=S(C1=CC=C(N2C3=CC=CC=C3C(C)(C)C4=C2C=CC=C4)C=C1)(C5=CC=C(N6C7=CC=CC=C7C(C)(C)C8=C6C=CC=C8)C=C5)=O	0.09	0.12
DTPA-DPS[20]	O=S(C1=CC=C(N(C2=CC=C(C(C)(C)C)C=C2)C3=CC=C(C(C)(C)C)C=C3)C=C1)(C4=CC=C(N(C5=CC=C(C(C)(C)C)C=C5)C6=CC=C(C(C)(C)C)C=C6)C=C4)=O	0.46	0.56
4CzTPN[20]	N#CC1=C(N2C3=C(C4=C2C=CC=C4)C=CC=C3)C(N5C6=C(C=CC=C6)C7=C5C=CC=C7)=C(C#N)C(N8C9=C(C%10=C8C=CC=C%10)C=CC=C9)=C1N%11C%12=C(C%13=C%11C=CC=C%13)C=CC=C%12	0.09	0.30
DTC-DPS[20]	O=S(C1=CC=C(N2C3=CC=C(C(C)(C)C)C=C3C4=C2C=CC(C(C)(C)C)=C4)C=C1)(C5=CC=C(N6C7=CC=C(C(C)(C)C)C=C7C8=C6C=CC(C(C)(C)C)=C8)C=C5)=O	0.36	0.38
PXZ-TRZ[20]	C1(C2=CC=C(N3C4=C(C=CC=C4)OC5=C3C=CC=C5)C=C2)=NC(C6=CC=CC=C6)=NC(C7=CC=CC=C7)=N1	0.06	0.09
4CzPN[20]	N#CC1=C(N2C3=C(C4=C2C=CC=C4)C=CC=C3)C(N5C6=C(C=CC=C6)C7=C5C=CC=C7)=C(N8C9=C(C%10=C8C=CC=C%10)C=CC=C9)C(N%11C%12=C(C=CC=C%12)C%13=C%11C=CC=C%13)=C1C#N	0.15	0.25
PXZ-TDZ[20]	C1(OC(C=CC=C2)=C2N3C4=CC=C(C5=NN=C(C(C=C6)=CC=C6N7C8=C(C=CC=C8)OC9=C7C=CC=C9)S5)C=C4)=C3C=CC=C1	0.11	0.08
PIC-TRZ[20]	C1(N(C2=C3C=CC4=C2N(C5=CC=CC=C5)C6=C4C=CC=C6)C7=C3C=CC=C7)=NC(C8=CC=C(C9=CC=CC=C9)C=C8)=NC(N(C%10=C%11C=CC%12=C%10N(C%13=CC=CC=C%13)C%14=C%12C=CC=C%14)C%15=	0.18	0.22

	C%11C=CC=C%15)=N1		
DMOC-DPS[20]	O=S(C1=CC=C(N2C3=CC=C(OC)C=C3C4=C2C=CC(OC)=C4)C=C1)(C5=CC=C(N6C7=CC=C(OC)C=C7C8=C6C=CC(OC)=C8)C=C5)=O	0.21	0.30
2PXZ-TAZ[20]	C1(OC(C=CC=C2)=C2N3C4=CC=C(C5=NN=C(C(C=C6)=CC=C6N7C8=C(C=CC=C8)OC9=C7C=CC=C9)N5C%10=CC=CC=C%10)C=C4)=C3C=CC=C1	0.23	0.33
PhCzTAZ[20]	C1(C2=CC=CC=C2)=NC(C3=CC=CC=C3)=NC(C4=CC=CC=C4C5=C(C6=CC=C(N(C7=CC=CC=C7)C8=C9C=CC=C8)C9=C6)C=CC=C5)=N1	0.2	0.12
DACQ[20]	C1(N(C2=CC=CC=C2)C3=CC=CC=C3)=CC4=C(N(C5=CC=C(C6=NC(C=CC=C7)=C7N=C6)C=C5)C8=C4C=C(N(C9=CC=CC=C9)C%10=CC=CC=C%10)C=C8)C=C1	0.08	0.18
PXZQ[20]	C12=CC=CC=C1N=C(C3=CC=C(N4C5=C(C=CC=C5)OC6=C4C=CC=C6)C=C3)C=N2	0.19	0.21
DPA-AQ[20]	O=C1C2=C(C=CC(N(C3=CC=CC=C3)C4=CC=CC=C4)=C2)C(C5=CC(N(C6=CC=CC=C6)C7=CC=CC=C7)=CC=C51)=O	0.27	0.55
BBPA-AQ[20]	O=C1C2=C(C=CC(N(C3=CC=C(C4=CC=CC=C4)C=C3)C5=CC=C(C6=CC=CC=C6)C=C5)=C2)C(C7=CC(N(C8=CC=C(C9=CC=CC=C9)C=C8)C%10=CC=C(C%11=CC=CC=C%11)C=C%10)=CC=C71)=O	0.26	0.40
DTC-AQ[20]	O=C1C2=C(C=CC(N3C4=C(C5=C3C=CC(C(C)(C)C)=C5)C=C(C(C)(C)C)C=C4)=C2)C(C6=CC(N7C8=C(C9=C7C=CC(C(C)(C)C)=C9)C=C(C(C)(C)C)C=C8)=CC=C61)=O	0.19	0.42
DMAC-AQ[20]	O=C1C2=C(C=CC(N(C3=C4C=CC=C3)C5=C(C4(C)C)C=CC=C5)=C2)C(C6=CC(N(C7=C8C=CC=C7)C9=C(C8(C)C)C=CC=C9)=CC=C61)=O	0.11	0.12
ACRSA[20]	O=C(C1=C2C=CC=C1)C3=CC=CC=C3C42C5=CC=CC=C5N(C6=CC=CC=C6)C7=C4C=CC=C7	0.03	0.17
AcPmBPX[20]	CC1(C)C2=C(C=CC=C2)N(C3=CC=C(C4=CC(C(C5=CC=C(C)C=C5)=O)=C(C6=CC=C(N(C7=C8C=CC=C7)C9=C(C8(C)C)C=CC=C9)C=C6)C=C4C(C%10=CC=C(C)C=C%10)=O)C=C3)C%11=C1C=CC=C%11	0.05	0.09
PxPmBPX[20]	O=C(C1=CC=C(C)C=C1)C2=CC(C3=CC=C(N(C4=C5C=CC=C4)C6=C(O5)C=CC=C6)C=C3)=C(C(C7=CC=C(C)C=C7)=O)C=C2C8=CC=C(N(C9=C%10C=CC=C9)C%11=C(O%10)C=CC=C%11)C=C8	0.02	0.15

TX1[19]	O=C1C2=C(C=CC=C2)SC3=CC=CC=C31	0.23433	0.62
TX8[19]	O=C1C2=C(C=CC=C2)SC3=CC(C)=CC=C31	0.23061	0.61
TX2[19]	O=C1C2=C(C(C=CC=C3)=C3C=C2)SC4=CC=CC=C41	0.50834	0.72
TX9[19]	O=C1C2=C(C(C=CC=C3)=C3C=C2)SC4=CC(C)=CC=C41	0.51577	0.73
TX3[19]	O=C1C2=C(C(C=C(C=CC=C3)C3=C4)=C4C=C2)SC5=CC=CC=C51	0.81148	0.86
p-Icz-PI[21]	CC(C)(C)C1=CC2=C(C=C1)N3C4=C2C=C(C(C)(C)C)C=C4C5=C3C=C6C(C(N(C)C6=O)=O)=C5	0.29	0.52
o-Icz-PI[21]	CC(C)(C)C1=CC2=C(C3=C1)N(C4=CC=C(C(N(C)C5=O)=O)C5=C43)C6=C2C=C(C(C)(C)C)C=C6	0.04	0.29
Cz-PI[21]	CC(C)(C1=CC2=C(N(C3=C2C=C(C=C3)C(C)(C)C)C4=CC=C(C5=C4)C(N(C5=O)C)=O)C=C1)C	0.1	0.39
ACR-IPTZ[8]	CC(C1=C2C=CC=C1)(C)C3=C(C=CC=C3)N2C(C=C4)=CC=C4C5=NC6=CC=CC7=C6N5C8=CC=CC=C8S7	0.2	0.17
ACR-IPTZO[8]	CC(C1=C2C=CC=C1)(C)C3=C(C=CC=C3)N2C(C=C4)=CC=C4C5=NC6=CC=CC7=C6N5C8=CC=CC=C8S7=O	0.25	0.17
ACR_IPTZO2[8]	CC(C1=C2C=CC=C1)(C)C3=C(C=CC=C3)N2C(C=C4)=CC=C4C5=NC6=CC=CC7=C6N5C8=CC=CC=C8S7(=O)=O	0.4	0.18
TerCz-IPTZ[8]	C12=CC=CC=C1N3C4=C(C=CC=C4N=C3C5=CC=C(N6C7=C(C8=C6C=CC(N9C(C=CC=C%10)=C%10C%11=C9C=CC=C%11)=C8)C=C(N%12C(C=CC=C%13)=C%13C%14=C%12C=CC=C%14)C=C7)C=C5)S2	0.4	0.24
SACR-IPTZ[8]	C12=CC=CC=C1N3C4=C(C=CC=C4N=C3C5=CC=C(N6C(C=CC=C7)=C7C8(C9=CC=CC=C9C%10=C8C=CC=C%10)C%11=C6C=CC=C%11)C=C5)S2	0.33	0.19
PTZ-IPTZ[8]	C12=CC=CC=C1N3C4=C(C=CC=C4N=C3C5=CC=C(N6C7=C(C=CC=C7)SC8=CC=CC=C86)C=C5)S2	0.06	0.13
SMAT-IPTZ[8]	CC1(C)C2=CC(C3=NC4=CC=CC5=C4N3C6=CC=CC=C6S5)=CC(C7(C)C)=C2N8C9=C(C=CC=C91)SC%10=CC=CC7=C%108	0.4	0.43
TPE[22]	C1(/C(C2=CC=CC=C2)=C(C3=CC=CC=C3)/C4=CC=CC=C4)=CC=CC=C1	1.22	0.92
TPDC[22]	C/C(C(C=C1)=CC=C1/C(C2=CC=CC=C2)=C(C3=CC=CC=C3)/C4=CC=CC=C4)=C(C#N)/C#N	0.48	0.41

TPPDC[22]	N#C/C(C#N)=C(C1=CC=CC=C1)\C(C=C2)=CC=C2/C(C3=CC=CC=C3)=C(C4=CC=CC=C4)/C5=CC=CC=C5	0.35	0.29
PPDC[22]	C/C(C(C=C1)=CC=C1C(C=C2)=CC=C2/C(C3=CC=CC=C3)=C(C4=CC=CC=C4)/C5=CC=CC=C5)=C(C#N)\C#N	0.27	0.22

Estimation of electronic character in excited state

For a set of 26 molecules, where the fraction charge transfer (CT) and local excitation (LE) character is reported, O_{FMO} was computed. Using a threshold of 50% to classify the character of a given species to either CT or LE, it is possible to correctly predict the dominant character (O_{FMO} < 0.5: CT, O_{FMO} > 0.5: LE) in 79% of the cases (88% for singlet multiplicities and 67% for triplet multiplicities) using O_{FMO} (**Table S3**).

Table S3: Benchmark set of molecules with reported electronic character and computed HOMO-LUMO overlap (O_{FMO}).

Molecule	SMILES	Multiplicity	Reported electronic character	O_{FMO}
CBP[23]	C1(N(C2=CC=C(C3=CC=C(N(C4=C5C=CC=C4)C6=C5C=CC=C6)C=C3)C=C2)C7=C8C=CC=C7)=C8C=CC=C1	Singlet	53% CT	0.53
		Triplet	76% LE	0.24
CC2TA[23]	C1(N(C2=C3C=CC=C2)C4=C3C=C(N(C5=C6C=CC=C5)C7=C6C=CC=C7)C=C4)=NC(C8=CC=CC=C8)=NC(N(C9=C%10C=CC=C9)C%11=C%10C=C(N(C%12=C%13C=CC=C%12)C%14=C%13C=CC=C%14)C=C%11)=N1	Singlet	88% CT	0.17
		Triplet	94% LE	0.06
α-NPD[23]	C1(N(C2=CC=CC=C2)C3=CC=C(C4=CC=C(N(C5=CC=CC=C5)C6=C(C=CC=C7)C7=CC=C6)C=C4)C=C3)=CC=CC8=C1C=CC=C8	Singlet	72% CT	0.51
		Triplet	71% LE	0.85
PIC-TRZ[23]	C1(N(C2=C3C=CC4=C2N(C5=CC=CC=C5)C6=C4C=CC=C6)C7=C3C=CC=C7)=NC(C8=CC=C(C9=CC=CC=C9)C=	Singlet	89% CT	0.27
		Triplet	14% LE	0.56

	C8)=NC(N(C%10=C%11C=CC%12=C%10N(C%13=CC=CC=C%13)C%14=C%12C=CC=C%14)C%15=C%11C=CC=C%15)=N1			
PXZ-TRZ[23]	C1(C2=CC=CC=C2)=NC(C3=CC=CC=C3)=NC(C4=CC=C(N5C(C=CC=C6)=C6OC7=C5C=CC=C7)C=C4)=N1	Singlet	91% CT	0.23
		Triplet	21% LE	0.05
ACRFLCN[23]	N#CC(C=C1)=CC2=C1C3=C(C24C(C=CC=C5)=C5N(C6=CC=CC=C6)C7=C4C=CC=C7)C=C(C#N)C=C3	Singlet	92% CT	0.14
		Triplet	96% LE	0.01
Spiro-CN[23]	CC(C=C1)=CC=C1N(C2=CC=C(C)C=C2)C3=CC(C4=CC=C(N(C5=CC=C(C)C=C5)C6=CC=C(C)C=C6)C=C4C78C9=CC(C#N)=CC=C9C%10=C7C=C(C#N)C=C%10)=C8C=C3	Singlet	98% CT	0.05
		Triplet	4% LE	0.97
4CzIPN[23]	N#CC1=C(N2C3=CC=CC=C3C4=CC=CC=C42)C(N5C6=CC=CC=C6C7=CC=CC=C75)=C(N8C9=CC=CC=C9C%10=CC=CC=C%108)C(C#N)=C1N%11C(C=CC=C%12)=C%12C%13=C%11C=CC=C%13	Singlet	89% CT	0.32
		Triplet	22% LE	0.61
4CzIPN-Me[23]	N#CC1=C(C(N2C3=CC=C(C)C=C3C4=CC(C)=CC=C42)=C(C(C#N)=C1N5C6=C(C7=C5C=CC(C)=C7)C=C(C)C=C6)N8C9=CC=C(C)C=C9C%10=CC(C)=CC=C%108)N%11C%12=CC=C(C)C=C%12C%13=CC(C)=CC=C%13%11	Singlet	88% CT	0.23
		Triplet	19% LE	0.60

4Cz-TPN[23]	N#CC1=C(N2C3=C(C4=C2C=CC=C4)C=CC=C3)C(N5C6=C(C=CC=C6)C7=C5C=CC=C7)=C(C#N)C(N8C9=C(C%10=C8C=CC=C%10)C=CC=C9)=C1N%11C%12=C(C%13=C%11C=CC=C%13)C=CC=C%12	Singlet<br>Triplet	88% CT<br>27% LE	0.38<br>0.41
4Cz-TPN-Me[23]	N#CC1=C(C(N2C3=C(C4=C2C=CC(C)=C4)C=C(C)C=C3)=C(C(N5C6=C(C=C(C)C=C6)C7=C5C=CC(C)=C7)=C1N8C9=C(C=C(C)C=C9)C%10=C8C=CC(C)=C%10)C#N)N%11C%12=C(C=C(C)C=C%12)C%13=C%11C=CC(C)=C%13	Singlet<br>Triplet	88% CT<br>17% LE	0.38<br>0.37
CzPN[24]	N#CC1=CC(N2C3=C(C=CC=C3)C4=C2C=CC=C4)=CC=C1C#N	Singlet<br>Triplet	92% CT<br>77% LE	0.34<br>0.74
2CzPN[24]	N#CC1=CC(N2C3=C(C=CC=C3)C4=C2C=CC=C4)=C(N5C6=C(C=CC=C6)C7=C5C=CC=C7)C=C1C#N	Singlet<br>Triplet	92% CT<br>65% LE	0.41<br>0.48
3CzPN[24]	N#CC1=CC(N2C3=C(C=CC=C3)C4=C2C=CC=C4)=C(N5C6=C(C=CC=C6)C7=C5C=CC=C7)C(N8C9=C(C=CC=C9)C%10=C8C=CC=C%10)=C1C#N	Singlet<br>Triplet	92% CT<br>59% LE	0.23<br>0.56
4CzPN[24]	N#CC1=C(N2C3=C(C=CC=C3)C4=C2C=CC=C4)C(N5C6=C(C=CC=C6)C7=C5C=CC=C7)=C(N8C9=C(C=CC=C9)C%10=C8C=CC=C%10)C(N%11C%12=C(C=CC=C%12)C%13=C%11C=CC=C%13)=C1C#N	Singlet<br>Triplet	91% CT<br>51% LE	0.25<br>0.64
TerCz-IMAC[8]	CC1(C)C(C=CC=C2N=C3C4=CC=C(C=C4)N5C6=C(C=C(C=C6)N7C8=C(C9=C7C=CC=C9)C=CC=C8)C%10=C5C=	Singlet<br>Triplet	60% CT<br>83% LE	0.24<br>0.88

	CC(N%11C%12=C(C%13=C%11C=CC=C%13)C=CC=C%12)=C%10)=C2N3C%14=CC=CC=C1%14			
TerCz-IPTZ[8]	C12=CC=CC=C1N3C4=C(C=CC=C4N=C3C5=CC=C(N6C7=C(C8=C6C=CC(N9C(C=CC=C%10)=C%10C%11=C9C=CC=C%11)=C8)C=C(N%12C(C=CC=C%13)=C%13C%14=C%12C=CC=C%14)C=C7)C=C5)S2	Singlet<br>Triplet	55% CT<br>69% LE	0.19<br>0.79
SACR-IPTZ[8]	C12=CC=CC=C1N3C4=C(C=CC=C4N=C3C5=CC=C(N6C(C=CC=C7)=C7C8(C9=CC=CC=C9C%10=C8C=CC=C%10)C%11=C6C=CC=C%11)C=C5)S2	Singlet<br>Triplet	92% CT<br>73% LE	0.15<br>0.02
ACR-IPTZ[8]	CC(C1=C2C=CC=C1)(C)C3=C(C=CC=C3)N2C(C=C4)=CC=C4C5=NC6=CC=CC7=C6N5C8=CC=CC=C8S7	Singlet<br>Triplet	93% CT<br>76% LE	0.13<br>0.60
ACR-IPTZO[8]	CC(C1=C2C=CC=C1)(C)C3=C(C=CC=C3)N2C(C=C4)=CC=C4C5=NC6=CC=CC7=C6N5C8=CC=CC=C8S7=O	Singlet<br>Triplet	93% CT<br>7% LE	0.23<br>0.49
$ACR-IPTZO_2$[8]	CC(C1=C2C=CC=C1)(C)C3=C(C=CC=C3)N2C(C=C4)=CC=C4C5=NC6=CC=CC7=C6N5C8=CC=CC=C8S7(=O)=O	Singlet<br>Triplet	94% CT<br>6% LE	0.09<br>0.09
NPh_3[25]	C1(N(C2=CC=CC=C2)C3=CC=CC=C3)=CC=CC=C1	Singlet	32% CT	0.64

PhCz[25]	C1(N(C2=CC=CC=C2)C3=C4C=CC=C3)=C4C=CC=C1	Singlet	9% CT	0.69
DPA-DPS[25]	O=S(C1=CC=C(N(C2=CC=CC=C2)C3=CC=CC=C3)C=C1)(C4=CC=C(N(C5=CC=CC=C5)C6=CC=CC=C6)C=C4)=O	Singlet	48% CT	0.62
DTPA-DPS[25]	O=S(C1=CC=C(N(C2=CC=C(C(C)(C)C)C=C2)C3=CC=C(C(C)(C)C)C=C3)C=C1)(C4=CC=C(N(C5=CC=C(C(C)(C)C)C=C5)C6=CC=C(C(C)(C)C)C=C6)C=C4)=O	Singlet	50% CT	0.54
DTC-DPS[25]	O=S(C1=CC=C(N2C3=CC=C(C(C)(C)C)C=C3C4=C2C=CC(C(C)(C)C)=C4)C=C1)(C5=CC=C(N6C7=CC=C(C(C)(C)C)C=C7C8=C6C=CC(C(C)(C)C)=C8)C=C5)=O	Singlet	76% CT	0.44

It is worth noting that the molecules in this benchmark, as well as the benchmark set for $\Delta E_{S_1-T_1}$ are mostly thermally activated delayed fluorescence (TADF) molecules. For molecules from other domains these values are much more seldomly reported which makes a comprehensive evaluation challenging.

1.2.4. Degree of conjugation

The degree of conjugation was determined as the ratio of conjugated bonds (as implemented in RDKit)[26] to total number of bonds per molecule.

1.2.5. Rigidity

To obtain a descriptor for a molecules rigidity, the $nConf_{20}$ descriptor was used as proposed by Cooper *et al.*[27] This descriptor counts the number of unique conformers (*e.g.*, RMS > 1 Å) within a 20 kcal/mol energy window to represent the conformational flexibility of a molecule.

1.2.6. SA score

The (synthetic accessibility) SA score,[28] as implemented in RDKit, was used as a scoring component. This score measures synthetic accessibility based on fragment-frequency statistics and molecular-complexity

penalties (*e.g.*, bridgehead atoms, stereocenters, or macrocycles)

1.3. Aggregation functions

To scale the properties and compute the final reward sigmoid, double sigmoid and step transformation functions were employed.

The function for the sigmoid transformation is:

$$score(x) = \frac{1}{1+e^{-k\cdot x}}, \text{ with } x = x - \frac{coef_{high}+coef_{low}}{2}, \text{ and } k = \frac{10\cdot coef_k}{coef_{high}+coef_{low}}.$$

The function for the double sigmoid function is:

$$score(x) = \begin{cases} \frac{1}{1+e^{-k_1(x-coef_{low})}} & for\ x < x_{mid} \\ 1 - \frac{1}{1+e^{-k_2(x-coef_{high})}} & for\ x \geq x_{mid} \end{cases}, \text{ with } x_{mid} = \frac{coef_{high}+coef_{low}}{2}, \text{ and } k_1 = \frac{coef_{si}}{coef_{div}}, \text{ and } k_2 = \frac{coef_{se}}{coef_{div}}$$

The left step, and right step functions are, respectively:

$$score(x) = \begin{cases} 0\ for\ x < k_{step} \\ 1\ for\ x \geq k_{step} \end{cases}, score(x) = \begin{cases} 1\ for\ x < k_{step} \\ 0\ for\ x \geq k_{step} \end{cases}$$

The transformed values are weighted, and the final reward is computed either through additive aggregation of the weighted individual scores or through multiplicative aggregation. Moreover, an aggregation function where the hypervolume improvement of the properties was used as the reward. For this, the current pareto frontier was obtained by identifying the dominant points using the properties of all previously generated molecules. The hypervolume for this pareto frontier with respect to a reference point with a value of 10^{-8} for each objective served as a reference. Then for each newly generated molecule, the hypervolume is re-calculated with the new point added to the baseline frontier. The difference between this hypervolume and the reference hypervolume served as the reward.

1.4. Production runs

1.4.1. Rediscovery

Table S4: Run parameters for the rediscovery of known organic PC.

Prior	Excluded SMARTS	Scoring component 1 (weight)	Transformation parameters	Scoring component 2 (weight)	Transformation parameters	Scoring component 3 (weight)	Transformation parameters	Aggregation function
Default	None	Triplet energy prediction (1.0)	Sigmoid function<br>$coef_{high} = 65$<br>$coef_{low} = 58$<br>$coef_k = 0.6$	ML prediction of maximum absorption wavelength (1.0)	Double sigmoid<br>$coef_{high} = 430$<br>$coef_{low} = 365$<br>$coef_{div} = 10$<br>$coef_{si} = 2.5$<br>$coef_{se} = 5$	Degree of conjugation (1.0)	Sigmoid function<br>$coef_{high} = 0.95$<br>$coef_{low} = 0.25$<br>$coef_k = 0.25$	Product

Default	None	Triplet energy prediction (1.0)	Sigmoid function<br>$coef_{high} = 65$<br>$coef_{low} = 58$<br>$coef_k = 0.6$	ML prediction of maximum absorption wavelength (1.0)	Double sigmoid<br>$coef_{high} = 430$<br>$coef_{low} = 365$<br>$coef_{div} = 10$<br>$coef_{si} = 2.5$<br>$coef_{se} = 5$	Degree of conjugation (1.0)	Sigmoid function<br>$coef_{high} = 0.95$<br>$coef_{low} = 0.25$<br>$coef_k = 0.25$	Summation

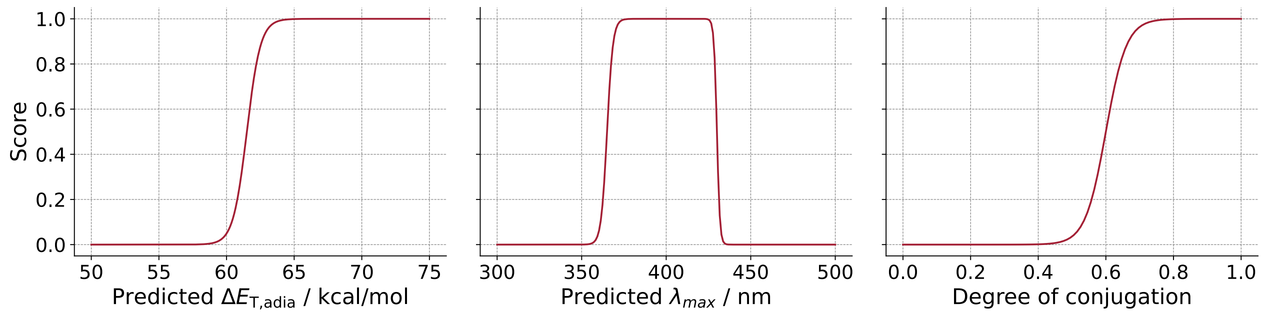

Figure S8: Transformation functions used for the rediscovery of known organic PC.

1.4.2. Chemical space exploration

Table S5: Run parameters used for the chemical space exploration.

Prior	Excluded SMARTS	Scoring component 1 (weight)	Transformation parameters	Scoring component 2 (weight)	Transformation parameters	Scoring component 3 (weight)	Transformation parameters	Aggrega-tion function
Default	[*;r3-4,r7-17]<br>[N;X3]<br>[#6]=O<br>[OH]<br>C=C<br>[!#6][!#6]<br>[CH2]	Triplet energy prediction (1.5)	Double sigmoid<br>$coef_{high} = 75$<br>$coef_{low} = 55$<br>$coef_{div} = 1$<br>$coef_{si} = 0.5$<br>$coef_{se} = 5$	ML prediction of maximum absorption wavelength (1.0)	Double sigmoid<br>$coef_{high} = 430$<br>$coef_{low} = 330$<br>$coef_{div} = 10$<br>$coef_{si} = 0.5$<br>$coef_{se} = 5$	Degree of conjugation (1.0)	Sigmoid function<br>$coef_{high} = 0.85$<br>$coef_{low} = 0.25$<br>$coef_k = 1$	Product

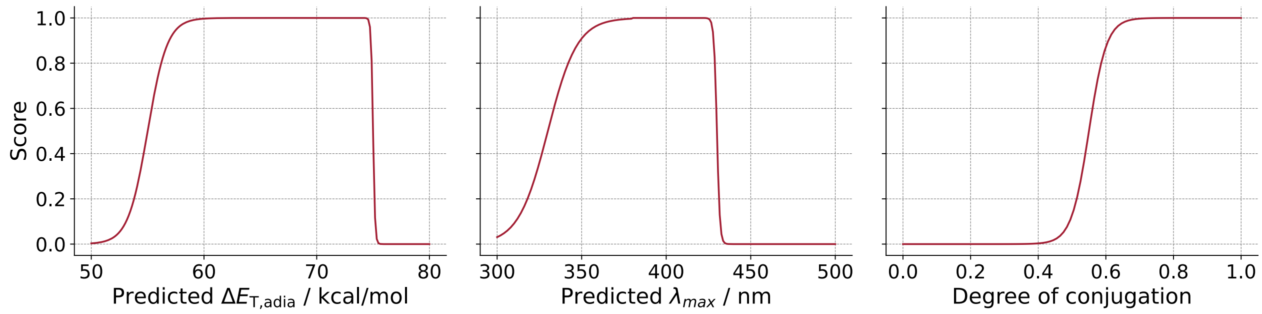

Figure S9: Transformation functions used for the chemical space exploration.

1.4.3. Evaluation of O_{FMO}

The influence of O_{FMO} on the output of the generative model was investigated by analyzing the property distributions of all molecules with a score > 0 in five runs using different random seeds. Additionally, the presence or absence of fused rings in the molecule was computed using RDKit.

Table S6: parameters for the evaluation of O_{FMO}.

Prior	Excluded SMARTS	Scoring component 1 (weight)	Transformation parameters	Scoring component 2 (weight)	Transformation parameters
Default	[*;r3-4,r7-17]<br>[N;X3]<br>[#6]=O<br>[OH]<br>C=C<br>[!#6][!#6]<br>[CH2]	Triplet energy prediction (1.5)	Double sigmoid<br>$coef_{high} = 75$<br>$coef_{low} = 55$<br>$coef_{div} = 1$<br>$coef_{si} = 0.5$<br>$coef_{se} = 5$	ML prediction of maximum absorption wavelength (1.0)	Double sigmoid<br>$coef_{high} = 430$<br>$coef_{low} = 330$<br>$coef_{div} = 10$<br>$coef_{si} = 0.5$<br>$coef_{se} = 5$
**Scoring component 3 (weight)**	**Transformation parameters**	**Scoring component 4 (weight)**	**Transformation parameters**	**Aggregation function**	
FMO analysis (1.0)	Sigmoid function<br>$coef_{high} = 0$<br>$coef_{low} = 1$<br>$coef_k = 0.4$	Degree of conjugation (1.0)	Sigmoid function<br>$coef_{high} = 0.85$<br>$coef_{low} = 0.25$<br>$coef_k = 1$	Product	

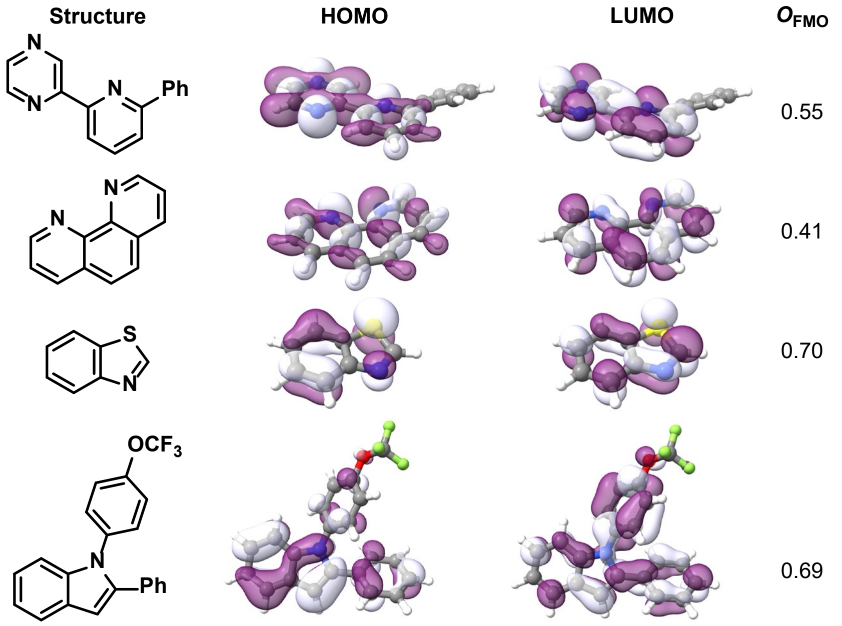

Figure S10: Structures from the run without O_{FMO}. The HOMO and LUMO of the respective molecule is computed using GFN2-xTB and Multiwfn.[12,18] Visualizations are created with ChimeraX.[29]

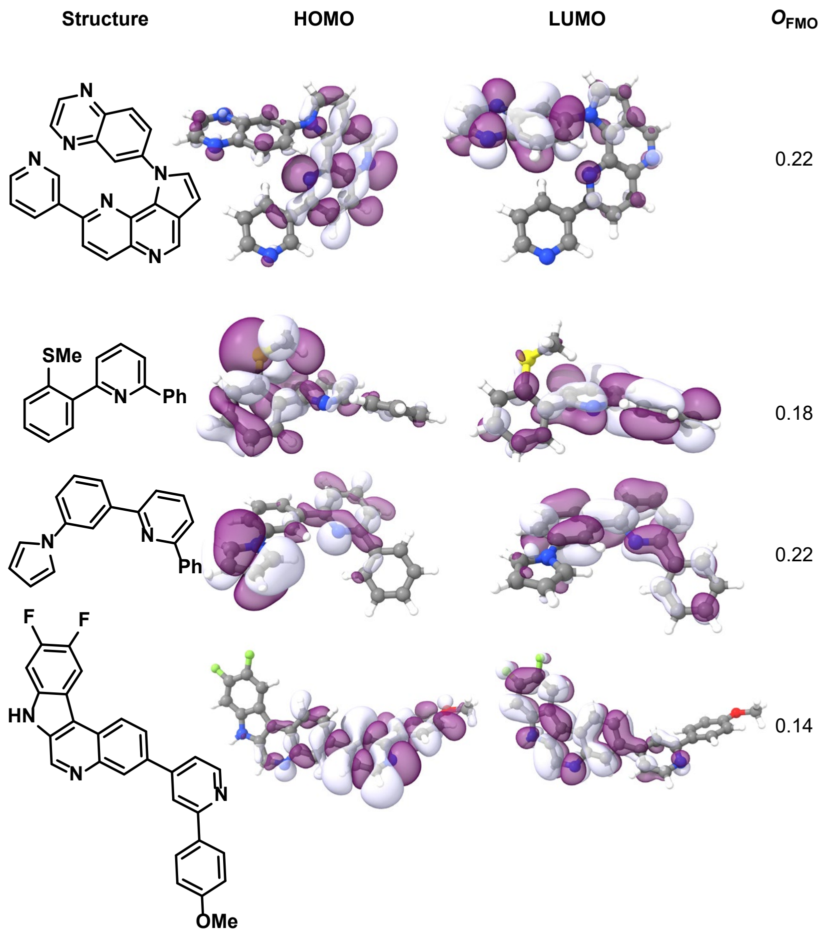

Figure S11: Structures from the run with O_{FMO}. The HOMO and LUMO of the respective molecule is computed using GFN2-xTB and Multiwfn.[12,18] Visualizations are created with ChimeraX.[29]

1.4.4. Overview of run parameters

Table S7: Overview of run parameters.

Prior	Excluded SMARTS	Scoring component 1 (weight)	Transforma-tion parameters	Scoring component 2 (weight)	Transforma-tion parameters	Scoring component 3 (weight)	Transforma-tion parameters	Scoring component 4 (weight)	Transforma-tion parameters	Aggrega-tion function
ZINC	None	Triplet energy prediction (1.0)	Sigmoid function $coef_{high} = 65$ $coef_{low} = 58$ $coef_k = 0.6$	ML prediction of maximum absorption wavelength (1.0)	Double sigmoid $coef_{high} = 430$ $coef_{low} = 365$ $coef_{div} = 10$ $coef_{si} = 2.5$ $coef_{se} = 5$	Degree of conjugation (1.0)	Sigmoid function $coef_{high} = 0.95$ $coef_{low} = 0.25$ $coef_k = 1$			Product
ZINC	None	Triplet energy prediction (1.0)	Double sigmoid $coef_{high} = 72$ $coef_{low} = 55$ $coef_{div} = 7.5$ $coef_{si} = 2.5$ $coef_{se} = 5$	ML prediction of maximum absorption wavelength (1.0)	Double sigmoid $coef_{high} = 430$ $coef_{low} = 365$ $coef_{div} = 10$ $coef_{si} = 2.5$ $coef_{se} = 5$	Degree of conjugation (1.0)	Sigmoid function $coef_{high} = 0.95$ $coef_{low} = 0.25$ $coef_k = 1$			Product
ZINC	None	Triplet energy prediction (1.0)	Sigmoid function $coef_{high} = 65$ $coef_{low} = 58$ $coef_k = 0.6$	Computational prediction of maximum absorption wavelength (1.0)	Double sigmoid $coef_{high} = 420$ $coef_{low} = 345$ $coef_{div} = 10$	Degree of conjugation (1.0)	Sigmoid function $coef_{high} = 0.95$ $coef_{low} = 0.25$ $coef_k = 1$			Product

					$coef_{si} = 2.5$<br>$coef_{se} = 5$					
ZINC	[N;X3]<br>[!#6][!#6]	Triplet energy prediction (1.0)	Double sigmoid<br>$coef_{high} = 72$<br>$coef_{low} = 55$<br>$coef_{div} = 7.5$<br>$coef_{si} = 2.5$<br>$coef_{se} = 5$	ML prediction of maximum absorption wavelength (1.0)	Double sigmoid<br>$coef_{high} = 430$<br>$coef_{low} = 365$<br>$coef_{div} = 10$<br>$coef_{si} = 2.5$<br>$coef_{se} = 5$	Degree of conjugation (1.0)	Sigmoid function<br>$coef_{high} = 0.95$<br>$coef_{low} = 0.25$<br>$coef_{k} = 1$			Product
ZINC	[N;X3]<br>[!#6][!#6]<br>[#6]=O	Triplet energy prediction (1.0)	Double sigmoid<br>$coef_{high} = 72$<br>$coef_{low} = 55$<br>$coef_{div} = 7.5$<br>$coef_{si} = 2.5$<br>$coef_{se} = 5$	ML prediction of maximum absorption wavelength (1.0)	Double sigmoid<br>$coef_{high} = 430$<br>$coef_{low} = 365$<br>$coef_{div} = 10$<br>$coef_{si} = 2.5$<br>$coef_{se} = 5$	Degree of conjugation (1.0)	Sigmoid function<br>$coef_{high} = 0.95$<br>$coef_{low} = 0.25$<br>$coef_{k} = 1$			Product

ZINC	[*;r3-4,r7-17]<br>[N;X3]<br>[#6]=O<br>[OH]<br>C=C<br>[!#6][!#6]<br>[CH2]	Triplet energy prediction (3.0)	Double sigmoid<br>$coef_{high} = 72$<br>$coef_{low} = 55$<br>$coef_{div} = 7.5$<br>$coef_{si} = 2.5$<br>$coef_{se} = 5$	Computational prediction of maximum absorption wavelength (2.0)	Sigmoid function<br>$coef_{high} = 400$<br>$coef_{low} = 300$<br>$coef_{k} = 0.1$	Degree of conjugation (1.0)	Sigmoid function<br>$coef_{high} = 0.85$<br>$coef_{low} = 0.25$<br>$coef_{k} = 1$			Product
ZINC	[*;r3-4,r7-17]<br>[N;X3]<br>[#6]=O<br>[OH]<br>C=C<br>[!#6][!#6]<br>[CH2]<br>[#17]<br>[#35]<br>[#53]	Triplet energy prediction (3.0)	Double sigmoid<br>$coef_{high} = 75$<br>$coef_{low} = 55$<br>$coef_{div} = 1$<br>$coef_{si} = 0.5$<br>$coef_{se} = 5$	Computational prediction of maximum absorption wavelength (2.0)	Sigmoid function<br>$coef_{high} = 400$<br>$coef_{low} = 300$<br>$coef_{k} = 0.1$	Degree of conjugation (1.0)	Sigmoid function<br>$coef_{high} = 0.85$<br>$coef_{low} = 0.25$<br>$coef_{k} = 1$			Product
ZINC	[*;r3-4,r7-17]<br>[N;X3]<br>[#6]=O<br>[OH]<br>C=C<br>[!#6][!#6]<br>[CH2]<br>[#17]<br>[#35]<br>[#53]	Triplet energy prediction (3.0)	Double sigmoid<br>$coef_{high} = 75$<br>$coef_{low} = 55$<br>$coef_{div} = 1$<br>$coef_{si} = 0.5$<br>$coef_{se} = 5$	Computational prediction of maximum absorption wavelength (2.0)	Sigmoid function<br>$coef_{high} = 400$<br>$coef_{low} = 300$<br>$coef_{k} = 0.1$	Degree of conjugation (1.0)	Sigmoid function<br>$coef_{high} = 0.85$<br>$coef_{low} = 0.25$<br>$coef_{k} = 1$	FMO analysis (1.0)	Right step function<br>$k_{step} = 0.5$	Product

ZINC	[*;r3-4,r7-17]<br>[N;X3]<br>[#6]=O<br>C=C<br>[!#6][!#6]<br>[CH2]	Triplet energy prediction (3.0)	Double sigmoid<br>$coef_{high} = 75$<br>$coef_{low} = 55$<br>$coef_{div} = 1$<br>$coef_{si} = 0.5$<br>$coef_{se} = 5$	ML prediction of maximum absorption wavelength (1.0)	Double sigmoid<br>$coef_{high} = 430$<br>$coef_{low} = 330$<br>$coef_{div} = 10$<br>$coef_{si} = 0.5$<br>$coef_{se} = 5$	Degree of conjugation (1.0)	Sigmoid function<br>$coef_{high} = 0.85$<br>$coef_{low} = 0.25$<br>$coef_{k} = 1$			Product
TADF	None	Triplet energy prediction (2.5)	Double sigmoid<br>$coef_{high} = 77$<br>$coef_{low} = 63$<br>$coef_{div} = 5$<br>$coef_{si} = 2$<br>$coef_{se} = 2$	ML prediction of maximum absorption wavelength (2.5)	Double sigmoid<br>$coef_{high} = 430$<br>$coef_{low} = 365$<br>$coef_{div} = 10$<br>$coef_{si} = 1$<br>$coef_{se} = 5$	Rigidity (1.0)	Sigmoid function<br>$coef_{high} = -1$<br>$coef_{low} = 8$<br>$coef_{k} = 0.5$	FMO analysis (2.5)	Sigmoid function<br>$coef_{high} = 0$<br>$coef_{low} = 1$<br>$coef_{k} = 1.5$	Product + Hypervolume
TADF	None	Triplet energy prediction (2.5)	Double sigmoid<br>$coef_{high} = 77$<br>$coef_{low} = 63$<br>$coef_{div} = 5$<br>$coef_{si} = 2$<br>$coef_{se} = 2$	ML prediction of maximum absorption wavelength (2.5)	Double sigmoid<br>$coef_{high} = 430$<br>$coef_{low} = 365$<br>$coef_{div} = 10$<br>$coef_{si} = 1$<br>$coef_{se} = 5$	FMO analysis (2.5)	Sigmoid function<br>$coef_{high} = 0$<br>$coef_{low} = 1$<br>$coef_{k} = 1.5$			Product + Hypervolume

TADF	[*;r3-4,r7-17]<br>[N;X3]<br>[!#6][!#6]<br>[CH2]	Triplet energy prediction (2.5)	Double sigmoid<br>$coef_{high} = 77$<br>$coef_{low} = 63$<br>$coef_{div} = 5$<br>$coef_{si} = 2$<br>$coef_{se} = 2$	ML prediction of maximum absorption wavelength (2.5)	Double sigmoid<br>$coef_{high} = 430$<br>$coef_{low} = 365$<br>$coef_{div} = 10$<br>$coef_{si} = 1$<br>$coef_{se} = 5$					Sum
TADF	[*;r3-4,r7-17]<br>[N;X3]<br>[!#6][!#6]<br>[CH2]	Triplet energy prediction (2.5)	Double sigmoid<br>$coef_{high} = 77$<br>$coef_{low} = 63$<br>$coef_{div} = 5$<br>$coef_{si} = 2$<br>$coef_{se} = 2$	ML prediction of maximum absorption wavelength (2.5)	Double sigmoid<br>$coef_{high} = 430$<br>$coef_{low} = 365$<br>$coef_{div} = 10$<br>$coef_{si} = 1$<br>$coef_{se} = 5$	FMO analysis (2.5)	Sigmoid function<br>$coef_{high} = 0$<br>$coef_{low} = 1$<br>$coef_{k} = 1$			Sum
TADF	[*;r3-4,r7-17]<br>[N;X3]<br>[!#6][!#6]	Triplet energy prediction (2.5)	Double sigmoid<br>$coef_{high} = 71$<br>$coef_{low} = 63$<br>$coef_{div} = 0.5$<br>$coef_{si} = 5$<br>$coef_{se} = 5$	ML prediction of maximum absorption wavelength (2.5)	Double sigmoid<br>$coef_{high} = 430$<br>$coef_{low} = 300$<br>$coef_{div} = 10$<br>$coef_{si} = 5$<br>$coef_{se} = 5$	Rigidity (1.0)	Sigmoid function<br>$coef_{high} = -1$<br>$coef_{low} = 8$<br>$coef_{k} = 0.5$	FMO analysis (2.5)	Sigmoid function<br>$coef_{high} = 0$<br>$coef_{low} = 1$<br>$coef_{k} = 1.5$	Sum

TADF	[*;r3-4,r7-17]<br>[N;X3]<br>[#6]=O<br>[!#6][!#6]	Triplet energy prediction (2.0)	Double sigmoid<br>$coef_{high} = 77$<br>$coef_{low} = 63$<br>$coef_{div} = 5$<br>$coef_{si} = 2$<br>$coef_{se} = 2$	ML prediction of maximum absorption wavelength (1)	Double sigmoid<br>$coef_{high} = 430$<br>$coef_{low} = 365$<br>$coef_{div} = 10$<br>$coef_{si} = 1$<br>$coef_{se} = 5$	Degree of conjugation (1.0)	Sigmoid function<br>$coef_{high} = 0.95$<br>$coef_{low} = 0.25$<br>$coef_{k} = 1$	FMO analysis (1.0)	Sigmoid function<br>$coef_{high} = 0$<br>$coef_{low} = 1$<br>$coef_{k} = 1.5$	Sum
TADF	[*;r3-4,r7-17]<br>[N;X3]<br>[!#6][!#6]	Triplet energy prediction (2.5)	Double sigmoid<br>$coef_{high} = 71$<br>$coef_{low} = 63$<br>$coef_{div} = 0.5$<br>$coef_{si} = 5$<br>$coef_{se} = 5$	ML prediction of maximum absorption wavelength (2.5)	Double sigmoid<br>$coef_{high} = 430$<br>$coef_{low} = 300$<br>$coef_{div} = 10$<br>$coef_{si} = 5$<br>$coef_{se} = 5$	Degree of conjugation (2.5)	Sigmoid function<br>$coef_{high} = 1$<br>$coef_{low} = 0$<br>$coef_{k} = 1$			Sum
TADF	None	Triplet energy prediction (2.5)	Double sigmoid<br>$coef_{high} = 77$<br>$coef_{low} = 63$<br>$coef_{div} = 5$<br>$coef_{si} = 2$<br>$coef_{se} = 2$	ML prediction of maximum absorption wavelength (2.5)	Double sigmoid<br>$coef_{high} = 430$<br>$coef_{low} = 365$<br>$coef_{div} = 10$<br>$coef_{si} = 1$<br>$coef_{se} = 5$					Sum

TADF	[*;r3-4,r7-17] [N;X3] [#6]=O C=C [!#6][!#6] [CH2] [OH]	Triplet energy prediction (2.5)	Double sigmoid $coef_{high} = 71$ $coef_{low} = 63$ $coef_{div} = 0.5$ $coef_{si} = 5$ $coef_{se} = 5$	ML prediction of maximum absorption wavelength (2.5)	Double sigmoid $coef_{high} = 430$ $coef_{low} = 300$ $coef_{div} = 10$ $coef_{si} = 5$ $coef_{se} = 5$	Degree of conjugation (1)	Sigmoid function $coef_{high} = 0.95$ $coef_{low} = 0.25$ $coef_{k} = 1$	FMO analysis (1.0)	Right step function $k_{step} = 0.5$	Hypervolume
TADF	[*;r3-4,r7-17] [N;X3] [#6]=O C=C [!#6][!#6] [CH2] [OH]	Triplet energy prediction (1.0)	Double sigmoid $coef_{high} = 71$ $coef_{low} = 63$ $coef_{div} = 0.5$ $coef_{si} = 5$ $coef_{se} = 5$	Computational prediction of maximum absorption wavelength (1.0)	Sigmoid function $coef_{high} = 400$ $coef_{low} = 300$ $coef_{k} = 1$	Degree of conjugation (1.0)	Sigmoid function $coef_{high} = 0.95$ $coef_{low} = 0.25$ $coef_{k} = 1$	FMO analysis (1.0)	Right step function $k_{step} = 0.5$	Hypervolume
TADF	[*;r3-4,r7-17] [N;X3] [!#6][!#6]	Triplet energy prediction (1.0)	Double sigmoid $coef_{high} = 68$ $coef_{low} = 63$ $coef_{div} = 1.5$ $coef_{si} = 2$ $coef_{se} = 2$	ML prediction of maximum absorption wavelength (2.5)	Double sigmoid $coef_{high} = 430$ $coef_{low} = 360$ $coef_{div} = 10$ $coef_{si} = 5$ $coef_{se} = 5$					Sum

TADF	[*;r3-4,r7-17]<br>[N;X3]<br>[#6]=O<br>C=C<br>[!#6][!#6]<br>[CH2]<br>[OH]	Triplet energy prediction (2.0)	Double sigmoid<br>$coef_{high} = 71$<br>$coef_{low} = 63$<br>$coef_{div} = 0.5$<br>$coef_{si} = 5$<br>$coef_{se} = 5$	ML prediction of maximum absorption wavelength (2.5)	Double sigmoid<br>$coef_{high} = 430$<br>$coef_{low} = 300$<br>$coef_{div} = 10$<br>$coef_{si} = 5$<br>$coef_{se} = 5$	Degree of conjugation (1)	Sigmoid function<br>$coef_{high} = 0.95$<br>$coef_{low} = 0.25$<br>$coef_{k} = 1$	FMO analysis (1)	Right step function<br>$k_{step} = 0.5$	Product
TADF	[*;r3-4,r7-17]<br>[N;X3]<br>[#6]=O<br>C=C<br>[!#6][!#6]<br>[CH2]<br>[OH]	Triplet energy prediction (2.0)	Double sigmoid<br>$coef_{high} = 71$<br>$coef_{low} = 63$<br>$coef_{div} = 0.5$<br>$coef_{si} = 5$<br>$coef_{se} = 5$	Computational prediction of maximum absorption wavelength (1.0)	Sigmoid function<br>$coef_{high} = 400$<br>$coef_{low} = 300$<br>$coef_{k} = 1$	Degree of conjugation (1)	Sigmoid function<br>$coef_{high} = 0.95$<br>$coef_{low} = 0.25$<br>$coef_{k} = 1$	FMO analysis (1)	Right step function<br>$k_{step} = 0.5$	Product
TADF	[*;r3-4,r7-17]<br>[N;X3]<br>[#6]=O<br>C=C<br>[!#6][!#6]<br>[CH2]	Triplet energy prediction (2.5)	Double sigmoid<br>$coef_{high} = 77$<br>$coef_{low} = 63$<br>$coef_{div} = 5$<br>$coef_{si} = 2$<br>$coef_{se} = 2$	ML prediction of maximum absorption wavelength (2.5)	Double sigmoid<br>$coef_{high} = 430$<br>$coef_{low} = 365$<br>$coef_{div} = 10$<br>$coef_{si} = 1$<br>$coef_{se} = 5$					Sum

TADF	[*;r3-4,r7-17]<br>[N;X3]<br>[#6]=O<br>C=C<br>[!#6][!#6]<br>[CH2]	Triplet energy prediction (2.5)	Double sigmoid<br>$coef_{high} = 77$<br>$coef_{low} = 63$<br>$coef_{div} = 5$<br>$coef_{si} = 2$<br>$coef_{se} = 2$	ML prediction of maximum absorption wavelength (2.5)	Double sigmoid<br>$coef_{high} = 430$<br>$coef_{low} = 365$<br>$coef_{div} = 10$<br>$coef_{si} = 1$<br>$coef_{se} = 5$	FMO analysis (2.5)	Sigmoid function<br>$coef_{high} = 0$<br>$coef_{low} = 1$<br>$coef_{k} = 1.5$			Sum
TADF	[*;r3-4,r7-17]<br>[N;X3]<br>[!#6][!#6]	Triplet energy prediction (2.5)	Double sigmoid<br>$coef_{high} = 71$<br>$coef_{low} = 63$<br>$coef_{div} = 0.5$<br>$coef_{si} = 5$<br>$coef_{se} = 5$	Computational prediction of maximum absorption wavelength (2.5)	Sigmoid function<br>$coef_{high} = 400$<br>$coef_{low} = 300$<br>$coef_{k} = 1$	Degree of conjugation (1.0)	Sigmoid function<br>$coef_{high} = 0.85$<br>$coef_{low} = 0.25$<br>$coef_{k} = 1$			Sum
Default	[*;r3-4,r7-17]<br>[N;X3]<br>[#6]=O<br>C=C<br>[!#6][!#6]<br>[CH2]	Triplet energy prediction (2.5)	Double sigmoid<br>$coef_{high} = 77$<br>$coef_{low} = 63$<br>$coef_{div} = 5$<br>$coef_{si} = 2$<br>$coef_{se} = 2$	Computational prediction of maximum absorption wavelength (1.0)	Sigmoid function<br>$coef_{high} = 400$<br>$coef_{low} = 300$<br>$coef_{k} = 1$	Degree of conjugation (1.0)	Sigmoid function<br>$coef_{high} = 0.85$<br>$coef_{low} = 0.25$<br>$coef_{k} = 1$	FMO analysis (1.0)	Right step function<br>$k_{step} = 0.5$	Sum

Default	[*;r3-4,r7-17] [N;X3] [#6]=O C=C [!#6][!#6] [CH2]	Triplet energy prediction (2.5)	Double sigmoid $coef_{high} = 77$ $coef_{low} = 63$ $coef_{div} = 5$ $coef_{si} = 2$ $coef_{se} = 2$	Computational prediction of maximum absorption wavelength (1.0)	Sigmoid function $coef_{high} = 400$ $coef_{low} = 300$ $coef_{k} = 1$	Degree of conjugation (1.0)	Sigmoid function $coef_{high} = 0.85$ $coef_{low} = 0.25$ $coef_{k} = 1$	FMO analysis (CT/LE estimation) (1.0)	Right step function (S1) $k_{step} = 0.4$ + Left step function (S1) $k_{step} = 0.6$	Sum
Default	[*;r3-4,r7-17] [N;X3] [#6]=O C=C [!#6][!#6] [CH2]	Triplet energy prediction (1.0)	Double sigmoid $coef_{high} = 77$ $coef_{low} = 63$ $coef_{div} = 5$ $coef_{si} = 2$ $coef_{se} = 2$	ML prediction of maximum absorption wavelength (1.0)	Double sigmoid $coef_{high} = 430$ $coef_{low} = 365$ $coef_{div} = 10$ $coef_{si} = 1$ $coef_{se} = 5$	Degree of conjugation (1.0)	Sigmoid function $coef_{high} = 0.85$ $coef_{low} = 0.25$ $coef_{k} = 1$	FMO analysis (CT/LE estimation) (2.5)	Right step function (S1) $k_{step} = 0.4$ + Left step function (S1) $k_{step} = 0.6$	Sum
Default	[*;r3-4,r7-17] [N;X3] [#6]=O C=C [!#6][!#6] [CH2] [r](=N)	Triplet energy prediction (2.0)	Double sigmoid $coef_{high} = 77$ $coef_{low} = 63$ $coef_{div} = 5$ $coef_{si} = 2$ $coef_{se} = 2$	ML prediction of maximum absorption wavelength (1.0)	Double sigmoid $coef_{high} = 430$ $coef_{low} = 365$ $coef_{div} = 10$ $coef_{si} = 1$ $coef_{se} = 5$	Degree of conjugation (1.0)	Sigmoid function $coef_{high} = 0.85$ $coef_{low} = 0.25$ $coef_{k} = 1$	FMO analysis (1.0)	Right step function $k_{step} = 0.5$	Product

Default	[*;r3-4,r7-17]<br>[N;X3]<br>[#6]=O<br>C=C<br>[!#6][!#6]<br>[CH2]	Triplet energy prediction (1.0)	Double sigmoid<br>$coef_{high} = 77$<br>$coef_{low} = 63$<br>$coef_{div} = 5$<br>$coef_{si} = 2$<br>$coef_{se} = 2$	ML prediction of maximum absorption wavelength (1.0)	Double sigmoid<br>$coef_{high} = 430$<br>$coef_{low} = 365$<br>$coef_{div} = 10$<br>$coef_{si} = 1$<br>$coef_{se} = 5$	Degree of conjugation (1.0)	Sigmoid function<br>$coef_{high} = 0.85$<br>$coef_{low} = 0.25$<br>$coef_{k} = 1$	FMO analysis (2.5)	Right step function<br>$k_{step} = 0.5$	Sum
Default	[*;r3-4,r7-17]<br>[N;X3]<br>[#6]=O<br>C=C<br>[!#6][!#6]<br>[CH2]	Triplet energy prediction (1.0)	Double sigmoid<br>$coef_{high} = 77$<br>$coef_{low} = 63$<br>$coef_{div} = 5$<br>$coef_{si} = 2$<br>$coef_{se} = 2$	Computational prediction of maximum absorption wavelength (2.5)	Sigmoid function<br>$coef_{high} = 400$<br>$coef_{low} = 300$<br>$coef_{k} = 1$	Degree of conjugation (1.0)	Sigmoid function<br>$coef_{high} = 0.85$<br>$coef_{low} = 0.25$<br>$coef_{k} = 1$	FMO analysis (2.5)	Right step function<br>$k_{step} = 0.5$	Sum
Default	[*;r3-4,r7-17]<br>[N;X3]<br>[#6]=O<br>C=C<br>[!#6][!#6]<br>[CH2]	Triplet energy prediction (1.0)	Double sigmoid<br>$coef_{high} = 77$<br>$coef_{low} = 63$<br>$coef_{div} = 5$<br>$coef_{si} = 2$<br>$coef_{se} = 2$	Computational prediction of maximum absorption wavelength (2.5)	Sigmoid function<br>$coef_{high} = 400$<br>$coef_{low} = 300$<br>$coef_{k} = 1$	Degree of conjugation (1.0)	Sigmoid function<br>$coef_{high} = 0.85$<br>$coef_{low} = 0.25$<br>$coef_{k} = 1$	FMO analysis (CT/LE estimation) (2.5)	Right step function (S1)<br>$k_{step} = 0.4$<br>+<br>Left step function (S1)<br>$k_{step} = 0.6$	Sum

Default	[*;r3-4,r7-17]<br>[N;X3]<br>[#6]=O<br>C=C<br>[!#6][!#6]<br>[CH2]	Triplet energy prediction (1.0)	Double sigmoid<br>$coef_{high} = 77$<br>$coef_{low} = 63$<br>$coef_{div} = 5$<br>$coef_{si} = 2$<br>$coef_{se} = 2$	ML prediction of maximum absorption wavelength (1.0)	Double sigmoid<br>$coef_{high} = 430$<br>$coef_{low} = 365$<br>$coef_{div} = 10$<br>$coef_{si} = 1$<br>$coef_{se} = 5$	Degree of conjugation (1.0)	Sigmoid function<br>$coef_{high} = 0.85$<br>$coef_{low} = 0.25$<br>$coef_{k} = 1$	FMO analysis (1.0)	Right step function<br>$k_{step} = 0.5$	Sum
Default	[*;r3-4,r7-17]<br>[N;X3]<br>[!#6][!#6]<br>[CH2]	Triplet energy prediction (2.5)	Double sigmoid<br>$coef_{high} = 77$<br>$coef_{low} = 63$<br>$coef_{div} = 5$<br>$coef_{si} = 2$<br>$coef_{se} = 2$	ML prediction of maximum absorption wavelength (2.5)	Double sigmoid<br>$coef_{high} = 430$<br>$coef_{low} = 365$<br>$coef_{div} = 10$<br>$coef_{si} = 1$<br>$coef_{se} = 5$	FMO analysis (2.5)	Sigmoid function<br>$coef_{high} = 0$<br>$coef_{low} = 1$<br>$coef_{k} = 1$	Rigidity (1.0)	Sigmoid function<br>$coef_{high} = -1$<br>$coef_{low} = 8$<br>$coef_{k} = 0.5$	Product + Hypervolume
Default	[*;r3-4,r7-17]<br>[N;X3]<br>[!#6][!#6]<br>[CH2]	Triplet energy prediction (1.0)	Sigmoid function<br>$coef_{high} = 70$<br>$coef_{low} = 55$<br>$coef_{k} = 0.6$	ML prediction of maximum absorption wavelength (2.0)	Double sigmoid<br>$coef_{high} = 420$<br>$coef_{low} = 360$<br>$coef_{div} = 25$<br>$coef_{si} = 1$<br>$coef_{se} = 5$					Product

Default	[*;r3-4,r7-17] [N;X3] [!#6][!#6] [CH2] [#6]=O	Triplet energy prediction (6.0)	Double sigmoid $coef_{high} = 77$ $coef_{low} = 63$ $coef_{div} = 5$ $coef_{si} = 2$ $coef_{se} = 2$	ML prediction of maximum absorption wavelength (2.5)	Double sigmoid $coef_{high} = 430$ $coef_{low} = 360$ $coef_{div} = 25$ $coef_{si} = 1$ $coef_{se} = 5$	FMO analysis (2.5)	Sigmoid function $coef_{high} = 0$ $coef_{low} = 1$ $coef_{k} = 1$	Degree of conjugation (2.5)	Sigmoid function $coef_{high} = 0.85$ $coef_{low} = 0.25$ $coef_{k} = 1$	Sum
Default	[*;r3-4,r7-17] [N;X3] [!#6][!#6] [CH2]	Triplet energy prediction (2.5)	Double sigmoid $coef_{high} = 77$ $coef_{low} = 63$ $coef_{div} = 5$ $coef_{si} = 2$ $coef_{se} = 2$	ML prediction of maximum absorption wavelength (2.5)	Double sigmoid $coef_{high} = 430$ $coef_{low} = 365$ $coef_{div} = 10$ $coef_{si} = 1$ $coef_{se} = 5$	SA score (1.0)	Sigmoid function $coef_{high} = 1$ $coef_{low} = 10$ $coef_{k} = 0.5$			Sum
Default	[*;r3-4,r7-17] [N;X3] [!#6][!#6] [CH2] [#6]=O C=C	Triplet energy prediction (6.0)	Double sigmoid $coef_{high} = 77$ $coef_{low} = 63$ $coef_{div} = 5$ $coef_{si} = 2$ $coef_{se} = 2$	Computational prediction of maximum absorption wavelength (2.5)	Sigmoid function $coef_{high} = 400$ $coef_{low} = 300$ $coef_{k} = 1$	FMO analysis (2.5)	Sigmoid function $coef_{high} = 0$ $coef_{low} = 1$ $coef_{k} = 1$	Degree of conjugation (2.5)	Sigmoid function $coef_{high} = 0.85$ $coef_{low} = 0.25$ $coef_{k} = 1$	Sum

Default	[*;r3-4,r7-17] [N;X3] [!#6][!#6] [CH2] [#6]=O C=C	Triplet energy prediction (6.0)	Double sigmoid $coef_{high} = 77$ $coef_{low} = 63$ $coef_{div} = 5$ $coef_{si} = 2$ $coef_{se} = 2$	ML prediction of maximum absorption wavelength (2.5)	Double sigmoid $coef_{high} = 430$ $coef_{low} = 365$ $coef_{div} = 10$ $coef_{si} = 1$ $coef_{se} = 5$	Degree of conjugation (2.5)	Sigmoid function $coef_{high} = 0.85$ $coef_{low} = 0.25$ $coef_{k} = 1$			Sum
Default	None	Triplet energy prediction (2.5)	Double sigmoid $coef_{high} = 77$ $coef_{low} = 63$ $coef_{div} = 5$ $coef_{si} = 2$ $coef_{se} = 2$	ML prediction of maximum absorption wavelength (2.5)	Double sigmoid $coef_{high} = 430$ $coef_{low} = 365$ $coef_{div} = 10$ $coef_{si} = 1$ $coef_{se} = 5$	Rigidity (1.0)	Sigmoid function $coef_{high} = -1$ $coef_{low} = 8$ $coef_{k} = 0.5$	SA score (1.0)	Sigmoid function $coef_{high} = 1$ $coef_{low} = 10$ $coef_{k} = 0.5$	Product
Default	None	Triplet energy prediction (2.5)	Double sigmoid $coef_{high} = 77$ $coef_{low} = 63$ $coef_{div} = 5$ $coef_{si} = 2$ $coef_{se} = 2$	ML prediction of maximum absorption wavelength (2.5)	Double sigmoid $coef_{high} = 430$ $coef_{low} = 365$ $coef_{div} = 10$ $coef_{si} = 1$ $coef_{se} = 5$	Rigidity (1.0)	Sigmoid function $coef_{high} = -1$ $coef_{low} = 8$ $coef_{k} = 0.5$	SA score (1.0)	Sigmoid function $coef_{high} = 1$ $coef_{low} = 10$ $coef_{k} = 0.5$	Product + Hypervolume

Default	None	Triplet energy prediction (2.5)	Double sigmoid $coef_{high} = 77$ $coef_{low} = 63$ $coef_{div} = 5$ $coef_{si} = 2$ $coef_{se} = 2$	ML prediction of maximum absorption wavelength (2.5)	Double sigmoid $coef_{high} = 430$ $coef_{low} = 365$ $coef_{div} = 10$ $coef_{si} = 1$ $coef_{se} = 5$	Rigidity (1.0)	Sigmoid function $coef_{high} = -1$ $coef_{low} = 8$ $coef_{k} = 0.5$	SA score (1.0)	Sigmoid function $coef_{high} = 1$ $coef_{low} = 10$ $coef_{k} = 0.5$	Sum
Default	None	Triplet energy prediction (2.5)	Double sigmoid $coef_{high} = 77$ $coef_{low} = 63$ $coef_{div} = 5$ $coef_{si} = 2$ $coef_{se} = 2$	ML prediction of maximum absorption wavelength (2.5)	Double sigmoid $coef_{high} = 430$ $coef_{low} = 365$ $coef_{div} = 10$ $coef_{si} = 1$ $coef_{se} = 5$	Rigidity (1.0)	Sigmoid function $coef_{high} = -1$ $coef_{low} = 8$ $coef_{k} = 0.5$	SA score (1.0)	Sigmoid function $coef_{high} = 1$ $coef_{low} = 10$ $coef_{k} = 0.5$	Hypervolume
Default	[*;r3-4,r7-17] [N;X3] [!#6][!#6] [CH2] [#6]=O	Triplet energy prediction (1.0)	Double sigmoid $coef_{high} = 77$ $coef_{low} = 63$ $coef_{div} = 5$ $coef_{si} = 2$ $coef_{se} = 2$	ML prediction of maximum absorption wavelength (1.0)	Double sigmoid $coef_{high} = 430$ $coef_{low} = 365$ $coef_{div} = 10$ $coef_{si} = 1$ $coef_{se} = 5$	FMO analysis (1.0)	Sigmoid function $coef_{high} = 0$ $coef_{low} = 1.25$ $coef_{k} = 2.5$			Product + Hypervolume

Default	[*;r3-4,r7-17] [N;X3] [!#6][!#6] [CH2] [#6]=O	Triplet energy prediction (1.0)	Double sigmoid $coef_{high} = 77$ $coef_{low} = 63$ $coef_{div} = 5$ $coef_{si} = 2$ $coef_{se} = 2$	Computational prediction of maximum absorption wavelength (1.0)	Sigmoid function $coef_{high} = 400$ $coef_{low} = 300$ $coef_{k} = 1$	Degree of conjugation (1.0)	Sigmoid function $coef_{high} = 0.95$ $coef_{low} = 0.25$ $coef_{k} = 1$			Sum
Default	[*;r3-4,r7-17] [N;X3] [!#6][!#6] [CH2]	Triplet energy prediction (1.0)	Double sigmoid $coef_{high} = 77$ $coef_{low} = 63$ $coef_{div} = 5$ $coef_{si} = 2$ $coef_{se} = 2$	Computational prediction of maximum absorption wavelength (1.0)	Sigmoid function $coef_{high} = 400$ $coef_{low} = 300$ $coef_{k} = 1$	Degree of conjugation (1.0)	Sigmoid function $coef_{high} = 0.95$ $coef_{low} = 0.25$ $coef_{k} = 1$			Sum
Default	[*;r3-4,r7-17] [N;X3] [!#6][!#6] [CH2]	Triplet energy prediction (1.0)	Double sigmoid $coef_{high} = 77$ $coef_{low} = 63$ $coef_{div} = 5$ $coef_{si} = 2$ $coef_{se} = 2$	Computational prediction of maximum absorption wavelength (1.0)	Sigmoid function $coef_{high} = 400$ $coef_{low} = 300$ $coef_{k} = 1$	Degree of conjugation (1.0)	Sigmoid function $coef_{high} = 0.95$ $coef_{low} = 0.25$ $coef_{k} = 1$	FMO analysis (1.0)	Sigmoid function $coef_{high} = 0$ $coef_{low} = 1.25$ $coef_{k} = 2.5$	Sum
Default	[*;r3-4,r7-17] [N;X3] [!#6][!#6] [CH2] [#6]=O	Triplet energy prediction (0.5)	Double sigmoid $coef_{high} = 75$ $coef_{low} = 55$ $coef_{div} = 1$	ML prediction of maximum absorption wavelength (3.0)	Double sigmoid $coef_{high} = 430$ $coef_{low} = 330$ $coef_{div} = 10$	Degree of conjugation (1.0)	Sigmoid function $coef_{high} = 0.85$ $coef_{low} = 0.25$ $coef_{k} = 1$			Product

	C=C<br>[OH]		$coef_{si} = 0.5$<br>$coef_{se} = 5$		$coef_{si} = 0.5$<br>$coef_{se} = 5$					
Default	[#6]=O	Triplet energy prediction (1.5)	Double sigmoid<br>$coef_{high} = 75$<br>$coef_{low} = 55$<br>$coef_{div} = 1$<br>$coef_{si} = 0.5$<br>$coef_{se} = 5$	ML prediction of maximum absorption wavelength (1.0)	Double sigmoid<br>$coef_{high} = 430$<br>$coef_{low} = 330$<br>$coef_{div} = 10$<br>$coef_{si} = 0.5$<br>$coef_{se} = 5$					Product
Default	[*;r3-4,r7-17]<br>[N;X3]<br>[!#6][!#6]<br>[CH2]<br>[#6]=O<br>C=C<br>[OH]	Triplet energy prediction (1.5)	Double sigmoid<br>$coef_{high} = 75$<br>$coef_{low} = 55$<br>$coef_{div} = 1$<br>$coef_{si} = 0.5$<br>$coef_{se} = 5$	ML prediction of maximum absorption wavelength (1.0)	Double sigmoid<br>$coef_{high} = 430$<br>$coef_{low} = 330$<br>$coef_{div} = 10$<br>$coef_{si} = 0.5$<br>$coef_{se} = 5$	FMO analysis (1.0)	Sigmoid function<br>$coef_{high} = 0$<br>$coef_{low} = 1$<br>$coef_{k} = 0.4$	Degree of conjugation (1.0)	Sigmoid function<br>$coef_{high} = 0.85$<br>$coef_{low} = 0.25$<br>$coef_{k} = 1$	Product

Default	[*;r3-4,r7-17] [N;X3] [!#6][!#6] [CH2] [#6]=O	Triplet energy prediction (1.0)	Double sigmoid $coef_{high} = 77$ $coef_{low} = 63$ $coef_{div} = 5$ $coef_{si} = 2$ $coef_{se} = 2$	ML prediction of maximum absorption wavelength (1.0)	Double sigmoid $coef_{high} = 430$ $coef_{low} = 365$ $coef_{div} = 10$ $coef_{si} = 1$ $coef_{se} = 5$	FMO analysis (1.0)	Sigmoid function $coef_{high} = 0$ $coef_{low} = 1.25$ $coef_{k} = 2.5$	Degree of conjugation (1.0)	Sigmoid function $coef_{high} = 0.95$ $coef_{low} = 0.25$ $coef_{k} = 1$	Sum
Default	[*;r3-4,r7-17] [N;X3] [!#6][!#6] [CH2] [#6]=O	Triplet energy prediction (1.0)	Double sigmoid $coef_{high} = 77$ $coef_{low} = 63$ $coef_{div} = 5$ $coef_{si} = 2$ $coef_{se} = 2$	Computational prediction of maximum absorption wavelength (1.0)	Sigmoid function $coef_{high} = 400$ $coef_{low} = 300$ $coef_{k} = 1$	FMO analysis (1.0)	Sigmoid function $coef_{high} = 0$ $coef_{low} = 1.25$ $coef_{k} = 2.5$	Degree of conjugation (1.0)	Sigmoid function $coef_{high} = 0.95$ $coef_{low} = 0.25$ $coef_{k} = 1$	Sum
Default	[*;r3-4,r7-17] [N;X3] [!#6][!#6] [CH2] [#6]=O	Triplet energy prediction (1.0)	Double sigmoid $coef_{high} = 77$ $coef_{low} = 63$ $coef_{div} = 5$ $coef_{si} = 2$ $coef_{se} = 2$	ML prediction of maximum absorption wavelength (1.0)	Double sigmoid $coef_{high} = 430$ $coef_{low} = 365$ $coef_{div} = 10$ $coef_{si} = 1$ $coef_{se} = 5$	Degree of conjugation (1.0)	Sigmoid function $coef_{high} = 0.95$ $coef_{low} = 0.25$ $coef_{k} = 1$			Sum

Default	[*;r3-4,r7-17]<br>[N;X3]<br>[!#6][!#6]<br>[CH2]<br>[#6]=O	Triplet energy prediction (1.0)	Double sigmoid<br>$coef_{high} = 77$<br>$coef_{low} = 63$<br>$coef_{div} = 5$<br>$coef_{si} = 2$<br>$coef_{se} = 2$	Computational prediction of maximum absorption wavelength (1.0)	Sigmoid function<br>$coef_{high} = 400$<br>$coef_{low} = 300$<br>$coef_{k} = 1$	Degree of conjugation (1.0)	Sigmoid function<br>$coef_{high} = 0.95$<br>$coef_{low} = 0.25$<br>$coef_{k} = 1$			Sum
Default	[*;r3-4,r7-17]<br>[N;X3]<br>[!#6][!#6]<br>[CH2]<br>[#6]=O<br>[OH]<br>[r](=N)	Triplet energy prediction (1.0)	Double sigmoid<br>$coef_{high} = 77$<br>$coef_{low} = 63$<br>$coef_{div} = 5$<br>$coef_{si} = 2$<br>$coef_{se} = 2$	ML prediction of maximum absorption wavelength (1.0)	Double sigmoid<br>$coef_{high} = 430$<br>$coef_{low} = 345$<br>$coef_{div} = 10$<br>$coef_{si} = 1$<br>$coef_{se} = 5$	Degree of conjugation (1.0)	Sigmoid function<br>$coef_{high} = 0.95$<br>$coef_{low} = 0.25$<br>$coef_{k} = 1$	FMO analysis (1.0)	Right step function<br>$k_{step} = 0.5$	Hypervolume
Default	[*;r3-4,r7-17]<br>[N;X3]<br>[!#6][!#6]<br>[CH2]<br>[#6]=O<br>[OH]<br>[r](=N)	Triplet energy prediction (1.0)	Double sigmoid<br>$coef_{high} = 77$<br>$coef_{low} = 63$<br>$coef_{div} = 5$<br>$coef_{si} = 2$<br>$coef_{se} = 2$	ML prediction of maximum absorption wavelength (1.0)	Double sigmoid<br>$coef_{high} = 430$<br>$coef_{low} = 345$<br>$coef_{div} = 10$<br>$coef_{si} = 1$<br>$coef_{se} = 5$	Degree of conjugation (1.0)	Sigmoid function<br>$coef_{high} = 0.95$<br>$coef_{low} = 0.25$<br>$coef_{k} = 1$	FMO analysis (1.0)	Right step function<br>$k_{step} = 0.5$	Product

Default	[*;r3-4,r7-17]<br>[N;X3]<br>[!#6][!#6]<br>[CH2]	Triplet energy prediction (1.0)	Double sigmoid<br>$coef_{high} = 77$<br>$coef_{low} = 63$<br>$coef_{div} = 5$<br>$coef_{si} = 2$<br>$coef_{se} = 2$	Computational prediction of maximum absorption wavelength (1.0)	Sigmoid function<br>$coef_{high} = 400$<br>$coef_{low} = 300$<br>$coef_{k} = 1$	Degree of conjugation (1.0)	Sigmoid function<br>$coef_{high} = 0.95$<br>$coef_{low} = 0.25$<br>$coef_{k} = 1$			Sum
Default	[*;r3-4,r7-17]<br>[N;X3]<br>[!#6][!#6]<br>[CH2]<br>[#6]=O	Triplet energy prediction (1.0)	Double sigmoid<br>$coef_{high} = 77$<br>$coef_{low} = 63$<br>$coef_{div} = 5$<br>$coef_{si} = 2$<br>$coef_{se} = 2$	Computational prediction of maximum absorption wavelength (1.0)	Sigmoid function<br>$coef_{high} = 400$<br>$coef_{low} = 300$<br>$coef_{k} = 1$	Degree of conjugation (1.0)	Sigmoid function<br>$coef_{high} = 0.95$<br>$coef_{low} = 0.25$<br>$coef_{k} = 1$	FMO analysis (1.0)	Sigmoid function<br>$coef_{high} = 0$<br>$coef_{low} = 1.25$<br>$coef_{k} = 2.5$	Sum
Default	[*;r3-4,r7-17]<br>[N;X3]<br>[!#6][!#6]<br>[CH2]<br>[#6]=O<br>C=C	Triplet energy prediction (6.0)	Double sigmoid<br>$coef_{high} = 77$<br>$coef_{low} = 63$<br>$coef_{div} = 5$<br>$coef_{si} = 2$<br>$coef_{se} = 2$	ML prediction of maximum absorption wavelength (2.5)	Double sigmoid<br>$coef_{high} = 430$<br>$coef_{low} = 365$<br>$coef_{div} = 10$<br>$coef_{si} = 1$<br>$coef_{se} = 5$	FMO analysis (2.5)	Sigmoid function<br>$coef_{high} = 0$<br>$coef_{low} = 1.25$<br>$coef_{k} = 2.5$	Degree of conjugation (1.0)	Sigmoid function<br>$coef_{high} = 0.85$<br>$coef_{low} = 0.25$<br>$coef_{k} = 1$	Sum

Default	[*;r3-4,r7-17] [N;X3] [!#6][!#6]	Triplet energy prediction (1.0)	Double sigmoid $coef_{high} = 71$ $coef_{low} = 63$ $coef_{div} = 0.5$ $coef_{si} = 5$ $coef_{se} = 5$	ML prediction of maximum absorption wavelength (1.0)	Double sigmoid $coef_{high} = 430$ $coef_{low} = 300$ $coef_{div} = 10$ $coef_{si} = 5$ $coef_{se} = 5$	Degree of conjugation (1.0)	Sigmoid function $coef_{high} = 0.85$ $coef_{low} = 0.25$ $coef_{k} = 1$			Sum
Default	[*;r3-4,r7-17] [N;X3] [!#6][!#6] [CH2] [#6]=O	Triplet energy prediction (1.0)	Double sigmoid $coef_{high} = 77$ $coef_{low} = 63$ $coef_{div} = 5$ $coef_{si} = 2$ $coef_{se} = 2$	ML prediction of maximum absorption wavelength (1.0)	Double sigmoid $coef_{high} = 430$ $coef_{low} = 365$ $coef_{div} = 10$ $coef_{si} = 1$ $coef_{se} = 5$	FMO analysis (1.0)	Sigmoid function $coef_{high} = 0$ $coef_{low} = 1.25$ $coef_{k} = 2.5$			Product
Default	[*;r3-4,r7-17] [N;X3] [!#6][!#6] [CH2] [#6]=O	Triplet energy prediction (1.0)	Double sigmoid $coef_{high} = 77$ $coef_{low} = 63$ $coef_{div} = 5$ $coef_{si} = 2$ $coef_{se} = 2$	ML prediction of maximum absorption wavelength (1.0)	Double sigmoid $coef_{high} = 430$ $coef_{low} = 365$ $coef_{div} = 10$ $coef_{si} = 1$ $coef_{se} = 5$	FMO analysis (1.0)	Sigmoid function $coef_{high} = 0$ $coef_{low} = 1.25$ $coef_{k} = 2.5$			Hypervolume

Default	None	Triplet energy prediction (2.5)	Double sigmoid $coef_{high} = 77$ $coef_{low} = 63$ $coef_{div} = 5$ $coef_{si} = 2$ $coef_{se} = 2$	ML prediction of maximum absorption wavelength (2.5)	Double sigmoid $coef_{high} = 430$ $coef_{low} = 365$ $coef_{div} = 10$ $coef_{si} = 1$ $coef_{se} = 5$	FMO analysis (2.5)	Sigmoid function $coef_{high} = 0$ $coef_{low} = 1.25$ $coef_{k} = 2.5$	Rigidity (1.0)	Sigmoid function $coef_{high} = -1$ $coef_{low} = 8$ $coef_{k} = 0.5$	Product + Hypervolume
Default	[*;r3-4,r7-17] [N;X3] [!#6][!#6]	Triplet energy prediction (2.5)	Double sigmoid $coef_{high} = 71$ $coef_{low} = 63$ $coef_{div} = 0.5$ $coef_{si} = 5$ $coef_{se} = 5$	Computational prediction of maximum absorption wavelength (1.0)	Sigmoid function $coef_{high} = 400$ $coef_{low} = 300$ $coef_{k} = 1$	Degree of conjugation (1.0)	Sigmoid function $coef_{high} = 0.85$ $coef_{low} = 0.25$ $coef_{k} = 1$			Sum
Default	None	Triplet energy prediction (2.5)	Sigmoid function $coef_{high} = 65$ $coef_{low} = 58$ $coef_{k} = 0.6$	ML prediction of maximum absorption wavelength (2.5)	Double sigmoid $coef_{high} = 430$ $coef_{low} = 365$ $coef_{div} = 10$ $coef_{si} = 2.5$ $coef_{se} = 5$	Degree of conjugation (1.0)	Sigmoid function $coef_{high} = 0.95$ $coef_{low} = 0.25$ $coef_{k} = 1$			Product

Default	[*;r3-4,r7-17]<br>[N;X3]<br>[!#6][!#6]	Triplet energy prediction (2.5)	Double sigmoid<br>$coef_{high} = 68$<br>$coef_{low} = 63$<br>$coef_{div} = 1.5$<br>$coef_{si} = 2$<br>$coef_{se} = 2$	ML prediction of maximum absorption wavelength (2.5)	Double sigmoid<br>$coef_{high} = 430$<br>$coef_{low} = 360$<br>$coef_{div} = 10$<br>$coef_{si} = 5$<br>$coef_{se} = 5$					Sum
Default	[*;r3-4,r7-17]<br>[N;X3]<br>[!#6][!#6]<br>[CH2]<br>[#6]=O	Triplet energy prediction (1.0)	Double sigmoid<br>$coef_{high} = 77$<br>$coef_{low} = 63$<br>$coef_{div} = 5$<br>$coef_{si} = 2$<br>$coef_{se} = 2$	ML prediction of maximum absorption wavelength (1.0)	Double sigmoid<br>$coef_{high} = 430$<br>$coef_{low} = 365$<br>$coef_{div} = 10$<br>$coef_{si} = 1$<br>$coef_{se} = 5$	FMO analysis (1.0)	Sigmoid function<br>$coef_{high} = 0$<br>$coef_{low} = 1.25$<br>$coef_{k} = 2.5$			Sum
Default	[*;r3-4,r7-17]<br>[N;X3]<br>[!#6][!#6]<br>[CH2]<br>[#6]=O	Triplet energy prediction (1.0)	Double sigmoid<br>$coef_{high} = 77$<br>$coef_{low} = 63$<br>$coef_{div} = 5$<br>$coef_{si} = 2$<br>$coef_{se} = 2$	ML prediction of maximum absorption wavelength (1.0)	Double sigmoid<br>$coef_{high} = 430$<br>$coef_{low} = 365$<br>$coef_{div} = 10$<br>$coef_{si} = 1$<br>$coef_{se} = 5$	FMO analysis (1.0)	Sigmoid function<br>$coef_{high} = 0$<br>$coef_{low} = 1.25$<br>$coef_{k} = 2.5$			Product + Hypervolume

Default	[#6]=O<br>C=C	Triplet energy prediction (6.0)	Double sigmoid<br>$coef_{high} = 77$<br>$coef_{low} = 63$<br>$coef_{div} = 5$<br>$coef_{si} = 2$<br>$coef_{se} = 2$	Computational prediction of maximum absorption wavelength (2.5)	Sigmoid function<br>$coef_{high} = 400$<br>$coef_{low} = 300$<br>$coef_{k} = 1$	FMO analysis (1.0)	Sigmoid function<br>$coef_{high} = 0$<br>$coef_{low} = 1.25$<br>$coef_{k} = 2.5$	Degree of conjugation (1.0)	Sigmoid function<br>$coef_{high} = 0.85$<br>$coef_{low} = 0.25$<br>$coef_{k} = 1$	Sum
Default	None	Triplet energy prediction (2.5)	Double sigmoid<br>$coef_{high} = 77$<br>$coef_{low} = 63$<br>$coef_{div} = 5$<br>$coef_{si} = 2$<br>$coef_{se} = 2$	ML prediction of maximum absorption wavelength (2.5)	Double sigmoid<br>$coef_{high} = 430$<br>$coef_{low} = 365$<br>$coef_{div} = 10$<br>$coef_{si} = 1$<br>$coef_{se} = 5$	Rigidity (1.0)	Sigmoid function<br>$coef_{high} = -1$<br>$coef_{low} = 8$<br>$coef_{k} = 0.5$			Product + Hypervolume
Default	[*;r3-4,r7-17]<br>[N;X3]<br>[!#6][!#6]<br>[CH2]<br>[#6]=O<br>C=C	Triplet energy prediction (6.0)	Double sigmoid<br>$coef_{high} = 77$<br>$coef_{low} = 63$<br>$coef_{div} = 5$<br>$coef_{si} = 2$<br>$coef_{se} = 2$	Computational prediction of maximum absorption wavelength (2.5)	Sigmoid function<br>$coef_{high} = 400$<br>$coef_{low} = 300$<br>$coef_{k} = 1$	Degree of conjugation (1.0)	Sigmoid function<br>$coef_{high} = 0.85$<br>$coef_{low} = 0.25$<br>$coef_{k} = 1$			Sum

Default	[*;r3-4,r7-17]<br>[N;X3]<br>[!#6][!#6]<br>[CH2]	Triplet energy prediction (2.5)	Double sigmoid<br>$coef_{high} = 77$<br>$coef_{low} = 63$<br>$coef_{div} = 5$<br>$coef_{si} = 2$<br>$coef_{se} = 2$	ML prediction of maximum absorption wavelength (2.5)	Double sigmoid<br>$coef_{high} = 430$<br>$coef_{low} = 365$<br>$coef_{div} = 10$<br>$coef_{si} = 1$<br>$coef_{se} = 5$	SA score (1.0)	Sigmoid function<br>$coef_{high} = 1$<br>$coef_{low} = 10$<br>$coef_{k} = 0.5$			Hypervolume
Default	None	Triplet energy prediction (1.0)	Sigmoid function<br>$coef_{high} = 65$<br>$coef_{low} = 58$<br>$coef_{k} = 0.6$	ML prediction of maximum absorption wavelength (1.0)	Double sigmoid<br>$coef_{high} = 430$<br>$coef_{low} = 365$<br>$coef_{div} = 10$<br>$coef_{si} = 2.5$<br>$coef_{se} = 5$	Degree of conjugation (1.0)	Sigmoid function<br>$coef_{high} = 0.85$<br>$coef_{low} = 0.25$<br>$coef_{k} = 1$			Product + Hypervolume
Default	None	Triplet energy prediction (1.0)	Sigmoid function<br>$coef_{high} = 65$<br>$coef_{low} = 58$<br>$coef_{k} = 0.6$	ML prediction of maximum absorption wavelength (1.0)	Double sigmoid<br>$coef_{high} = 430$<br>$coef_{low} = 365$<br>$coef_{div} = 10$<br>$coef_{si} = 2.5$<br>$coef_{se} = 5$	Degree of conjugation (1.0)	Sigmoid function<br>$coef_{high} = 0.95$<br>$coef_{low} = 0.25$<br>$coef_{k} = 1$			Product

Default	None	Triplet energy prediction (1.0)	Sigmoid function $coef_{high} = 65$ $coef_{low} = 58$ $coef_k = 0.6$	Computational prediction of maximum absorption wavelength (2.5)	Double sigmoid $coef_{high} = 430$ $coef_{low} = 365$ $coef_{div} = 10$ $coef_{si} = 2.5$ $coef_{se} = 5$	Degree of conjugation (1.0)	Sigmoid function $coef_{high} = 0.95$ $coef_{low} = 0.25$ $coef_k = 1$			Product
Default	None	Triplet energy prediction (1.0)	Sigmoid function $coef_{high} = 65$ $coef_{low} = 58$ $coef_k = 0.6$	ML prediction of maximum absorption wavelength (1.0)	Double sigmoid $coef_{high} = 430$ $coef_{low} = 365$ $coef_{div} = 10$ $coef_{si} = 2.5$ $coef_{se} = 5$	Degree of conjugation (1.0)	Sigmoid function $coef_{high} = 0.95$ $coef_{low} = 0.25$ $coef_k = 1$			Hypervolume
Default	[*;r3-4,r7-17] [N;X3] [!#6][!#6] [CH2] [#6]=O C=C [OH]	Triplet energy prediction (3.0)	Double sigmoid $coef_{high} = 75$ $coef_{low} = 55$ $coef_{div} = 1$ $coef_{si} = 0.5$ $coef_{se} = 5$	ML prediction of maximum absorption wavelength (2.0)	Double sigmoid $coef_{high} = 430$ $coef_{low} = 330$ $coef_{div} = 10$ $coef_{si} = 0.5$ $coef_{se} = 5$	FMO analysis (1.0)	Right step function $k_{step} = 0.5$	Degree of conjugation (1.0)	Sigmoid function $coef_{high} = 0.85$ $coef_{low} = 0.25$ $coef_k = 1$	Product

Default	[*;r3-4,r7-17]<br>[N;X3]<br>[!#6][!#6]<br>[CH2]<br>[#6]=O<br>C=C<br>[OH]	Triplet energy prediction (1.5)	Double sigmoid<br>$coef_{high} = 75$<br>$coef_{low} = 55$<br>$coef_{div} = 1$<br>$coef_{si} = 0.5$<br>$coef_{se} = 5$	ML prediction of maximum absorption wavelength (2.0)	Double sigmoid<br>$coef_{high} = 430$<br>$coef_{low} = 330$<br>$coef_{div} = 10$<br>$coef_{si} = 0.5$<br>$coef_{se} = 5$	Degree of conjugation (1.0)	Sigmoid function<br>$coef_{high} = 0.85$<br>$coef_{low} = 0.25$<br>$coef_{k} = 1$			Product
Default	[*;r3-4,r7-17]<br>[N;X3]<br>[!#6][!#6]<br>[CH2]<br>[#6]=O<br>C=C<br>[OH]	Triplet energy prediction (3.0)	Double sigmoid<br>$coef_{high} = 75$<br>$coef_{low} = 55$<br>$coef_{div} = 1$<br>$coef_{si} = 0.5$<br>$coef_{se} = 5$	ML prediction of maximum absorption wavelength (2.0)	Double sigmoid<br>$coef_{high} = 430$<br>$coef_{low} = 330$<br>$coef_{div} = 10$<br>$coef_{si} = 0.5$<br>$coef_{se} = 5$	FMO analysis (1.0)	Right step function<br>$k_{step} = 0.5$	Degree of conjugation (1.0)	Sigmoid function<br>$coef_{high} = 0.85$<br>$coef_{low} = 0.25$<br>$coef_{k} = 1$	Hypervolume
Default	[*;r3-4,r7-17]<br>[N;X3]<br>[!#6][!#6]<br>[CH2]<br>[#6]=O<br>C=C<br>[OH]	Triplet energy prediction (1.5)	Double sigmoid<br>$coef_{high} = 75$<br>$coef_{low} = 55$<br>$coef_{div} = 1$<br>$coef_{si} = 0.5$<br>$coef_{se} = 5$	ML prediction of maximum absorption wavelength (2.0)	Double sigmoid<br>$coef_{high} = 430$<br>$coef_{low} = 330$<br>$coef_{div} = 10$<br>$coef_{si} = 0.5$<br>$coef_{se} = 5$	Degree of conjugation (1.0)	Sigmoid function<br>$coef_{high} = 0.85$<br>$coef_{low} = 0.25$<br>$coef_{k} = 1$			Hypervolume

Default	[*;r3-4,r7-17] [N;X3] [!#6][!#6] [CH2] [#6]=O C=C [OH]	Triplet energy prediction (3.0)	Double sigmoid $coef_{high} = 75$ $coef_{low} = 55$ $coef_{div} = 1$ $coef_{si} = 0.5$ $coef_{se} = 5$	Computational prediction of maximum absorption wavelength (2.0)	Sigmoid function $coef_{high} = 400$ $coef_{low} = 300$ $coef_k = 1$	FMO analysis (1.0)	Right step function $k_{step} = 0.5$	Degree of conjugation (1.0)	Sigmoid function $coef_{high} = 0.85$ $coef_{low} = 0.25$ $coef_k = 1$	Product
Default	[*;r3-4,r7-17] [N;X3] [!#6][!#6] [CH2] [#6]=O C=C [OH]	Triplet energy prediction (1.5)	Double sigmoid $coef_{high} = 75$ $coef_{low} = 55$ $coef_{div} = 1$ $coef_{si} = 0.5$ $coef_{se} = 5$	Computational prediction of maximum absorption wavelength (1.0)	Sigmoid function $coef_{high} = 400$ $coef_{low} = 300$ $coef_k = 1$	Degree of conjugation (1.0)	Sigmoid function $coef_{high} = 0.85$ $coef_{low} = 0.25$ $coef_k = 1$			Product
Default	None	Triplet energy prediction (1.0)	Sigmoid function $coef_{high} = 65$ $coef_{low} = 58$ $coef_k = 0.6$	ML prediction of maximum absorption wavelength (1.0)	Double sigmoid $coef_{high} = 430$ $coef_{low} = 330$ $coef_{div} = 10$ $coef_{si} = 2.5$ $coef_{se} = 5$	Degree of conjugation (1.0)	Sigmoid function $coef_{high} = 0.85$ $coef_{low} = 0.25$ $coef_k = 1$			Product

Default	[*;r3-4,r7-17]<br>[N;X3]<br>[!#6][!#6]<br>[CH2]<br>[#6]=O<br>C=C	Triplet energy prediction (6.0)	Double sigmoid<br>$coef_{high} = 77$<br>$coef_{low} = 63$<br>$coef_{div} = 5$<br>$coef_{si} = 2$<br>$coef_{se} = 2$	Computational prediction of maximum absorption wavelength (2.5)	Sigmoid function<br>$coef_{high} = 400$<br>$coef_{low} = 300$<br>$coef_{k} = 1$	Degree of conjugation (1.0)	Sigmoid function<br>$coef_{high} = 0.85$<br>$coef_{low} = 0.25$<br>$coef_{k} = 1$			Sum

1.5. Candidate selection

The generated candidates from all runs were concatenated and duplicates were removed (2,031,964 molecules). From this set, candidate molecules were selected which possessed a predicted triplet energy (ΔE_T) > 60 kcal/mol and a predicted maximum absorption wavelength > 350 nm when the ML prediction was employed ($\lambda_{max\text{-}ML}$) and > 325 nm when the semiempirical computational prediction was employed ($\lambda_{max\text{-}sTDA}$). This reduced the number of promising candidates to 11,775. **Figure S12** gives an overview of run parameters and their respective contribution to the final set of promising candidates.

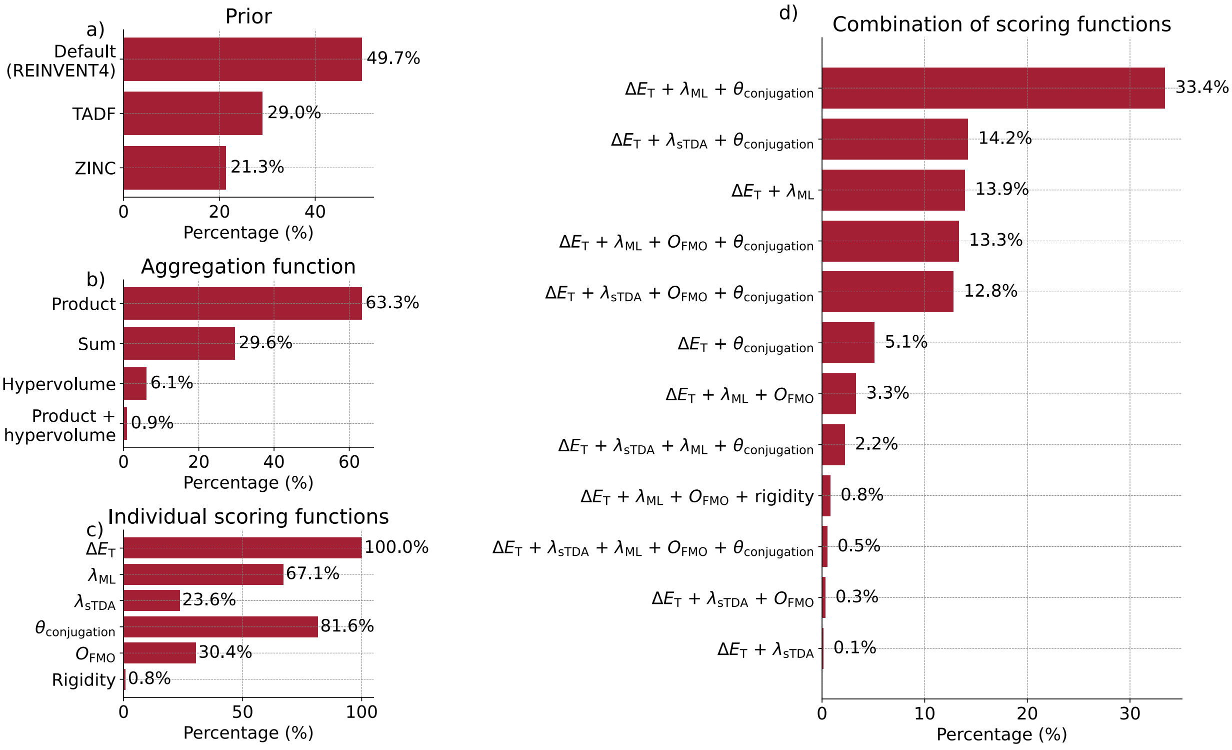

Figure S12: Analysis of the run parameters of the generative model during the multipronged chemical space exploration to generate the promising candidates. For each parameter its relative contribution, *e.g.*, fraction of generated molecules in the final set of promising candidates (11,775 molecules), is indicated. (a) The contribution of the different priors that were used for the generative model is shown. (b) The contribution of the different aggregation functions to obtain the final score is shown. (c) For each scoring component the fraction of promising candidates that were identified using this scoring component is given. (d) The contribution of combinations of scoring components that led to the generation of the promising candidates is given.

From this set of candidates, molecules were selected according to five distinct procedures:

Procedure 1:

- Select top 50% molecules based on $\lambda_{max\text{-}ML}$
- Generate extended circular fingerprint (ECFP, radius=2, 2048 bits)
- Cluster molecules and select the molecule with the highest $\lambda_{max\text{-}ML}$ from each of the 25 clusters

Procedure 2:

- Filter promising candidates to have a HOMO-LUMO overlap (O_{FMO}) < 0.5
- Select top 50% molecules based on $\lambda_{max\text{-}ML}$

- Generate extended circular fingerprint (ECFP, radius=2, 2048 bits)
- Cluster molecules and select the molecule with the highest $\lambda_{max\text{-}ML}$ from each of the 25 clusters

Procedure 3:

- Select top 50% molecules based on $\lambda_{max\text{-}sTDA}$
- Generate extended circular fingerprint (ECFP, radius=2, 2048 bits)
- Cluster molecules and select the molecule with the highest $\lambda_{max\text{-}sTDA}$ from each of the 25 clusters

Procedure 4:

- Filter promising candidates to have a HOMO-LUMO overlap (O_{FMO}) < 0.5
- Select top 50% molecules based on $\lambda_{max\text{-}sTDA}$
- Generate extended circular fingerprint (ECFP, radius=2, 2048 bits)
- Cluster molecules and select the molecule with the highest $\lambda_{max\text{-}sTDA}$ from each of the 25 clusters

Procedure 5:

- Generate extended circular fingerprint (ECFP, radius=2, 2048 bits) of all promising candidates
- Cluster molecules into 100 distinct clusters
- From each cluster select the molecules that:
    - maximise ΔE_T,
    - maximise $\lambda_{max\text{-}ML}$
    - maximise $\lambda_{max\text{-}sTDA}$
    - minimise O_{FMO}

The selected candidates from all five procedures were concatenated and duplicates were removed which yielded a total of 423 candidates that were used for high-fidelity quantum mechanical calculations (**Figure S13**).

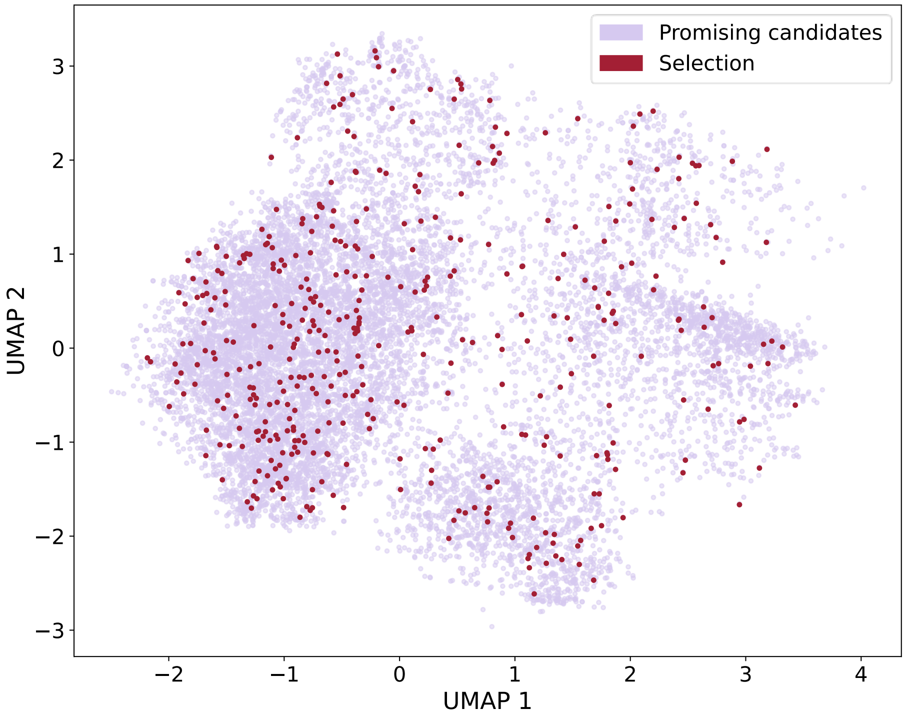

Figure S13: UMAP projection of promising candidates (11,775 molecules) and the selected molecules (423 molecules) based on their ECFP fingerprint (radius=2, 2048 bits)

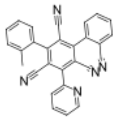
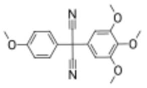
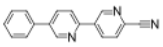
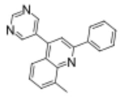
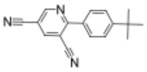
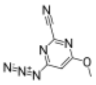
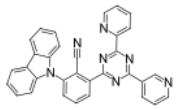
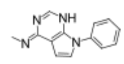
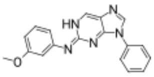
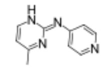
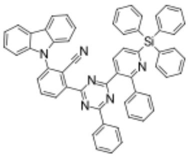
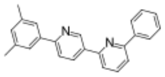
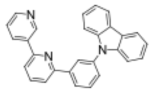
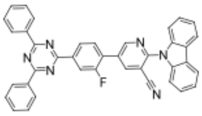
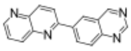
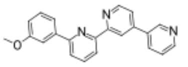
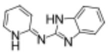
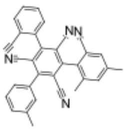
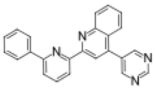
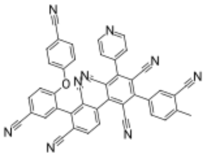
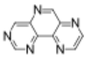
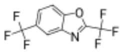
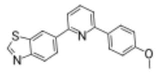
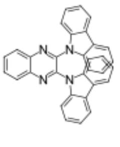
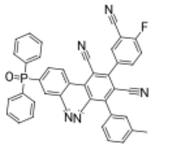
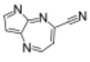
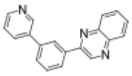
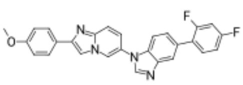
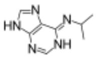
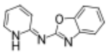

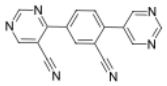
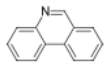
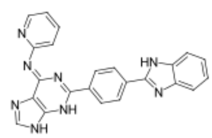
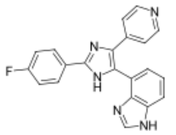
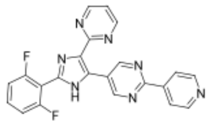
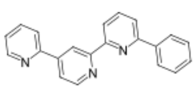
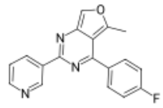
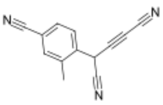
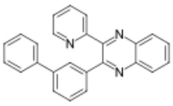
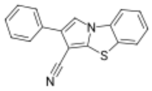
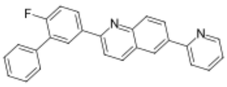
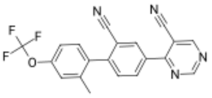
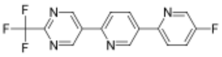
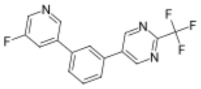
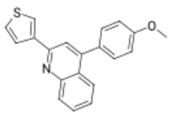
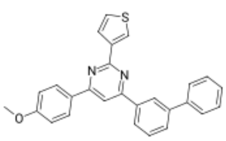
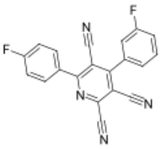
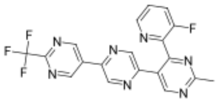
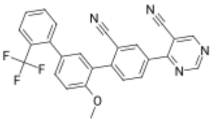
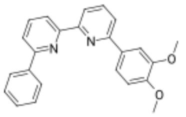
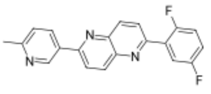
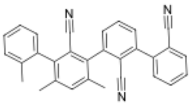
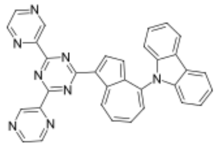
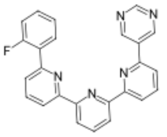
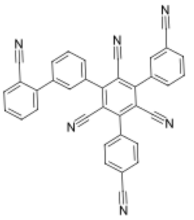
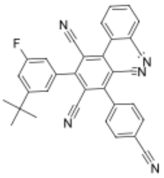
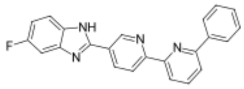
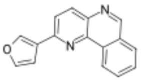
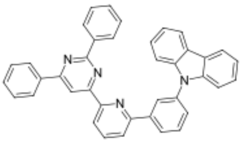
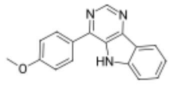

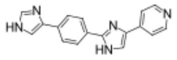 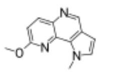 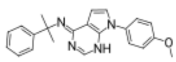 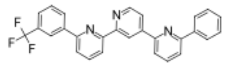 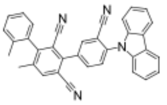

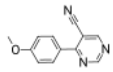 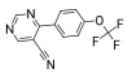 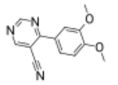 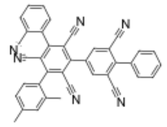 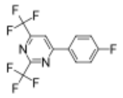

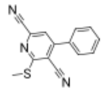 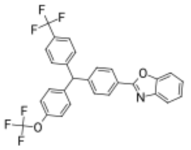 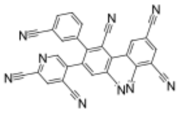 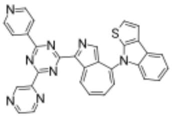 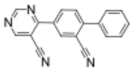

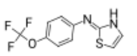 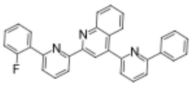 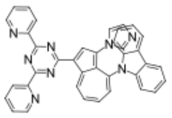 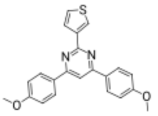 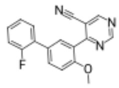

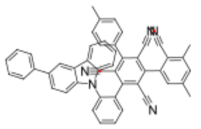 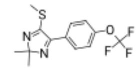 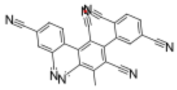 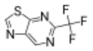 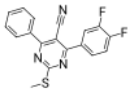

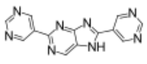 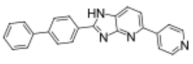 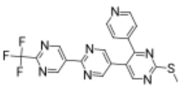 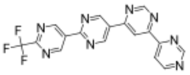 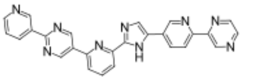

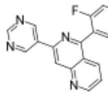
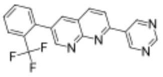
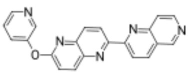
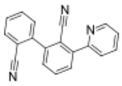
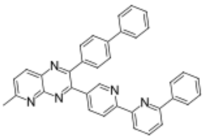
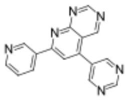
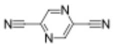
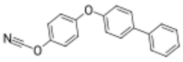
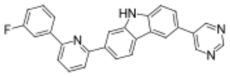
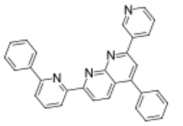
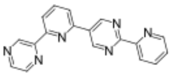
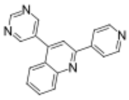
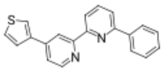
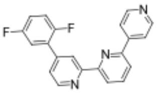
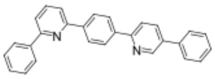
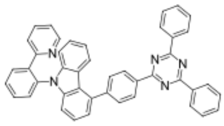
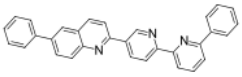
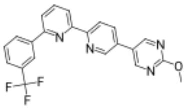
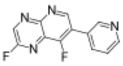
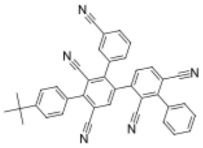
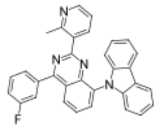
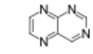
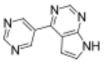
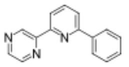
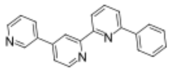
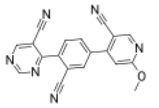
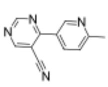
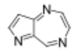
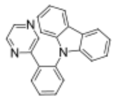
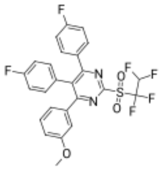

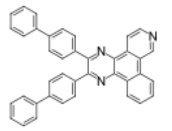
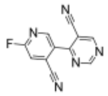
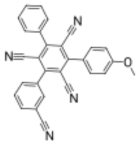
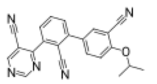
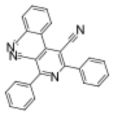

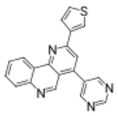
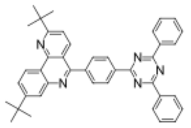
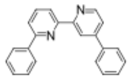
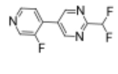
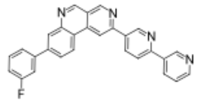

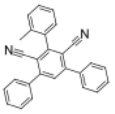
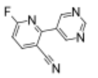
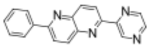
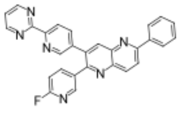
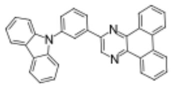

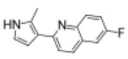
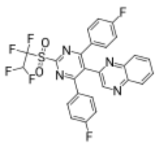
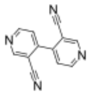
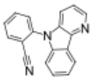
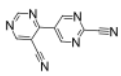

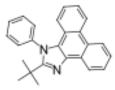
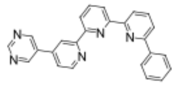
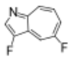
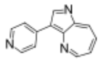
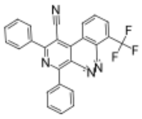

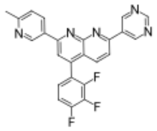
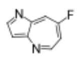
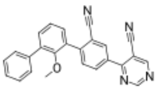
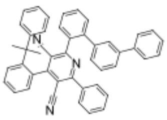
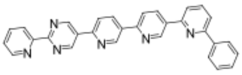

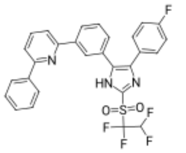
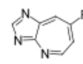
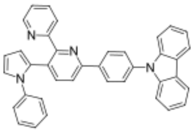
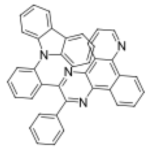
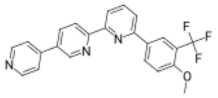
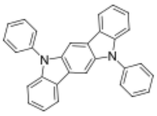
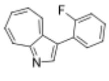
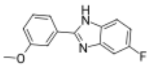
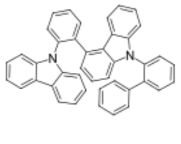
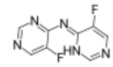
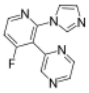
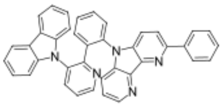
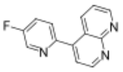
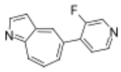
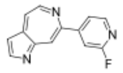
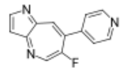
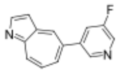
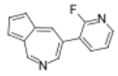
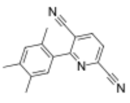
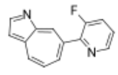
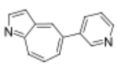
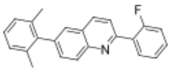
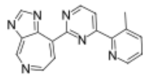
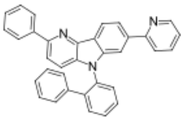
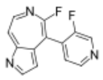
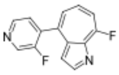
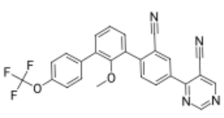
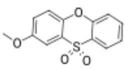
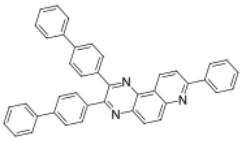
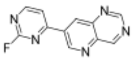

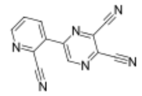
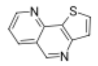
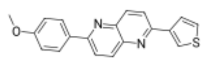
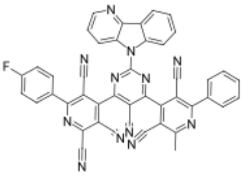
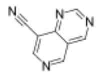
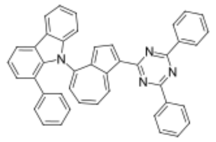
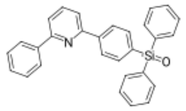
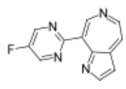
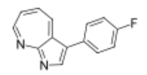
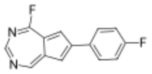
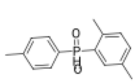
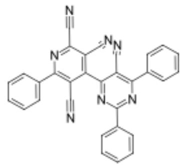
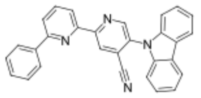
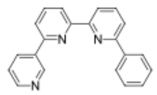
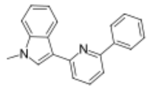
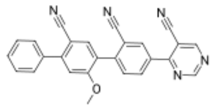
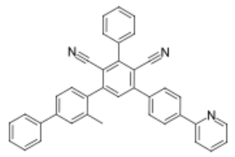
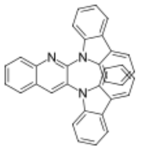
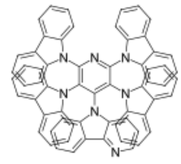
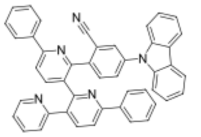
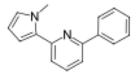
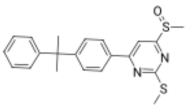
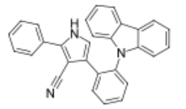
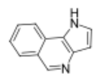
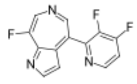
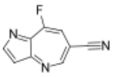
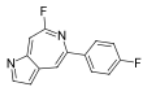
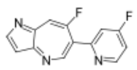
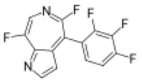
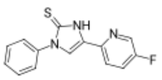

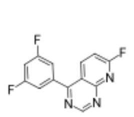
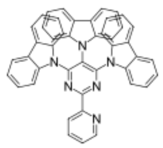
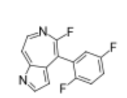
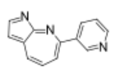
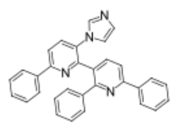
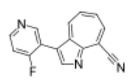
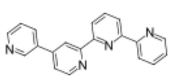
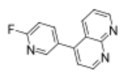
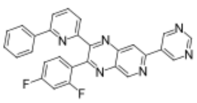
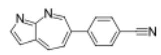
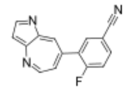
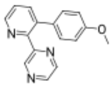
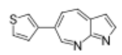
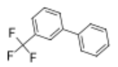
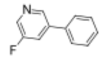
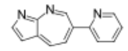
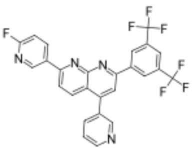
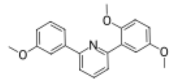
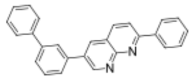
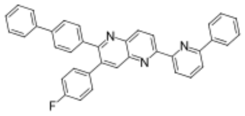
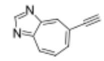
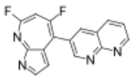
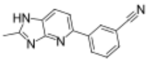
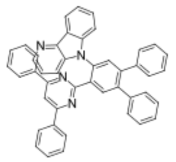
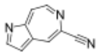
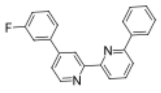
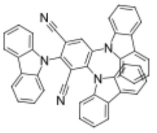
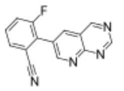
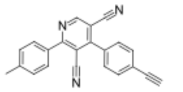
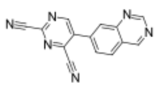

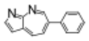
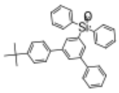
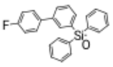
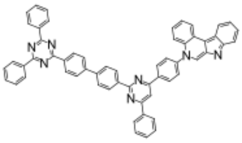
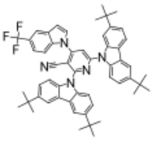
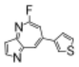
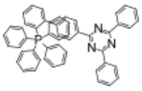
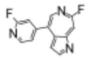
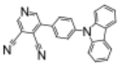
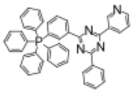
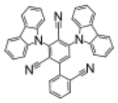
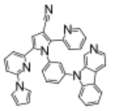
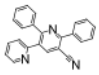
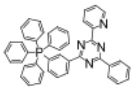
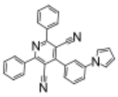
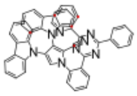
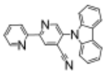
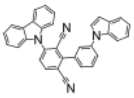
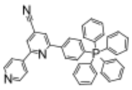
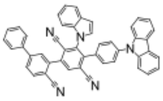
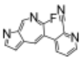
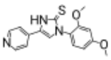
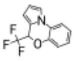
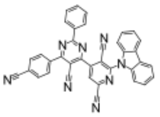
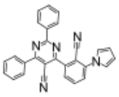
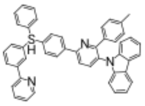
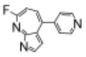
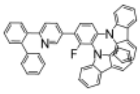
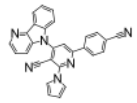
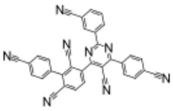

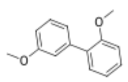 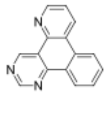 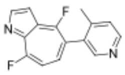 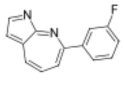 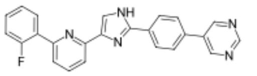

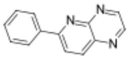 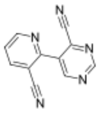 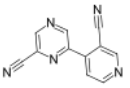 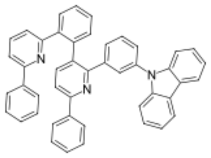 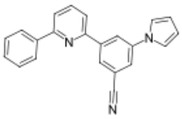

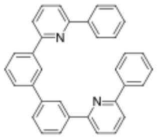 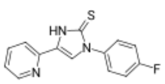 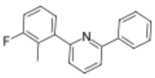 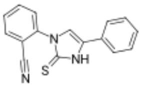 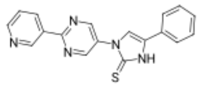

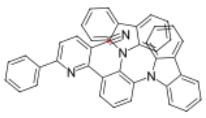 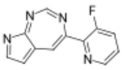 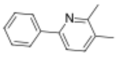 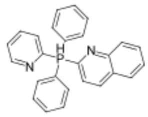 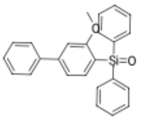

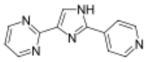 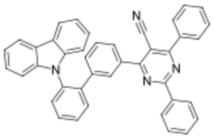 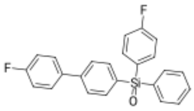 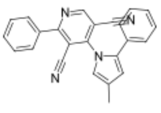 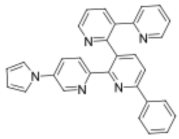

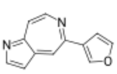 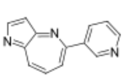 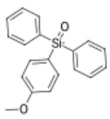 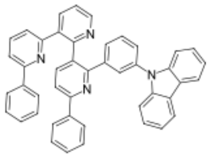 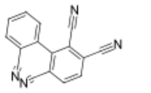

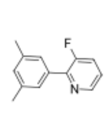
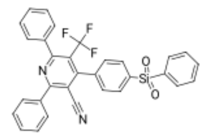
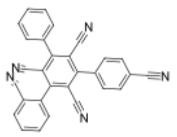
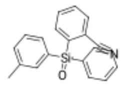
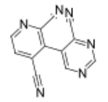
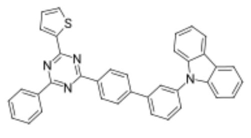
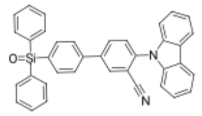
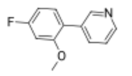
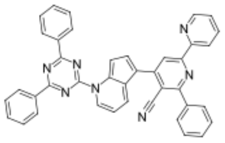
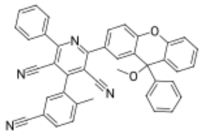
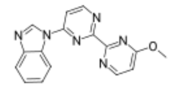
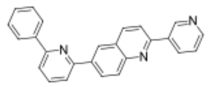
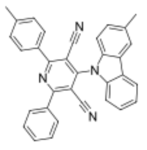
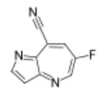
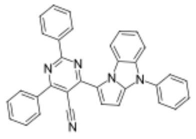
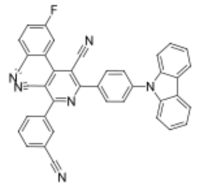
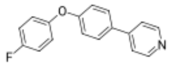
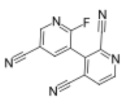
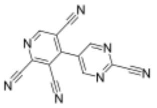
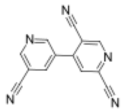
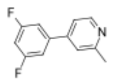
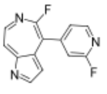
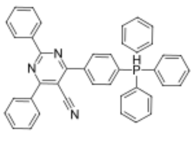
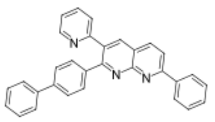
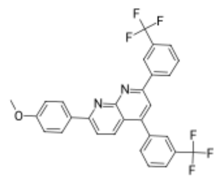
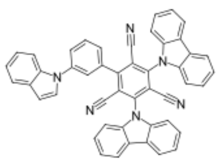
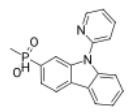
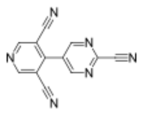
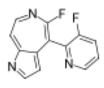
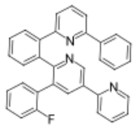

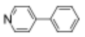
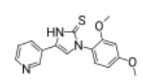
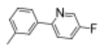
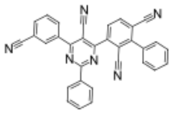
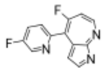
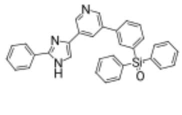
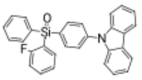
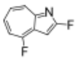
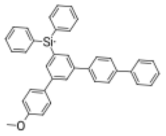
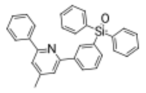
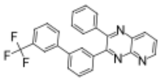
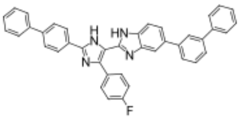
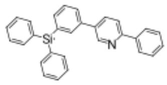
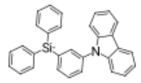
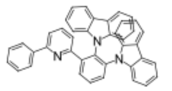
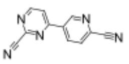
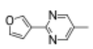
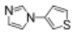
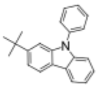
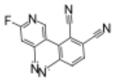
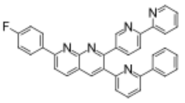
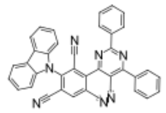
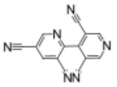
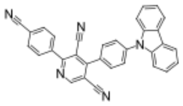
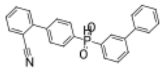
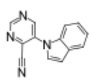
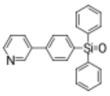
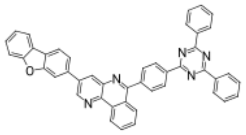
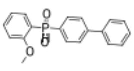
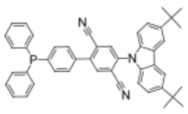

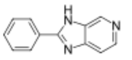 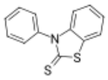 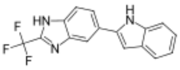 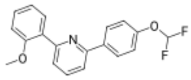 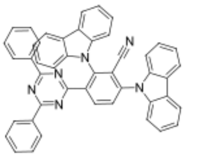

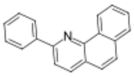 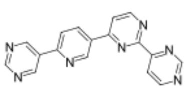 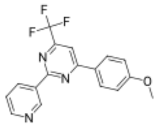 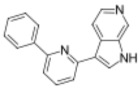 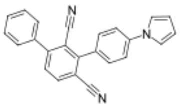

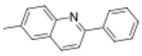 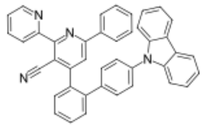 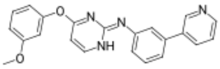 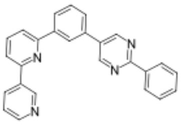 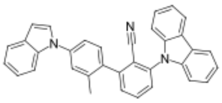

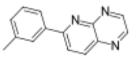 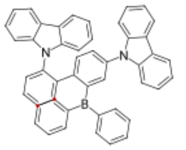 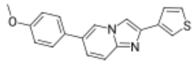 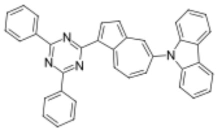 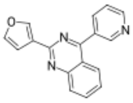

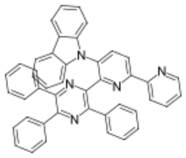 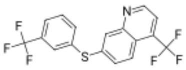 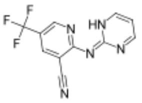 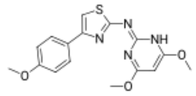 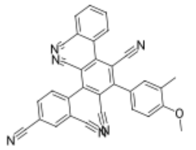

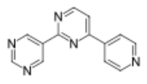 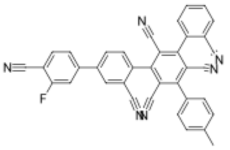 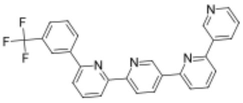 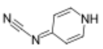 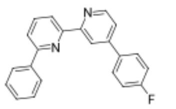

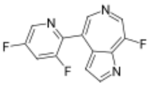 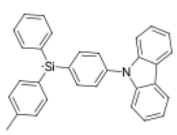 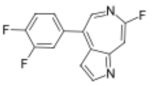

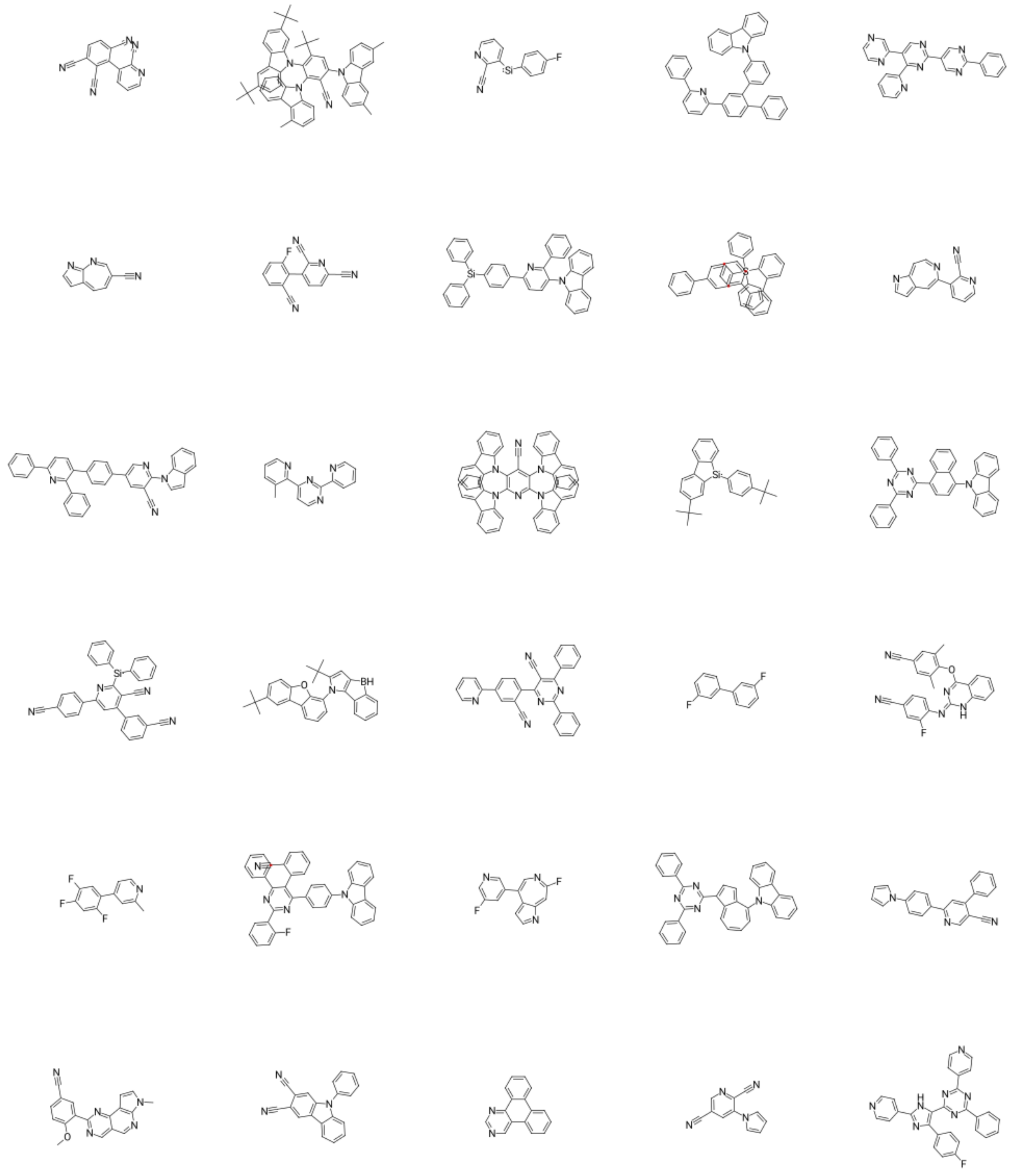

Figure S14: 432 selected candidates for QM calculations.

2. QUANTUM MECHANICAL CALCULATIONS

Starting from the SMILES representation of a molecule, a 3D structure was obtained using RDKit.[26] The conformational ensemble average was approximated by the molecule's lowest energy conformer using the

ETKDGv3 algorithm as implemented in RDKit.[30] The lowest energy conformer was pre-optimized on the semi-empirical GFN2-xTB level.[12] The pre-optimized structure was then optimized for the S_0, S_1, and T_1 state using ωB97X-D3//def2-SVP level of theory as implemented in ORCA 6.0.0 applying the CPCM continuum solvation model for acetonitrile.[31–36] For S_0 and T_1, the optimization was performed within the Kohn–Sham (KS) DFT formalism, while for S_1, TD-DFT optimizations were carried out. In order to accelerate the computation of two electron integrals, the resolution of identity approximation was used for the Coulomb part (RIJ) and the chain of spheres algorithm for the exchange part (COSX), with the corresponding auxiliary basis and grid settings.[37,38] Minimum energy structures were confirmed through frequency calculations (also for excited states) and single point energies were computed on the ωB97X-D3//def2-TZVPP level of theory using the same solvation model.[39] The triplet energy was obtained as the adiabatic singlet-triplet energy gap, to which free energy corrections (zero-point vibrational energy corrections, thermal corrections, enthalpy corrections, entropic corrections) as obtained from frequency calculations at 298.15 K were added. The absorption spectrum was obtained using the adiabatic hessian and the inclusion of the Herzberg–Teller effect. From the resulting output, excitation wavelengths and oscillator strengths were extracted. Each transition was broadened using Gaussian functions, simulating the absorption spectrum. The maximum absorption wavelength was obtained using a threshold for peak identification of 0.00001. This was performed using a modified version of the *orca_uv* code which is publicly available on GitHub.[40] The fluorescence and ISC and intersystem crossing rate (ISC) were obtained using the excited state dynamics (ESD) module in ORCA including the Herzberg–Teller effect.[41,42] The ISC rate was computed including Duschinsky mixing[43] at a temperature of 77 K and obtained as the average rate across all three spin-sublevels.

2.1. Validation of predicted ISC yield

No direct correlation between the computed ratio of ISC (k_{ISC}) and fluorescence rate (k_f) with reported ISC quantum yields (Φ_{ISC}) (48 molecules, data available in GitHub repository)[6,10] could be observed. However, for molecules with $\frac{\log(k_{ISC})}{\log(k_f)} > 1$ high Φ_{ISC} are reported, indicating that $\frac{\log(k_{ISC})}{\log(k_f)}$ can serve as a predictor for efficient triplet population through ISC (**Figure S15**). This observation can be rationalized by the fact that transitions between S_1 and higher lying triplets may contribute to Φ_{ISC}. The computed k_{ISC}, however, only considers the rate from S_1 to T_1. Moreover, alternative pathways detrimental for Φ_{ISC} such as internal conversion are not considered in the computation.

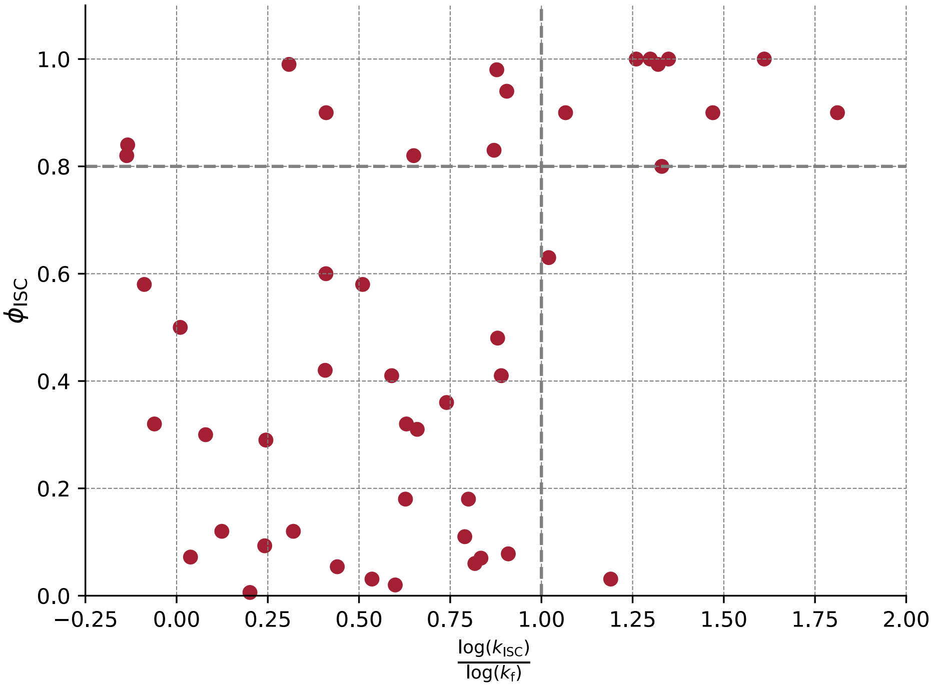

Figure S15: Parity plot of reported ISC quantum yield (Φ_{ISC}) and the ratio of computed ISC rate and computed fluorescence rate (48 molecules).

2.2. Automated workflow

Three key properties were obtained from the automated computations pipeline: ΔE_T, $\lambda_{max\text{-}QM}$, and $\frac{\log(k_{ISC})}{\log(k_f)}$. To obtain these properties multiple (interdependent) steps, *e.g.*, geometry optimization, single point energy, excited state dynamics, were required. Therefore, a time limit of 120 hours per molecule was set, to keep the computational effort tractable. Each individual step had a time limit of 24 hours. Geometry optimizations that converged to structures with imaginary frequencies were resubmitted up to three times. Using this workflow a complete set of properties (ΔE_T, $\lambda_{max\text{-}QM}$, and $\frac{\log(k_{ISC})}{\log(k_f)}$) could be obtained for 129 out of 432 molecules (30%, **Figure S16**). Out of these molecules, candidates with a ΔE_T > 55 kcal/mol, $\lambda_{max\text{-}QM}$ > 250 nm and $\frac{\log(k_{ISC})}{\log(k_f)}$ > 0.75 were considered as the refined selection (15 molecules, **Figure S19**).

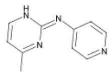

Triplet energy = 54.9 kcal/mol
Absorption maximum = 261 nm
Fluorescence rate = 1.73e+08 s-1
ISC rate = 7.05e+06 s-1

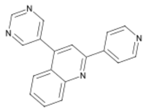

Triplet energy = 57.9 kcal/mol
Absorption maximum = 233 nm
Fluorescence rate = 7.69e+08 s-1
ISC rate = 3.36e+07 s-1

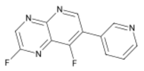

Triplet energy = 61.0 kcal/mol
Absorption maximum = 275 nm
Fluorescence rate = 5.75e+07 s-1
ISC rate = 8.03e+06 s-1

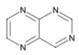

Triplet energy = 61.1 kcal/mol
Absorption maximum = 348 nm
Fluorescence rate = 2.26e+06 s-1
ISC rate = 1.75e+06 s-1

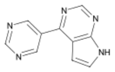

Triplet energy = 57.6 kcal/mol
Absorption maximum = 279 nm
Fluorescence rate = 2.70e+08 s-1
ISC rate = 6.96e+04 s-1

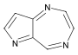

Triplet energy = 33.7 kcal/mol
Absorption maximum = 404 nm
Fluorescence rate = 1.06e+07 s-1
ISC rate = 5.76e+05 s-1

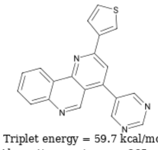

Triplet energy = 59.7 kcal/mol
Absorption maximum = 295 nm
Fluorescence rate = 5.18e+08 s-1
ISC rate = 9.51e+06 s-1

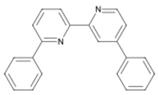

Triplet energy = 67.5 kcal/mol
Absorption maximum = 190 nm
Fluorescence rate = 1.05e+09 s-1
ISC rate = 4.97e+06 s-1

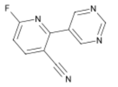

Triplet energy = 65.8 kcal/mol
Absorption maximum = 168 nm
Fluorescence rate = 7.34e+09 s-1
ISC rate = 2.35e+07 s-1

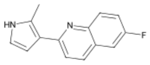

Triplet energy = 59.5 kcal/mol
Absorption maximum = 301 nm
Fluorescence rate = 4.95e+08 s-1
ISC rate = 3.87e+06 s-1

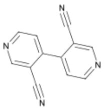

Triplet energy = 71.9 kcal/mol
Absorption maximum = 193 nm
Fluorescence rate = 8.22e+07 s-1
ISC rate = 1.53e+07 s-1

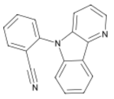

Triplet energy = 72.6 kcal/mol
Absorption maximum = 262 nm
Fluorescence rate = 6.80e+08 s-1
ISC rate = 5.14e+05 s-1

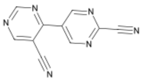

Triplet energy = 71.1 kcal/mol
Absorption maximum = 176 nm
Fluorescence rate = 1.50e+07 s-1
ISC rate = 3.43e+06 s-1

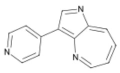

Triplet energy = 32.6 kcal/mol
Absorption maximum = 434 nm
Fluorescence rate = 1.02e+07 s-1
ISC rate = 7.55e+05 s-1

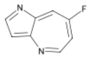

Triplet energy = 34.1 kcal/mol
Absorption maximum = 430 nm
Fluorescence rate = 1.42e+07 s-1
ISC rate = 8.15e+03 s-1

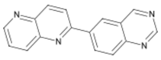

Triplet energy = 57.2 kcal/mol
Absorption maximum = 291 nm
Fluorescence rate = 1.58e+09 s-1
ISC rate = 7.03e+06 s-1

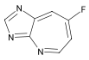

Triplet energy = 45.3 kcal/mol
Absorption maximum = 352 nm
Fluorescence rate = 2.56e+07 s-1
ISC rate = 1.56e+08 s-1

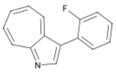

Triplet energy = 38.2 kcal/mol
Absorption maximum = 416 nm
Fluorescence rate = 2.60e+07 s-1
ISC rate = 1.48e+04 s-1

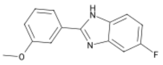

Triplet energy = 65.2 kcal/mol
Absorption maximum = 277 nm
Fluorescence rate = 9.32e+08 s-1
ISC rate = 2.70e+03 s-1

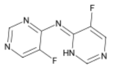

Triplet energy = 60.5 kcal/mol
Absorption maximum = 307 nm
Fluorescence rate = 9.11e+08 s-1
ISC rate = 2.93e+06 s-1

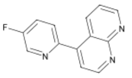

Triplet energy = 61.8 kcal/mol
Absorption maximum = 258 nm
Fluorescence rate = 1.33e+08 s-1
ISC rate = 5.84e+06 s-1

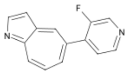

Triplet energy = 39.3 kcal/mol
Absorption maximum = 408 nm
Fluorescence rate = 3.37e+07 s-1
ISC rate = 3.68e+04 s-1

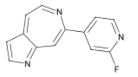

Triplet energy = 33.7 kcal/mol
Absorption maximum = 444 nm
Fluorescence rate = 3.00e+07 s-1
ISC rate = 8.94e+06 s-1

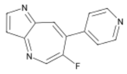

Triplet energy = 34.5 kcal/mol
Absorption maximum = 410 nm
Fluorescence rate = 1.52e+06 s-1
ISC rate = 9.34e+04 s-1

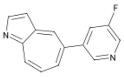

Triplet energy = 39.2 kcal/mol
Absorption maximum = 412 nm
Fluorescence rate = 4.24e+07 s-1
ISC rate = 2.06e+05 s-1

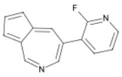

Triplet energy = 35.4 kcal/mol
Absorption maximum = 462 nm
Fluorescence rate = 8.70e+06 s-1
ISC rate = 1.93e+05 s-1

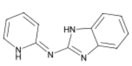

Triplet energy = 54.9 kcal/mol
Absorption maximum = 317 nm
Fluorescence rate = 6.83e+08 s-1
ISC rate = 5.75e+05 s-1

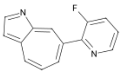

Triplet energy = 38.9 kcal/mol
Absorption maximum = 408 nm
Fluorescence rate = 2.87e+07 s-1
ISC rate = 3.98e+05 s-1

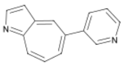

Triplet energy = 39.4 kcal/mol
Absorption maximum = 412 nm
Fluorescence rate = 3.73e+07 s-1
ISC rate = 3.85e+04 s-1

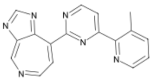

Triplet energy = 47.0 kcal/mol
Absorption maximum = 239 nm
Fluorescence rate = 4.07e+07 s-1
ISC rate = 3.01e+03 s-1

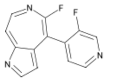

Triplet energy = 31.5 kcal/mol
Absorption maximum = 454 nm
Fluorescence rate = 2.58e+07 s-1
ISC rate = 5.13e+04 s-1

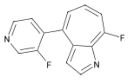

Triplet energy = 40.1 kcal/mol
Absorption maximum = 394 nm
Fluorescence rate = 2.54e+07 s-1
ISC rate = 1.25e+04 s-1

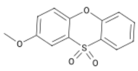

Triplet energy = 69.0 kcal/mol
Absorption maximum = 194 nm
Fluorescence rate = 2.87e+08 s-1
ISC rate = 4.31e+06 s-1

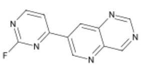

Triplet energy = 63.8 kcal/mol
Absorption maximum = 267 nm
Fluorescence rate = 7.21e+06 s-1
ISC rate = 4.76e+08 s-1

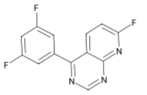

Triplet energy = 65.4 kcal/mol
Absorption maximum = 255 nm
Fluorescence rate = 2.64e+07 s-1
ISC rate = 2.06e+06 s-1

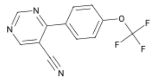

Triplet energy = 68.3 kcal/mol
Absorption maximum = 253 nm
Fluorescence rate = 9.45e+07 s-1
ISC rate = 3.83e+03 s-1

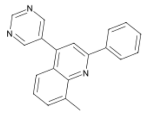

Triplet energy = 56.4 kcal/mol
Absorption maximum = 241 nm
Fluorescence rate = 5.73e+08 s-1
ISC rate = 4.07e+06 s-1

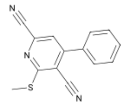

Triplet energy = 57.4 kcal/mol
Absorption maximum = 303 nm
Fluorescence rate = 2.89e+08 s-1
ISC rate = 4.28e+07 s-1

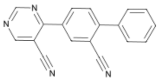

Triplet energy = 65.2 kcal/mol
Absorption maximum = 261 nm
Fluorescence rate = 5.29e+08 s-1
ISC rate = 4.41e+01 s-1

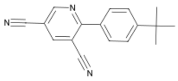

Triplet energy = 63.4 kcal/mol
Absorption maximum = 272 nm
Fluorescence rate = 6.15e+08 s-1
ISC rate = 3.83e+06 s-1

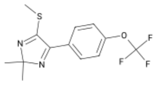

Triplet energy = 61.7 kcal/mol
Absorption maximum = 234 nm
Fluorescence rate = 5.00e+07 s-1
ISC rate = 9.50e+07 s-1

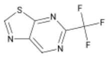

Triplet energy = 70.7 kcal/mol
Absorption maximum = 199 nm
Fluorescence rate = 5.99e+06 s-1
ISC rate = 2.53e+08 s-1

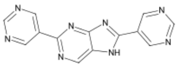

Triplet energy = 66.8 kcal/mol
Absorption maximum = 279 nm
Fluorescence rate = 9.82e+08 s-1
ISC rate = 1.76e+06 s-1

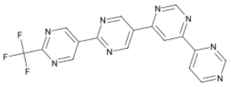

Triplet energy = 63.6 kcal/mol
Absorption maximum = 275 nm
Fluorescence rate = 4.45e+07 s-1
ISC rate = 1.34e+07 s-1

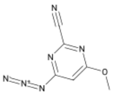

Triplet energy = 64.5 kcal/mol
Absorption maximum = 224 nm
Fluorescence rate = 7.68e+05 s-1
ISC rate = 1.91e+06 s-1

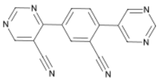

Triplet energy = 66.4 kcal/mol
Absorption maximum = 210 nm
Fluorescence rate = 1.30e+08 s-1
ISC rate = 2.26e+02 s-1

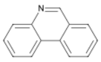

Triplet energy = 63.3 kcal/mol
Absorption maximum = 236 nm
Fluorescence rate = 1.32e+08 s-1
ISC rate = 1.48e+06 s-1

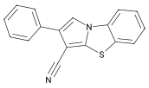

Triplet energy = 68.3 kcal/mol
Absorption maximum = 226 nm
Fluorescence rate = 6.75e+06 s-1
ISC rate = 3.96e+04 s-1

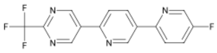

Triplet energy = 60.5 kcal/mol
Absorption maximum = 279 nm
Fluorescence rate = 7.60e+08 s-1
ISC rate = 5.89e+04 s-1

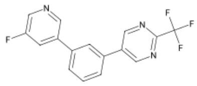

Triplet energy = 68.3 kcal/mol
Absorption maximum = 234 nm
Fluorescence rate = 3.65e+08 s-1
ISC rate = 4.17e+05 s-1

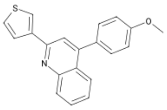

Triplet energy = 58.2 kcal/mol
Absorption maximum = 194 nm
Fluorescence rate = 3.78e+08 s-1
ISC rate = 2.94e+06 s-1

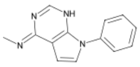

Triplet energy = 60.0 kcal/mol
Absorption maximum = 266 nm
Fluorescence rate = 3.84e+08 s-1
ISC rate = 3.50e+06 s-1

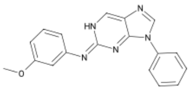

Triplet energy = 44.6 kcal/mol
Absorption maximum = 327 nm
Fluorescence rate = 7.95e+07 s-1
ISC rate = 1.15e+05 s-1

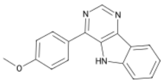

Triplet energy = 62.1 kcal/mol
Absorption maximum = 265 nm
Fluorescence rate = 5.78e+08 s-1
ISC rate = 8.97e+05 s-1

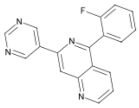

Triplet energy = 57.5 kcal/mol
Absorption maximum = 230 nm
Fluorescence rate = 3.24e+08 s-1
ISC rate = 1.59e+07 s-1

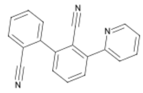

Triplet energy = 67.1 kcal/mol
Absorption maximum = 203 nm
Fluorescence rate = 2.31e+08 s-1
ISC rate = 2.41e+06 s-1

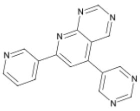

Triplet energy = 63.6 kcal/mol
Absorption maximum = 281 nm
Fluorescence rate = 7.92e+07 s-1
ISC rate = 1.42e+05 s-1

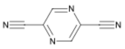

Triplet energy = 63.3 kcal/mol
Absorption maximum = 250 nm
Fluorescence rate = 1.49e+07 s-1
ISC rate = 1.86e+08 s-1

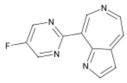

Triplet energy = 35.5 kcal/mol
Absorption maximum = 443 nm
Fluorescence rate = 3.27e+06 s-1
ISC rate = 9.40e+06 s-1

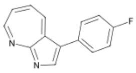

Triplet energy = 30.4 kcal/mol
Absorption maximum = 470 nm
Fluorescence rate = 1.28e+06 s-1
ISC rate = 4.38e+05 s-1

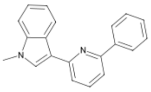

Triplet energy = 63.6 kcal/mol
Absorption maximum = 186 nm
Fluorescence rate = 3.18e+07 s-1
ISC rate = 4.75e+05 s-1

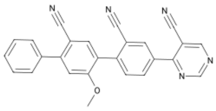

Triplet energy = 67.1 kcal/mol
Absorption maximum = 282 nm
Fluorescence rate = 8.85e+07 s-1
ISC rate = 4.51e+02 s-1

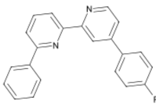

Triplet energy = 67.8 kcal/mol
Absorption maximum = 180 nm
Fluorescence rate = 2.34e+08 s-1
ISC rate = 1.52e+07 s-1

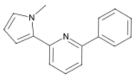

Triplet energy = 64.0 kcal/mol
Absorption maximum = 265 nm
Fluorescence rate = 2.26e+07 s-1
ISC rate = 1.64e+06 s-1

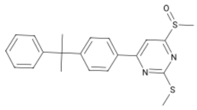

Triplet energy = 68.1 kcal/mol
Absorption maximum = 245 nm
Fluorescence rate = 6.18e+08 s-1
ISC rate = 2.02e+09 s-1

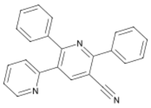

Triplet energy = 59.9 kcal/mol
Absorption maximum = 195 nm
Fluorescence rate = 9.78e+06 s-1
ISC rate = 4.92e+03 s-1

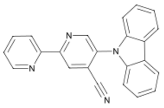

Triplet energy = 62.9 kcal/mol
Absorption maximum = 323 nm
Fluorescence rate = 1.31e+07 s-1
ISC rate = 8.91e+03 s-1

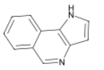

Triplet energy = 62.4 kcal/mol
Absorption maximum = 276 nm
Fluorescence rate = 1.86e+07 s-1
ISC rate = 1.59e+05 s-1

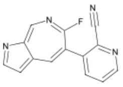

Triplet energy = 42.5 kcal/mol
Absorption maximum = 383 nm
Fluorescence rate = 1.18e+06 s-1
ISC rate = 3.65e+06 s-1

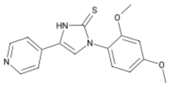

Triplet energy = 56.3 kcal/mol
Absorption maximum = 286 nm
Fluorescence rate = 6.92e+05 s-1
ISC rate = 2.80e+10 s-1

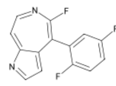

Triplet energy = 31.6 kcal/mol
Absorption maximum = 451 nm
Fluorescence rate = 1.76e+07 s-1
ISC rate = 3.60e+04 s-1

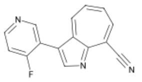

Triplet energy = 34.4 kcal/mol
Absorption maximum = 453 nm
Fluorescence rate = 2.38e+07 s-1
ISC rate = 2.54e+05 s-1

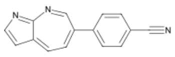

Triplet energy = 33.3 kcal/mol
Absorption maximum = 437 nm
Fluorescence rate = 3.56e+07 s-1
ISC rate = 6.91e+06 s-1

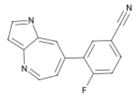

Triplet energy = 33.9 kcal/mol
Absorption maximum = 426 nm
Fluorescence rate = 1.15e+07 s-1
ISC rate = 2.63e+04 s-1

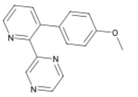

Triplet energy = 49.7 kcal/mol
Absorption maximum = 237 nm
Fluorescence rate = 2.14e+07 s-1
ISC rate = 2.36e-06 s-1

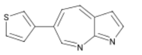

Triplet energy = 33.5 kcal/mol
Absorption maximum = 431 nm
Fluorescence rate = 4.87e+07 s-1
ISC rate = 8.40e+06 s-1

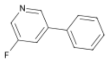

Triplet energy = 67.6 kcal/mol
Absorption maximum = 239 nm
Fluorescence rate = 1.55e+09 s-1
ISC rate = 2.14e+01 s-1

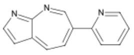

Triplet energy = 33.1 kcal/mol
Absorption maximum = 439 nm
Fluorescence rate = 2.78e+07 s-1
ISC rate = 1.07e+07 s-1

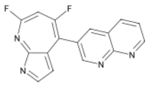

Triplet energy = 29.0 kcal/mol
Absorption maximum = 448 nm
Fluorescence rate = 1.93e+07 s-1
ISC rate = 5.70e+05 s-1

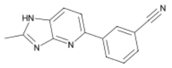

Triplet energy = 65.5 kcal/mol
Absorption maximum = 273 nm
Fluorescence rate = 1.08e+09 s-1
ISC rate = 3.35e+03 s-1

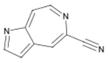

Triplet energy = 35.4 kcal/mol
Absorption maximum = 430 nm
Fluorescence rate = 3.96e+07 s-1
ISC rate = 1.10e+04 s-1

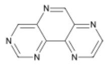

Triplet energy = 63.9 kcal/mol
Absorption maximum = 239 nm
Fluorescence rate = 9.80e+06 s-1
ISC rate = 1.64e+09 s-1

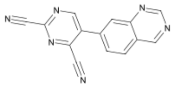

Triplet energy = 59.7 kcal/mol
Absorption maximum = 199 nm
Fluorescence rate = 2.54e+07 s-1
ISC rate = 2.17e+07 s-1

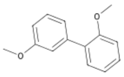

Triplet energy = 67.7 kcal/mol
Absorption maximum = 193 nm
Fluorescence rate = 3.39e+08 s-1
ISC rate = 7.39e+05 s-1

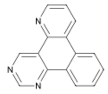

Triplet energy = 72.0 kcal/mol
Absorption maximum = 234 nm
Fluorescence rate = 3.67e+08 s-1
ISC rate = 2.57e+06 s-1

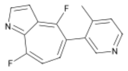

Triplet energy = 43.1 kcal/mol
Absorption maximum = 381 nm
Fluorescence rate = 4.18e+07 s-1
ISC rate = 4.47e+05 s-1

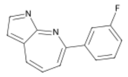

Triplet energy = 33.6 kcal/mol
Absorption maximum = 438 nm
Fluorescence rate = 2.71e+07 s-1
ISC rate = 2.91e+06 s-1

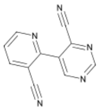

Triplet energy = 70.9 kcal/mol
Absorption maximum = 189 nm
Fluorescence rate = 1.48e+07 s-1
ISC rate = 4.91e+07 s-1

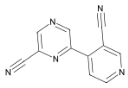

Triplet energy = 72.4 kcal/mol
Absorption maximum = 191 nm
Fluorescence rate = 1.82e+07 s-1
ISC rate = 1.26e+06 s-1

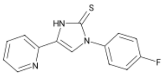

Triplet energy = 59.8 kcal/mol
Absorption maximum = 279 nm
Fluorescence rate = 1.06e+07 s-1
ISC rate = 1.11e+05 s-1

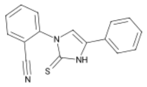

Triplet energy = 67.1 kcal/mol
Absorption maximum = 264 nm
Fluorescence rate = 2.70e+07 s-1
ISC rate = 1.69e+08 s-1

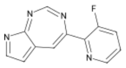

Triplet energy = 30.8 kcal/mol
Absorption maximum = 463 nm
Fluorescence rate = 4.09e+07 s-1
ISC rate = 5.69e+05 s-1

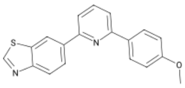

Triplet energy = 64.7 kcal/mol
Absorption maximum = 266 nm
Fluorescence rate = 9.88e+08 s-1
ISC rate = 5.61e+05 s-1

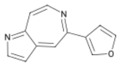

Triplet energy = 34.4 kcal/mol
Absorption maximum = 460 nm
Fluorescence rate = 4.62e+07 s-1
ISC rate = 1.89e+04 s-1

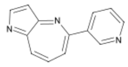

Triplet energy = 35.3 kcal/mol
Absorption maximum = 424 nm
Fluorescence rate = 1.09e+07 s-1
ISC rate = 2.57e+05 s-1

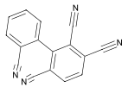

Triplet energy = 66.1 kcal/mol
Absorption maximum = 204 nm
Fluorescence rate = 2.10e+08 s-1
ISC rate = 2.16e+05 s-1

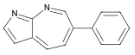

Triplet energy = 34.1 kcal/mol
Absorption maximum = 431 nm
Fluorescence rate = 3.84e+07 s-1
ISC rate = 1.08e+07 s-1

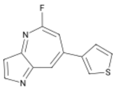

Triplet energy = 33.8 kcal/mol
Absorption maximum = 424 nm
Fluorescence rate = 1.38e+07 s-1
ISC rate = 1.32e+05 s-1

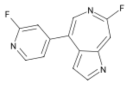

Triplet energy = 34.3 kcal/mol
Absorption maximum = 442 nm
Fluorescence rate = 3.23e+07 s-1
ISC rate = 5.24e+03 s-1

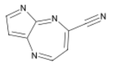

Triplet energy = 31.2 kcal/mol
Absorption maximum = 449 nm
Fluorescence rate = 6.59e+06 s-1
ISC rate = 2.96e+04 s-1

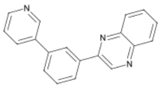

Triplet energy = 57.1 kcal/mol
Absorption maximum = 294 nm
Fluorescence rate = 6.47e+07 s-1
ISC rate = 5.27e+10 s-1

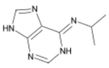

Triplet energy = 63.2 kcal/mol
Absorption maximum = 234 nm
Fluorescence rate = 3.95e+08 s-1
ISC rate = 1.30e+07 s-1

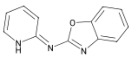

Triplet energy = 57.6 kcal/mol
Absorption maximum = 315 nm
Fluorescence rate = 7.39e+08 s-1
ISC rate = 3.73e+05 s-1

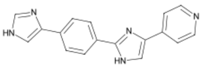

Triplet energy = 60.8 kcal/mol
Absorption maximum = 290 nm
Fluorescence rate = 1.23e+09 s-1
ISC rate = 6.15e+04 s-1

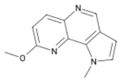

Triplet energy = 69.1 kcal/mol
Absorption maximum = 280 nm
Fluorescence rate = 5.52e+08 s-1
ISC rate = 3.94e+06 s-1

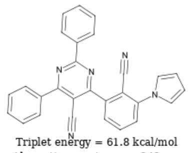

Triplet energy = 61.8 kcal/mol
Absorption maximum = 249 nm
Fluorescence rate = 4.07e+05 s-1
ISC rate = 3.22e+03 s-1

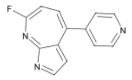

Triplet energy = 28.9 kcal/mol
Absorption maximum = 460 nm
Fluorescence rate = 2.64e+06 s-1
ISC rate = 3.87e+05 s-1

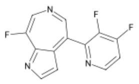

Triplet energy = 34.8 kcal/mol
Absorption maximum = 434 nm
Fluorescence rate = 3.29e+06 s-1
ISC rate = 1.79e+05 s-1

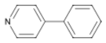

Triplet energy = 71.0 kcal/mol
Absorption maximum = 174 nm
Fluorescence rate = 7.61e+06 s-1
ISC rate = 1.05e+06 s-1

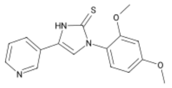

Triplet energy = 56.9 kcal/mol
Absorption maximum = 276 nm
Fluorescence rate = 6.72e+06 s-1
ISC rate = 3.13e+09 s-1

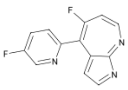

Triplet energy = 31.5 kcal/mol
Absorption maximum = 464 nm
Fluorescence rate = 1.42e+06 s-1
ISC rate = 2.05e+06 s-1

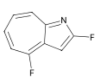

Triplet energy = 41.9 kcal/mol
Absorption maximum = 372 nm
Fluorescence rate = 4.85e+06 s-1
ISC rate = 3.39e+04 s-1

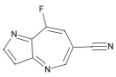

Triplet energy = 31.3 kcal/mol
Absorption maximum = 446 nm
Fluorescence rate = 1.88e+06 s-1
ISC rate = 4.59e+05 s-1

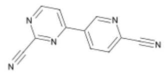

Triplet energy = 65.1 kcal/mol
Absorption maximum = 250 nm
Fluorescence rate = 2.10e+06 s-1
ISC rate = 2.94e+08 s-1

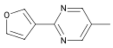

Triplet energy = 69.0 kcal/mol
Absorption maximum = 225 nm
Fluorescence rate = 3.60e+06 s-1
ISC rate = 2.15e+08 s-1

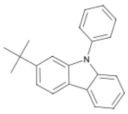

Triplet energy = 70.4 kcal/mol
Absorption maximum = 176 nm
Fluorescence rate = 3.77e+07 s-1
ISC rate = 3.72e+06 s-1

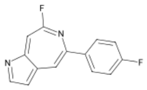

Triplet energy = 34.5 kcal/mol
Absorption maximum = 460 nm
Fluorescence rate = 1.08e+07 s-1
ISC rate = 1.92e+04 s-1

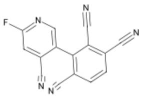

Triplet energy = 65.6 kcal/mol
Absorption maximum = 194 nm
Fluorescence rate = 1.29e+07 s-1
ISC rate = 8.34e+05 s-1

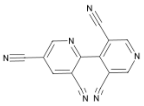

Triplet energy = 66.8 kcal/mol
Absorption maximum = 196 nm
Fluorescence rate = 4.96e+06 s-1
ISC rate = 8.96e+02 s-1

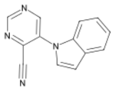

Triplet energy = 64.4 kcal/mol
Absorption maximum = 173 nm
Fluorescence rate = 1.89e+05 s-1
ISC rate = 2.06e+05 s-1

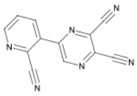

Triplet energy = 61.1 kcal/mol
Absorption maximum = 261 nm
Fluorescence rate = 8.64e+06 s-1
ISC rate = 1.20e+08 s-1

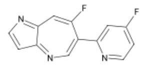

Triplet energy = 32.9 kcal/mol
Absorption maximum = 455 nm
Fluorescence rate = 1.26e+06 s-1
ISC rate = 4.09e+05 s-1

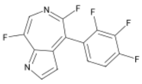

Triplet energy = 33.0 kcal/mol
Absorption maximum = 437 nm
Fluorescence rate = 4.91e+06 s-1
ISC rate = 3.23e+06 s-1

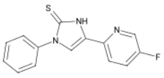

Triplet energy = 55.2 kcal/mol
Absorption maximum = 290 nm
Fluorescence rate = 4.60e+05 s-1
ISC rate = 2.10e+10 s-1

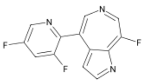

Triplet energy = 35.1 kcal/mol
Absorption maximum = 433 nm
Fluorescence rate = 3.01e+06 s-1
ISC rate = 2.74e+06 s-1

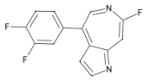

Triplet energy = 35.0 kcal/mol
Absorption maximum = 444 nm
Fluorescence rate = 8.60e+06 s-1
ISC rate = 3.92e+05 s-1

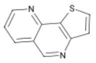

Triplet energy = 62.7 kcal/mol
Absorption maximum = 225 nm
Fluorescence rate = 1.68e+07 s-1
ISC rate = 5.90e+05 s-1

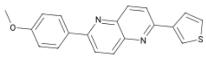

Triplet energy = 57.2 kcal/mol
Absorption maximum = 312 nm
Fluorescence rate = 1.51e+08 s-1
ISC rate = 5.18e+06 s-1

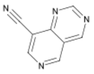

Triplet energy = 58.3 kcal/mol
Absorption maximum = 246 nm
Fluorescence rate = 7.86e+05 s-1
ISC rate = 1.36e+08 s-1

Figure S16: Molecules computed with the automated workflow.

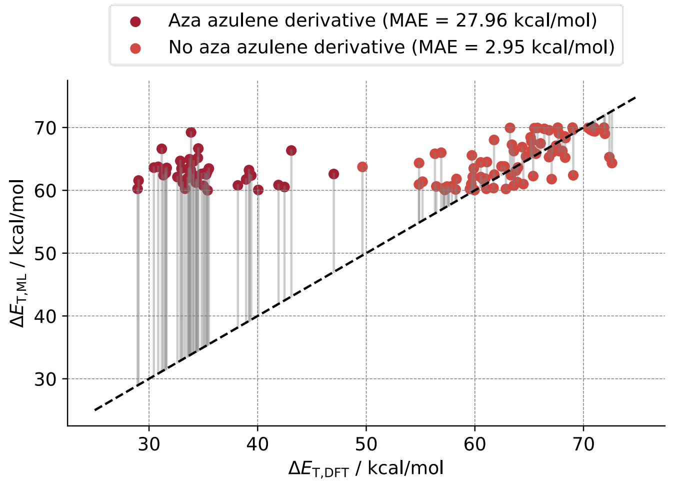

Figure S17: Scatter plot of triplet energies obtained with the ML model and by DFT. The classification between aza azuelene derivatives is indicated by the color and the MAE for the two sets are given separately.

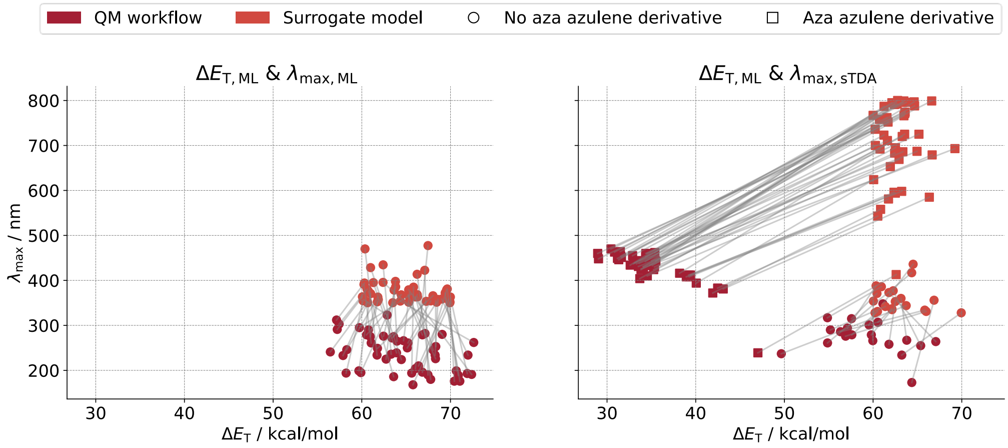

Figure S18: Scatter plot of predicted properties by the surrogate models and properties obtained with the QM screening workflow. The left plot shows the molecules for which λ_{max} was obtained with the ML proxy in the generative modelling. The properties predicted by the ML models (ΔE_T, $\lambda_{max\text{-}ML}$) as well as the properties obtained with the QM workflow are shown. The two sets of properties are connected with a gray line for each molecule indicating the difference in prediction by the surrogate model and the QM workflow. The right plot shows the same analysis for the predicted values using the ML model to predict ΔE_T and $\lambda_{max\text{-}sTDA}$ obtaind through the semi-empirical calculation.

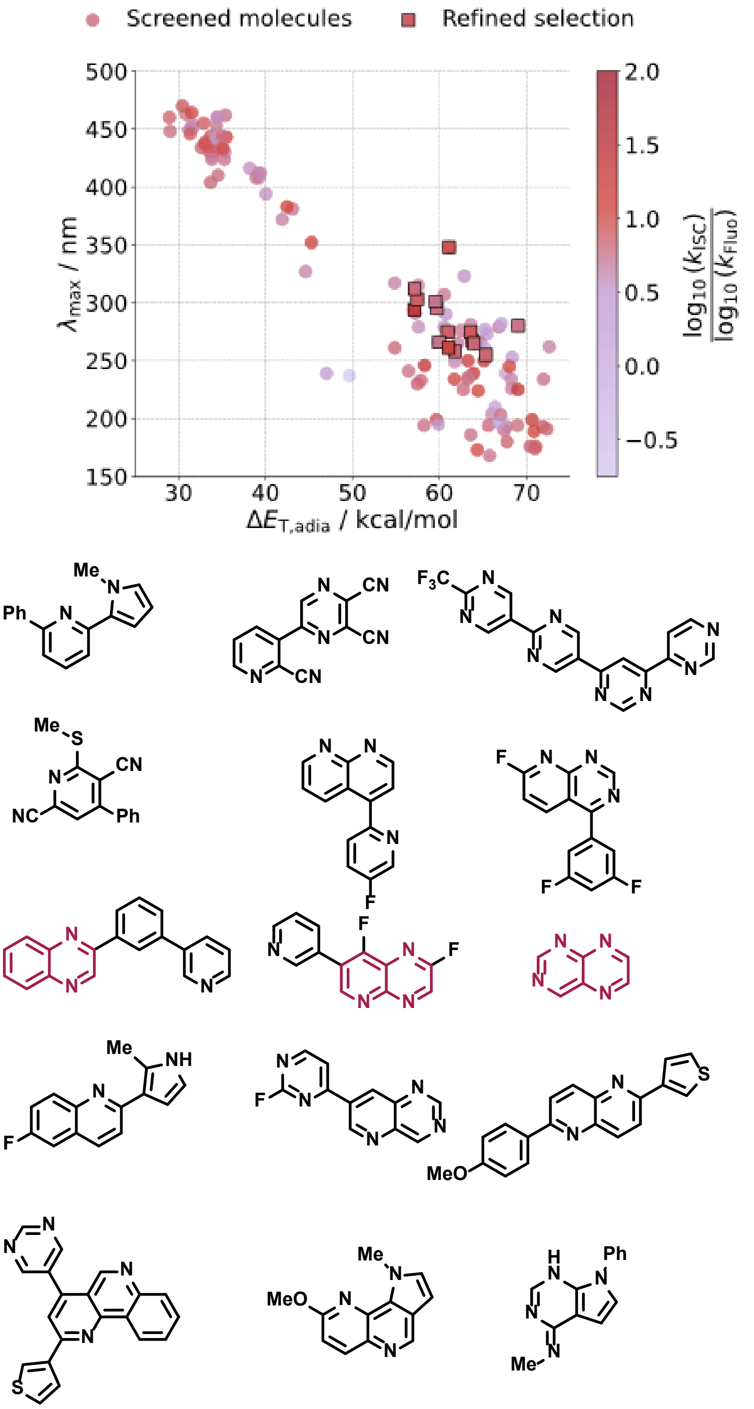

Figure S19: Computed photophysical properties of promising candidates. Molecules with a balanced set of properties are displayed as squares. Below are the structures of the refined selection (15 molecules). Purple substructure indicates the bicyclic (hetero)arene pyrazine derivatives that served as the core structure for the experimental investigations.

3. LOCAL EXPLORATION

3.1. Virtual library

A virtual library of PC candidates was created by combining six diamines with three diketones (**Figure S20**). The resulting 18 candidate structures were subjected to the automated computational workflow to obtain their photophysical properties (**Figure S21**).

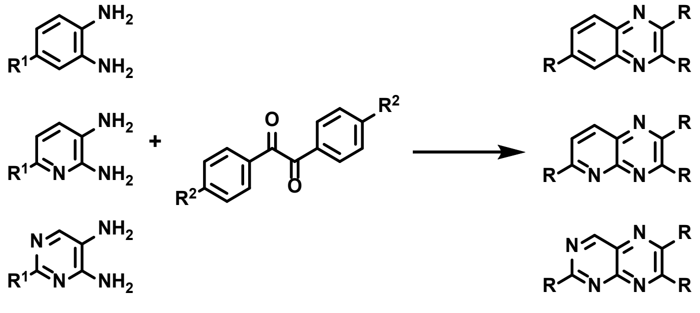

Figure S20: Scheme for creation of the local space based on bicyclic pyrazine derivatives.

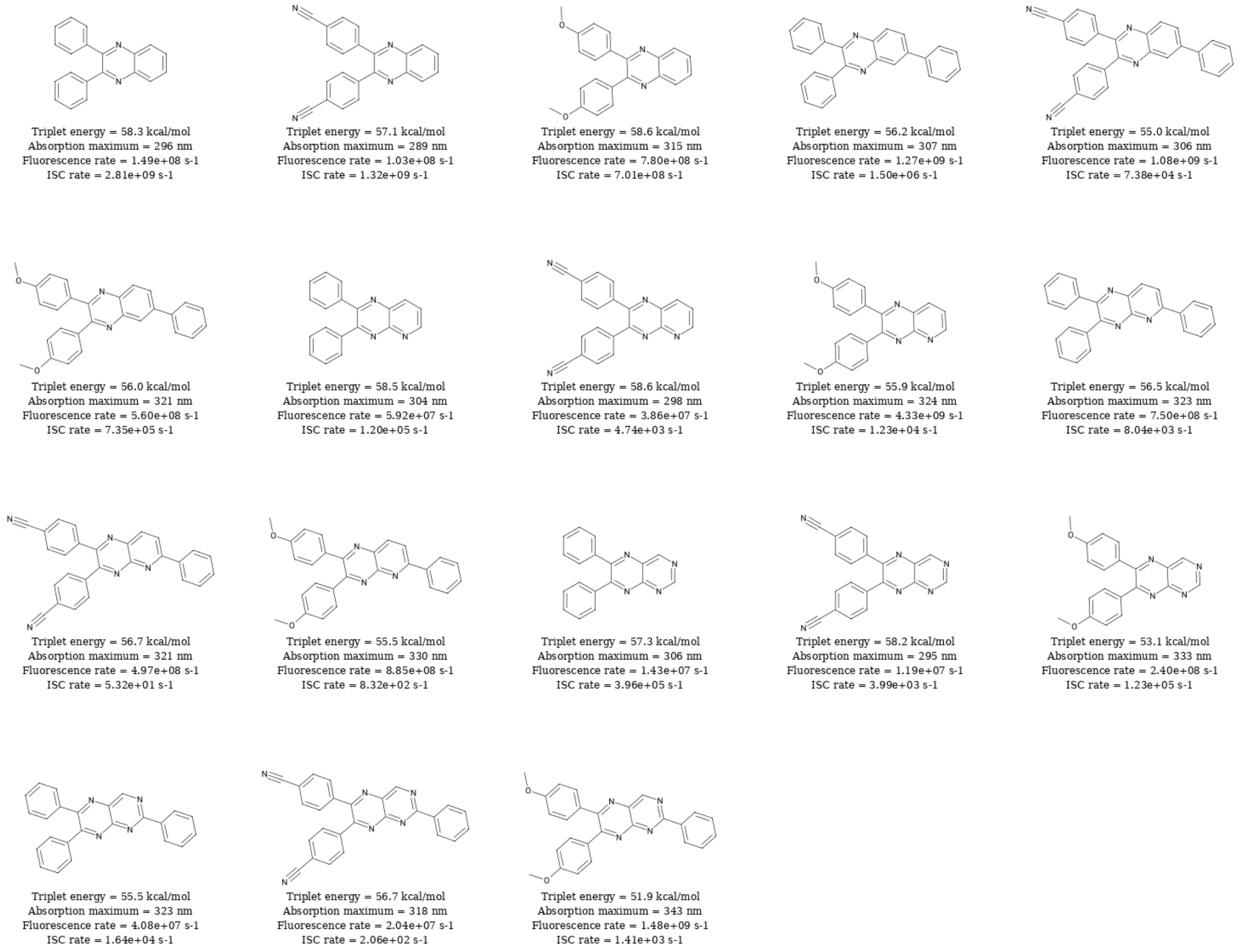

Figure S21: Computed properties for the local space.

Table S8: Comparison of computed absorption data and experimental values for PC1-4. I_{405} is the relative intensity at 405 nm divided by the intensity at λ_{max}.

PC	$\lambda_{max\text{-}QM}$ / nm	$I_{405\text{-}QM}$	$\lambda_{max\text{-}exp}$ / nm	$I_{405\text{-}exp}$
PC1	315	$1.3 \cdot 10^{-5}$	362.6	0.045
PC2	304	$1.5 \cdot 10^{-7}$	342.4	0.016
PC3	306	$1.5 \cdot 10^{-6}$	346.2	0.062
PC4	296	$2.8 \cdot 10^{-8}$	337.5	0.003

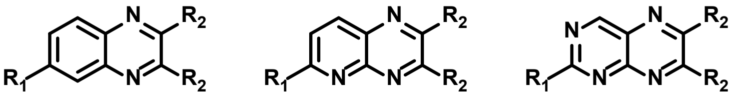

Qx **PyrPyr** **Pt**

Table S9: Predicted values ΔE_T, $\lambda_{max\text{-}ML}$, $\lambda_{max\text{-}sTDA}$, O_{FMO}, $\theta_{conjugation}$ obtained with the proxies used for the generative modelling for the candidates in the local space. The values in brackets indicate the transformed scores. The total score computed as shown in **Table S5** and **Table S6** is given.

Scaffold	R_1	R_2	ΔE_T /kcal mol^{-1}	$\lambda_{max\text{-}ML}$ / nm	$\lambda_{max\text{-}sTDA}$ / nm	O_{FMO}	$\theta_{conjugation}$	Total score (as in Table S5)	Total score (Table S6)
Qx	H	Ph	56.7 (0.87)	347 (0.87)	303 (0.00)	0.58 (0.32)	1.00 (1.00)	0.91	0.72
	H	*p*-CN-Ph	53.2 (0.12)	340 (0.76)	302 (0.00)	0.59 (0.31)	1.00 (1.00)	0.37	0.35
	H	*p*-MeO-Ph	56.2 (0.79)	333 (0.59)	320 (0.00)	0.58 (0.32)	0.93 (1.00)	0.78	0.64
	Ph	Ph	55.9 (0.74)	359 (0.97)	313 (0.00)	0.58 (0.33)	1.00 (1.00)	0.87	0.7
	Ph	*p*-CN-Ph	52.7 (0.07)	352 (0.93)	316 (0.00)	0.58 (0.32)	1.00 (1.00)	0.3	0.31
	Ph	*p*-MeO-Ph	55.8 (0.72)	351 (0.92)	326 (0.00)	0.57 (0.34)	0.94 (1.00)	0.85	0.69
PyrPyr	H	Ph	57.4 (0.94)	348 (0.89)	391 (1.00)	0.57 (0.34)	1.00 (1.00)	0.94	0.75
	H	*p*-CN-Ph	55.3 (0.58)	365 (0.98)	395 (1.00)	0.57 (0.35)	1.00 (1.00)	0.79	0.66

	H	*p*-MeO-Ph	56.0 (0.76)	357 (0.96)	329 (0.01)	0.57 (0.35)	0.93 (1.00)	0.88	0.72
	Ph	Ph	53.6 (0.16)	368 (0.99)	322 (0.00)	0.56 (0.37)	1.00 (1.00)	0.45	0.43
	Ph	*p*-CN-Ph	51.3 (0.01)	389 (1.00)	324 (0.00)	0.56 (0.36)	1.00 (1.00)	0.16	0.2
	Ph	*p*-MeO-Ph	51.8 (0.03)	385 (1.00)	341 (0.11)	0.55 (0.38)	0.94 (1.00)	0.21	0.24
Pt	H	Ph	55.8 (0.72)	363 (0.98)	435 (1.00)	0.56 (0.36)	1.00 (1.00)	0.86	0.71
	H	*p*-CN-Ph	53.8 (0.19)	365 (0.98)	425 (1.00)	0.55 (0.38)	1.00 (1.00)	0.49	0.46
	H	*p*-MeO-Ph	54.7 (0.40)	372 (0.99)	422 (1.00)	0.56 (0.37)	0.93 (1.00)	0.68	0.59
	Ph	Ph	56.2 (0.80)	348 (0.88)	444 1.00)	0.55 (0.38)	1.00 (1.00)	0.88	0.73
	Ph	*p*-CN-Ph	54.2 (0.28)	343 (0.81)	429 (1.00)	0.55 (0.40)	1.00 (1.00)	0.55	0.51
	Ph	*p*-MeO-Ph	54.2 (0.29)	353 (0.94)	430 (1.00)	0.55 (0.38)	0.94 (1.00)	0.58	0.53

4. GENERAL EXPERIMENTAL

4.1. Glassware, Solvents and Reagents

All reactions were conducted under an inert atmosphere of argon using Schlenk manifold techniques unless stated otherwise. All glassware and Teflon-coated magnetic stir bars were dried in an oven at 80 °C prior to use. All anhydrous solvents were commercially supplied and stored over 3 Å mol. sieves or dried using an activated alumina column drying system (MeCN, CH_2Cl_2, hexane, toluene, THF, Et_2O, DMF, MeOH). Reagents were purchased from commercial sources and used as received.

4.2. Photochemical set-up and light sources

Photochemical reactions were performed in a Hepatochem EvoluChemTM PhotoRedOx Box Duo device and irradiated with two EvoluChem™ HCK1012-02-012 LEDs (18 W, λ_{max} = 405 nm, **Figure S22**). With the internal fan, the reaction temperature was determined to be between 30 °C and 33 °C.

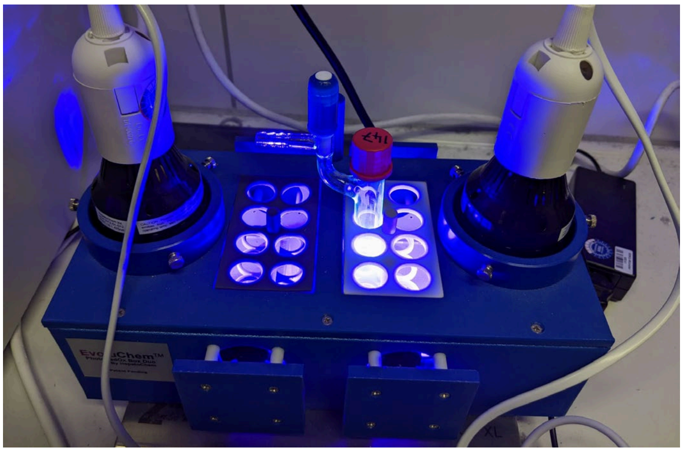

Figure S22: Experimental set-up for photochemical reactions.

4.3. Chromatography and Data Analysis

Thin layer chromatography (TLC) was performed to monitor reactions when practical using Merck silica gel 60 F_{254} aluminum plates and visualised under UV light, or by staining with aqueous basic potassium permanganate followed by heating. **Flash column chromatography** (FCC) was carried out using Acros Organics silica gel (35–70 mesh). **NMR spectra** were recorded on a Bruker Avance II 400, Agilent DD2 500 or DD2 600 spectrometers. All spectral data was acquired at 295 K. Deuterated solvents were purchased from Eurisotop ($CDCl_3$, deuteration > 99.8%, CD_2Cl_2, deuteration > 99.8%). Chemical shifts (δ) are reported in parts per million (ppm) and referenced to $CDCl_3$ (^1H: 7.26 ppm; ^{13}C: 77.16 ppm) or CD_2Cl_2 (^1H: 5.32 ppm; ^{13}C: 53.84 ppm). Coupling constants (J) are given in Hertz (Hz) and refer to corresponding multiplicities (s = singlet, d = doublet, t = triplet, q = quartet, quin = quintet, hex = hextet, h = heptet, m = multiplet, app = apparent, br. = broad signal, dd = doublet of doublets, etc.). The ^1H NMR spectra are reported as follows: chemical shift (multiplicity, coupling constants, number of protons). NMR assignments were made according to spin systems, using two-dimensional NMR spectroscopy (COSY, HSQC, HMBC) to assist the characterisation. NMR yields were determined by ^1H NMR analysis using dibromomethane as an internal standard. The *d.r.* and *r.r.* values were determined by ^1H NMR analysis of the crude reaction mixture. When only a single regioisomer was detected, no *r.r.* is given. >20:1 *d.r.* indicates when only a single diastereomer could be detected. **High resolution mass spectra (HRMS)** were recorded using electrospray ionisation (ESI) on a Bruker Daltonics, MicroToF spectrometer and calibrated using formate ion clusters.

4.4. Naming of Compounds

Compound names are those generated by ChemDraw Professional 23.0 software (PerkinElmer), following the IUPAC nomenclature.

5. EXPERIMENTAL DATA

5.1. Synthesizing of the photocatalysts

2,3-bis(4-methoxyphenyl)quinoxaline (PC 1)

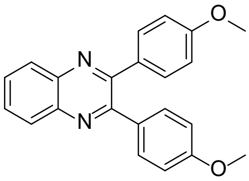

PC 1

The product was synthesized following an adapted literature procedure.[44] To an 10 mL round bottom flask equipped with a Teflon-coated magnetic stir bar was added benzene-1,2-diamine (108 mg, 1.00 mmol, 1.00 equiv.), 1,2-bis(4-methoxyphenyl)ethane-1,2-dione (270 mg, 1.0 mmol, 1.00 equiv), and MeOH (5 mL). $ZrCl_4$ (12 mg, 0.05 mmol, 5 mol%) was added. The reaction mixture was stirred at room temperature for 30 min. After this time, water (5 mL) and CH_2Cl_2 (5 mL) was added. The layer were separated and the aqueous layer was extracted with CH_2Cl_2 (2 x 5 mL). The combined organic layers were washed with sat. aq. NaCl (15 ml per 1 mmol), dried over MgSO4 and the solvent was removed under reduced pressure to afford the product as a beige solid (220 mg, 0.642 mmol, 64%).

NMR Spectroscopy (*see spectra*):

^{1}H NMR (400 MHz, $CDCl_3$): δ_H; 8.13 (ddd, J = 6.4, 3.4, 1.3 Hz, 2H), 7.73 (ddd, J = 6.4, 3.4, 1.2 Hz, 2H), 7.54 – 7.46 (m, 4H), 6.92 – 6.84 (m, 4H), 3.84 (s, 6H),

^{13}C NMR (101 MHz, $CDCl_3$): δ_C; 160.3, 153.2, 141.2, 131.9, 131.4, 129.7, 129.2, 113.9, 55.5.

HRMS (ESI$^+$): m/z calc'd for $C_{22}H_{18}N_2O_2Na$: [M+Na]$^+$: 365.12605, found: 365.12614.

2,3-diphenylpyrido[2,3-b]pyrazine (PC 2)

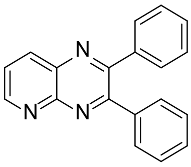

PC 2

The product was synthesized following an adapted literature procedure.[45] To an 10 mL round bottom flask equipped with a Teflon-coated magnetic stir bar was added pyridine-2,3-diamine (119 mg, 1.10 mmol, 1.10 equiv.), benzil (210 mg, 1.0 mmol, 1.00 equiv), and MeOH (600 µL). Sulfamic acid (5 mg, 0.05 mmol, 5 mol%) was added. The reaction mixture was stirred at room temperature for 1 h. The solvent was removed in vacuo and the product was purified using column chromatography on silica gel (60:40 → 50:50 pentane:EtOAc).

TLC: R_f = 0.18 (60:40 pentane:EtOAc).

NMR Spectroscopy (*see spectra*):

^{1}H NMR (400 MHz, $CDCl_3$): δ_H; 9.17 (dt, J = 4.1, 1.6 Hz, 1H), 8.52 (dt, J = 8.3, 1.5 Hz, 1H), 7.76 – 7.67 (m, 1H), 7.67 – 7.60 (m, 2H), 7.59 – 7.51 (m, 2H), 7.45 – 7.29 (m, 6H);

^{13}C NMR (101 MHz, $CDCl_3$): δ_C; 156.5, 154.9, 154.2, 150.0, 138.7, 138.2, 138.2, 136.3, 130.4, 130.0, 129.6, 129.4, 128.6, 128.3, 125.3.

HRMS (ESI$^+$): *m*/*z* calc'd for $C_{19}H_{13}N_3$ Na [M+Na]$^+$: 306.10017, found: 306.10004.

6,7-diphenylpteridine (PC 3)

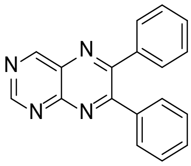

PC 3

The product was synthesized following an adapted literature procedure.[46] To an 10 mL round bottom flask equipped with a Teflon-coated magnetic stir bar was added pyrimidine-4,5-diamine (264 mg, 2.40 mmol, 1.20 equiv.), benzil (420 mg, 2.0 mmol, 1.00 equiv), and H_2O (4 mL). Bi(OTf)$_3$ (131 mg, 0.2 mmol, 10 mol%) was added. The reaction mixture was stirred at 60 °C for 16 h. After this time, EtOAc (5 mL) was added. The layers were separated and the aqueous layer was extracted with EtOAc (2 x 5 mL). The combined organic layers were dried over $MgSO_4$, the solvent was removed under reduced pressure. The product was purified by flash column chromatography (70:30 → 60:40 pentane:EtOAc) on silica gel to yield the compound as a beige solid (220 mg, 0.642 mmol, 64%).

TLC: R_f = 0.35 (60:40 pentane:EtOAc).

NMR Spectroscopy (*see spectra*):

^{1}H NMR (400 MHz, $CDCl_3$): δ_H; 9.74 (s, 1H), 9.57 (s, 1H), 7.67 – 7.61 (m, 2H), 7.59 – 7.50 (m, 2H), 7.49 – 7.31 (m, 6H);

^{13}C NMR (101 MHz, $CDCl_3$): δ_C; 162.8, 161.2, 158.9, 156.7, 152.4, 137.9, 137.5, 132.8, 130.6, 130.5, 130.1, 129.9, 128.7, 128.5.

--

HRMS (ESI$^+$): *m*/*z* calc'd for $C_{18}H_{12}N_4$Na [M+Na]$^+$: 307.09542, found: 307.09509.

2,3-diphenylquinoxaline (PC 4)

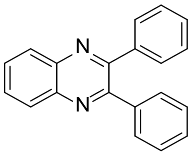

The product was synthesized following an adapted literature procedure.[45] To an 10 mL round bottom flask equipped with a Teflon-coated magnetic stir bar was added pyridine-2,3-diamine (119 mg, 1.10 mmol, 1.10 equiv.), benzil (210 mg, 1.0 mmol, 1.00 equiv), and MeOH (600 µL). Sulfamic acid (5 mg, 0.05 mmol, 5 mol%) was added. The reaction mixture was stirred at room temperature for 1 h. The solvent was removed in vacuo and the product was recrystallized from hot EtOH to yield the title compound as a white solid (252 mg, 0.891 mmol, 89 %).

NMR Spectroscopy (*see spectra*):

^{1}H NMR (400 MHz, $CDCl_3$): δ_H; 8.19 (ddd, *J* = 7.0, 3.7, 1.8 Hz, 2H), 7.82 – 7.72 (m, 2H), 7.53 (dd, *J* = 6.7,

2.1 Hz, 4H), 7.41 – 7.29 (m, 6H),

^{13}C NMR (101 MHz, $CDCl_3$): δ_C; 153.6, 141.4, 139.2, 130.1, 130.0, 129.3, 128.9, 128.4.

HRMS (ESI$^+$): *m*/*z* calc'd for $C_{20}H_{14}N_2Na$ [M+Na]$^+$: 305.10492, found: 305.10487.

5.2. Establishing the photocatalyst

5.2.1. Photoisomerization

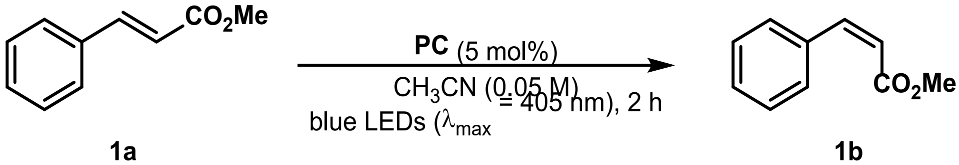

Table S10: Establishing the photocatalyst in the photoisomerization of methyl cinnamate

Entry	PC	1a:1b[a]
1	**PC1**	23:77
2	**PC2**	30:70
3	**PC3**	37:63
4	No **PC**	99:1

[a] relative ratio of **1a:1b** was determined by ^{1}H NMR analysis.

To an oven-dried 10 mL Schlenk tube equipped with a Teflon-coated magnetic stir bar was added **PC** (5 mol%) and methyl cinnamate **1a** (32.4 µL, 0.2 mmol, 1.0 equiv). The Schlenk tube was evacuated and backfilled with argon three times before, DCM (1 mL) were added under a positive argon pressure. The reaction mixture was stirred under irradiation with blue LEDs (18 W, λ_{max} = 405 nm) for 2 h. After this time, the solvent was removed under reduced pressure. The relative ratio of **1a:1b** was determined by ^{1}H NMR analysis.

5.2.2. [2+2]-Cycloaddition

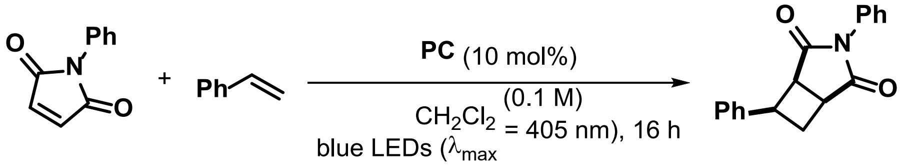

Table S11: Establishing the photocatalyst in the [2+2]-cycloaddition of phenylmaleimide (**2**) with styrene (**3**).

Entry	PC	% Yield[a]	*d.r.[a]*
1	**PC1**	80	2:1
2	**PC2**	68	1:1
3	**PC3**	46	1:1
4	No **PC**	<5	

[a] determined by ^{1}H NMR analysis using CH_2Br_2 as an internal standard.

For reference, Kokotos *et al.*, obtained **4** in 75% (^{1}H NMR) yield using thioxanthone (20 mol%) as the PC

(reaction performed in CH_2Cl_2 (0.1 M), 440 nm, 16h).[47]

Following a literature procedure to an oven-dried 10 mL Schlenk tube equipped with a Teflon-coated magnetic stir bar was added **PC** (10 mol%) and 1-phenyl-1H-pyrrole-2,5-dione **2** (34.6 mg, 0.2 mmol, 1.0 equiv). The Schlenk tube was evacuated and backfilled with argon three times before, DCM (2 mL) and styrene (46 µL, 0.2 mmol, 2.0 equiv) were added under a positive argon pressure. The reaction mixture was stirred under irradiation with blue LEDs (18 W, λ_{max} = 405 nm) for 16 h. After this time, the solvent was removed under reduced pressure and to the crude mixture CH_2Br_2 was added. The yield was determined using NMR.

For **Entry 1** the reaction was concentrated, and the product was purified by flash column chromatography (90:10 → 50:50 pentane:EtOAc) on silica gel to yield the diastereomer **4a** as white solid (26 mg, 0.094 mmol, 47%) and diastereomer **4b** as white solid (13 mg, 0.048 mmol, 24%).

***anti*-3,6-diphenyl-3-azabicyclo[3.2.0]heptane-2,4-dione (4a)**

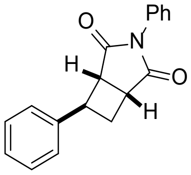

TLC: R_f = 0.48 (80:20 pentane:EtOAc).

NMR Spectroscopy (*see spectra*):

^{1}H NMR (400 MHz, $CDCl_3$): δ_H; 7.57 – 7.48 (m, 2H), 7.45 – 7.32 (m, 7H), 7.31 – 7.27 (m, 1H), 3.92 – 3.75 (m, 1H), 3.57 (dd, *J* = 6.6, 5.6 Hz, 1H), 3.49 (dddd, *J* = 10.6, 6.9, 3.9, 1.1 Hz, 1H), 2.96 – 2.76 (m, 2H),

^{13}C NMR (101 MHz, $CDCl_3$): δ_C; 178.7, 177.6, 142.2, 132.3, 129.4, 129.0, 128.8, 127.3, 126.6, 126.5, 46.7, 42.4, 36.1, 30.2.

HRMS (ESI$^+$): *m*/*z* calc'd for $C_{18}H_{15}NO_2Na$ [M+Na]$^+$: 300.09950, found: 300.09952.

***syn*-3,6-diphenyl-3-azabicyclo[3.2.0]heptane-2,4-dione (4b)**

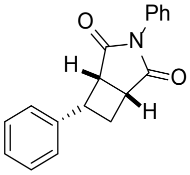

TLC: R_f = 0.29 (80:20 pentane:EtOAc).

NMR Spectroscopy (*see spectra*):

^{1}H NMR (400 MHz, $CDCl_3$): δ_H; 7.43 – 7.26 (m, 8H), 7.05 – 6.96 (m, 2H), 4.32 (td, *J* = 10.3, 7.0 Hz, 1H), 3.85 (dd, *J* = 10.5, 6.6 Hz, 1H), 3.49 (ddd, *J* = 10.7, 6.6, 4.5 Hz, 1H), 3.13 (dt, *J* = 13.5, 10.2 Hz, 1H), 2.76 (ddd, *J* = 13.7, 7.1, 4.4 Hz, 1H),

^{13}C NMR (101 MHz, $CDCl_3$): δ_C; 178.9, 175.5, 138.0, 132.1, 129.2, 128.8, 128.6, 127.7, 127.6, 126.4, 44.6, 40.0, 35.9, 27.6.

HRMS (ESI$^+$): *m*/*z* calc'd for $C_{18}H_{15}NO_2Na$ [M+Na]$^+$: 300.09950, found: 300.09952.

5.2.3. One pot aza-photocycloaddition

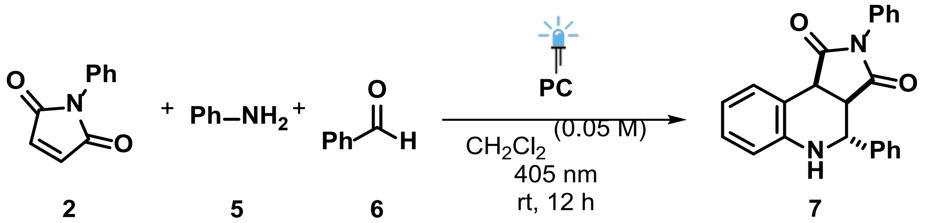

Table S12: Establishing the photocatalyst in the one pot aza-photocycloaddition.

Entry	PC (mol%)	% Yield[a]	*d.r.*
1	**PC1 (10 mol%)**	81	8:1
2	**PC1 (1 mol%)**	90	9:1
3	**PC2 (10 mol%)**	12	
4	**PC3 (10 mol%)**	9	
45	Thioxanthone **(10 mol%)**	56	9:1
56	4CzIPN **(10 mol%)**	90	5:1
67	$[Ir(dF(CF_3)ppy)_2(dtbbppy)]PF_6$ **(1 mol%)**	59	8:1

[a] determined by ^1H NMR analysis using CH_2Br_2 as an internal standard.

Following a literature procedure to an oven-dried 10 mL Schlenk tube equipped with a Teflon-coated magnetic stir bar was added **PC** and 1-phenyl-1H-pyrrole-2,5-dione **2** (34.6 mg, 0.2 mmol, 1.0 equiv). The Schlenk tube was evacuated and backfilled with argon three times before, CH_2Cl_2 (4 mL), aniline (22 µL, 0.24 mmol, 1.2 equiv) and benzaldehyde (30 µL, 0.3 mmol, 1.5 equiv) were added under a positive argon pressure. The reaction mixture was stirred under irradiation with blue LEDs (18 W, λ_{max} = 405 nm) for 12 h. After this time, the solvent was removed under reduced pressure and to the crude mixture CH_2Br_2 was added. The yield was determined using NMR.

For **Entry 1** the reaction was concentrated, and the product was purified by flash column chromatography (90:10 → 80:20 pentane:EtOAc) on silica gel to yield the diastereomer **7a** as white solid (48 mg, 0.14 mmol, 68%). Diastereomer **7b** could not be isolated.

***anti*- 2,4-diphenyl-3a,4,5,9b-tetrahydro-1H-pyrrolo[3,4-c]quinoline-1,3(2H)-dione (7a)**

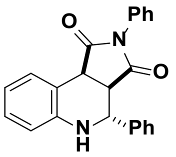

TLC: R_f = 0.35 (60:40 pentane:EtOAc).

NMR Spectroscopy (*see spectra*):

^{1}H NMR (400 MHz, CD_2Cl_2): δ_H; 7.60 (d, *J* = 7.7 Hz, 1H), 7.48 (t, *J* = 7.5 Hz, 2H), 7.44 – 7.31 (m, 6H), 7.28 (d, *J* = 7.7 Hz, 2H), 7.14 (t, *J* = 7.7 Hz, 1H), 6.86 (t, *J* = 7.5 Hz, 1H), 6.68 (d, *J* = 8.0 Hz, 1H), 4.67 (d, *J* = 5.7 Hz, 1H), 4.36 (s, 1H), 4.17 (d, *J* = 8.7 Hz, 1H), 3.65 (dd, *J* = 8.6, 5.7 Hz, 1H),

^{13}C NMR (101 MHz, CD_2Cl_2): δ_C; 176.0, 175.9, 144.5, 141.6, 132.5, 130.6, 129.4, 129.1, 129.0, 129.0, 128.5, 127.6, 126.9, 119.6, 115.8, 115.8, 55.6, 48.3, 41.4.

HRMS (ESI$^+$): *m/z* calc'd for $C_{23}H_{18}N_2O_2Na$ [M+Na]$^+$: 377.12605, found: 377.12600.

Investigation of photostability of PC1

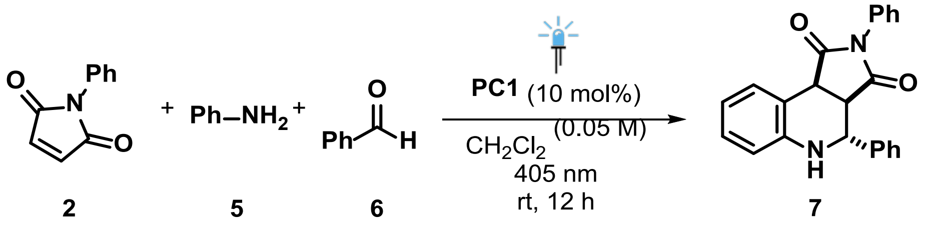

Table S13: Photostability experiment of **PC1** in the one pot aza-photocycloaddition.

Entry	PC (mol%)	% Yield[a]
1	**PC1** (10 mol%)	81
2	Recovered **PC1** from entry 1 (9 mol%)	80

[a] determined by ^{1}H NMR analysis using CH_2Br_2 as an internal standard.

Following the procedure described in section 5.2.3 an oven-dried 10 mL Schlenk tube equipped with a Teflon-coated magnetic stir bar was added **PC1** (6.8 mg, 10 mol%) and 1-phenyl-1H-pyrrole-2,5-dione **2** (34.6 mg, 0.2 mmol, 1.0 equiv). The Schlenk tube was evacuated and backfilled with argon three times before, CH_2Cl_2 (4 mL), aniline (22 µL, 0.24 mmol, 1.2 equiv) and benzaldehyde (30 µL, 0.3 mmol, 1.5 equiv) were added under a positive argon pressure. The reaction mixture was stirred under irradiation with blue LEDs (18 W, λ_{max} = 405 nm) for 12 h. After this time, the solvent was removed under reduced pressure and to the crude mixture CH_2Br_2 was added. The yield was determined using NMR.

For **Entry 2** the reaction mixture from **Entry 1** was concentrated, and the catalyst was recovered by flash column chromatography on silica gel to yield **PC1** as a beige solid (6.0 mg, 0.018 mmol, 90%). Using the recovered **PC1** for the same procedure yielded product **7** in 80% yield.

5.3. UV/vis Absorption Spectroscopy

UV/vis absorption spectra were recorded on a Jasco V-730 spectrophotometer, equipped with a temperature control unit at 25 °C. The samples were measured in Starna® fluorescence quartz cuvettes (type: 29-F, chamber volume = 1.400 mL, H × W × D = 48 mm × 12.5 mm × 12.5 mm, path length = 10 mm).

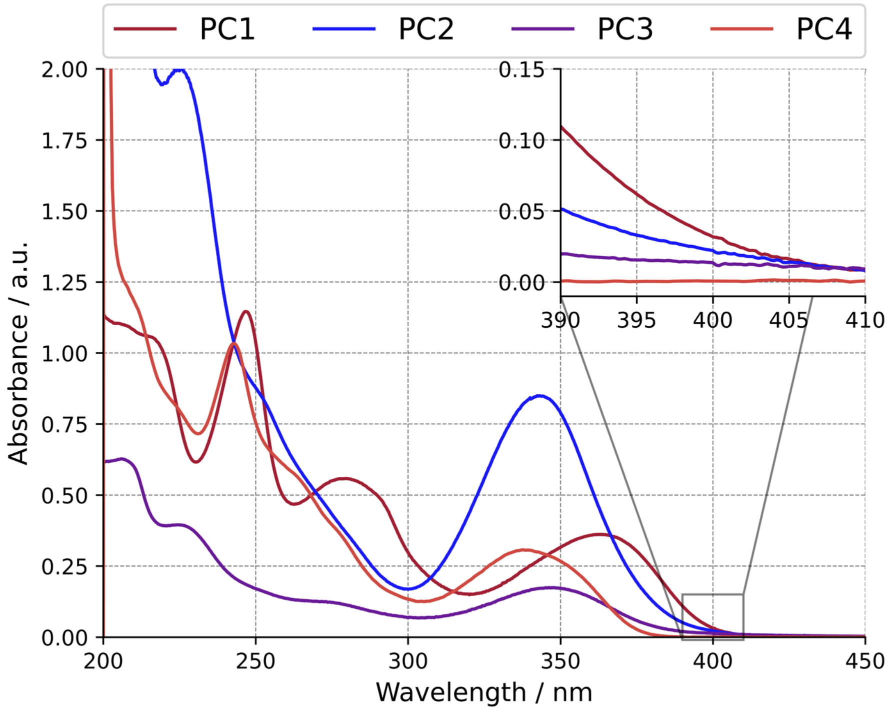

Figure S23: UV/vis absorption spectra of **PC1-4** in MeCN.

5.4. Cyclic Voltammetry

In order to determine the redox potentials of the reagents, cyclic voltammetry studies (CV) with a standard three electrode set up was conducted. The set up was equipped with a reference electrode (Ag/AgCl; 2 M LiCl solution in EtOH), a working electrode (3 mm glassy carbon disc electrode) and counter electrode (platinum wire) on a Metrohm µ-Stat-i 400s potentiostat (Metrohm, Steinhagen, Germany). For the measurements a solution of tetrabutylammonium hexafluorophosphate ($TBAPF_6$) (0.05 M) in MeCN (dry, LC-MS grade) was prepared as electrolyte. Prior to the measurement the solution was degassed inside the setup, while bubbling argon through it. First a blank CV and ferrocene was measured, followed by the first PC (0.05 mmol), which was dissolved in the previously prepared electrolyte solution (20 mL, 0.05 M). The process was repeated independently for all the PCs. In between the measurements all electrodes and the solvent bulb were washed with MeCN (LC-MS grade). No additional blank CV was performed. All CV studies were carried out under argon atmosphere at rt and with a scan rate of 0.1 V/s.

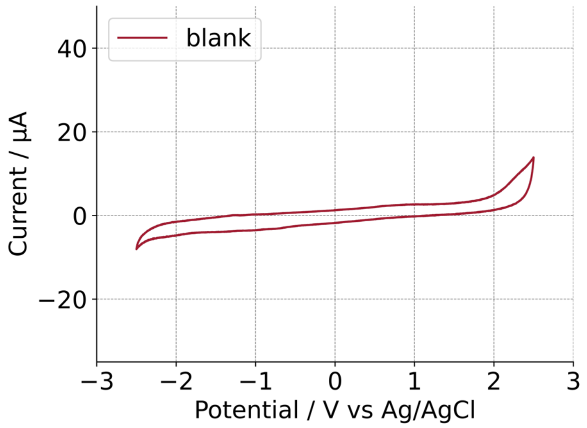

Figure S24: Cyclic voltammetry of electrolyte solution ($TBAPF_6$, 0.1 M), using a 2 mm glassy carbon disk working electrode, Pt sheet counter electrode and an Ag/AgCl (2 M LiCl in ethanol) reference electrode.

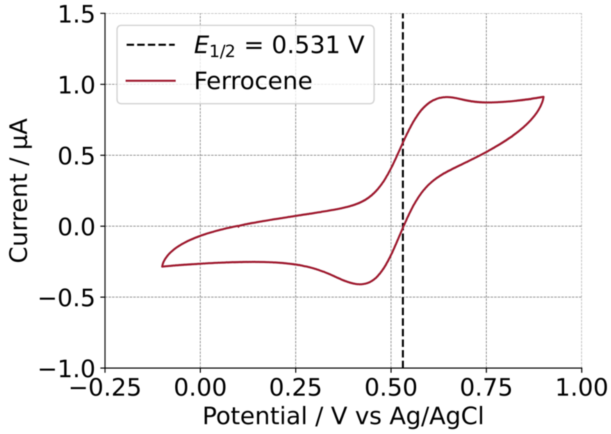

Figure S25: Cyclic voltammetry of Ferrocene in 0.1 M TBAPF6, using a 2 mm glassy carbon disk working electrode, Pt sheet counter electrode and an Ag/AgCl (2 M LiCl in ethanol) reference electrode.

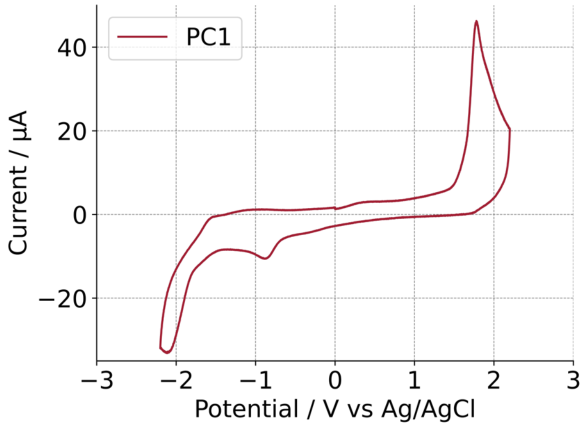

Figure S26: Cyclic voltammetry of PC1 in 0.1 M TBAPF6, using a 2 mm glassy carbon disk working electrode, Pt sheet counter electrode and an Ag/AgCl (2 M LiCl in ethanol) reference electrode.

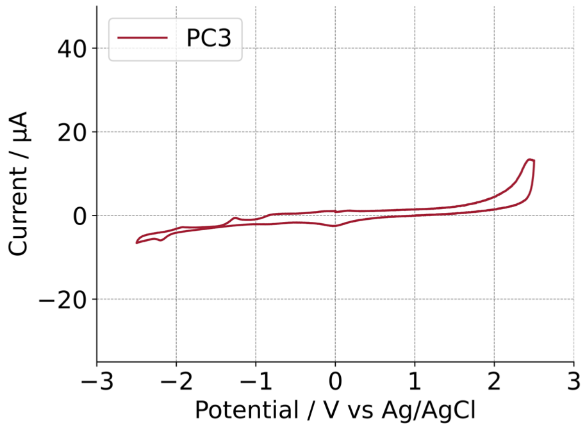

Figure S27: Cyclic voltammetry of PC3 in 0.1 M TBAPF6, using a 2 mm glassy carbon disk working electrode, Pt sheet counter electrode and an Ag/AgCl (2 M LiCl in ethanol) reference electrode.

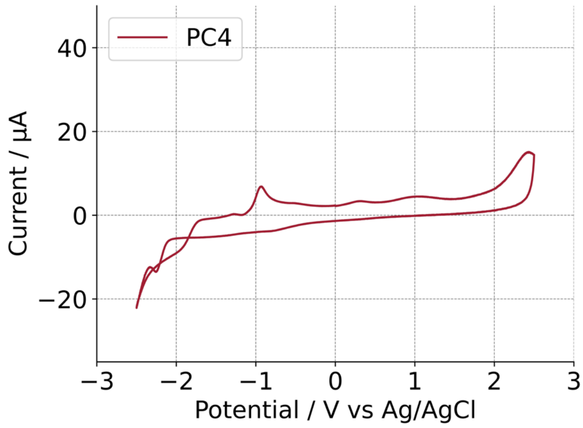

Figure S28: Cyclic voltammetry of PC4 in 0.1 M TBAPF6, using a 2 mm glassy carbon disk working electrode, Pt sheet counter electrode and an Ag/AgCl (2 M LiCl in ethanol) reference electrode.

6. SPECTROSCOPIC DATA

^{1}H NMR (400 MHz, $CDCl_3$) of **PC1** (*see procedure*)

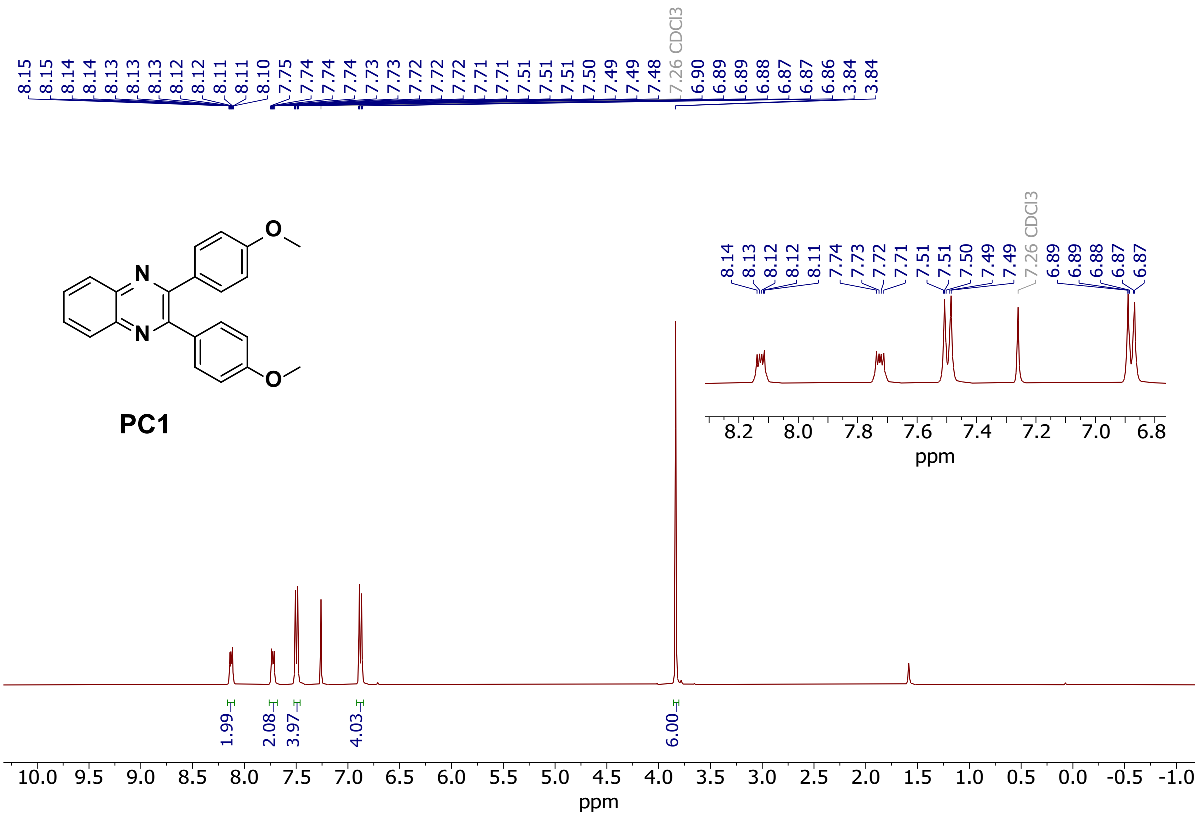

PC1

^{13}C NMR (101 MHz, $CDCl_3$) of **PC1**

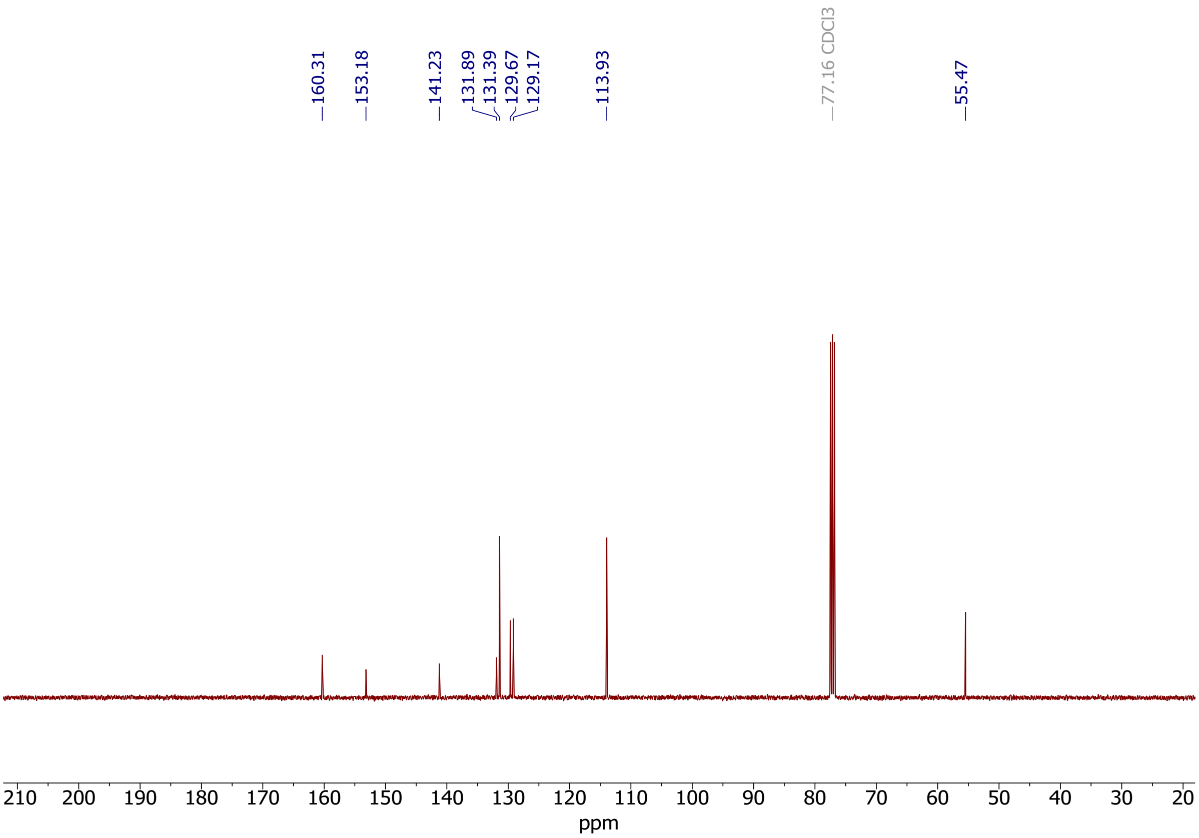

^{1}H NMR (400 MHz, $CDCl_3$) of **PC2** (*see procedure*)

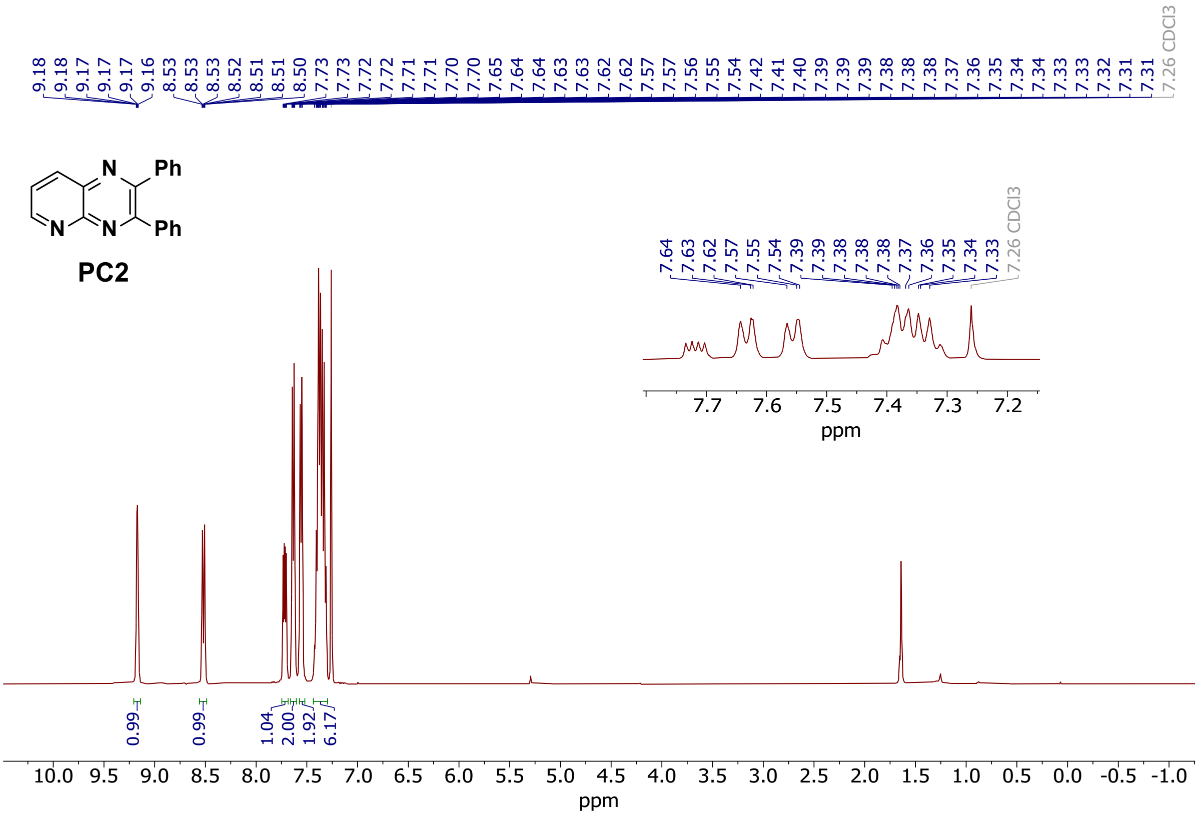

PC2

^{13}C NMR (101 MHz, $CDCl_3$) of **PC2**

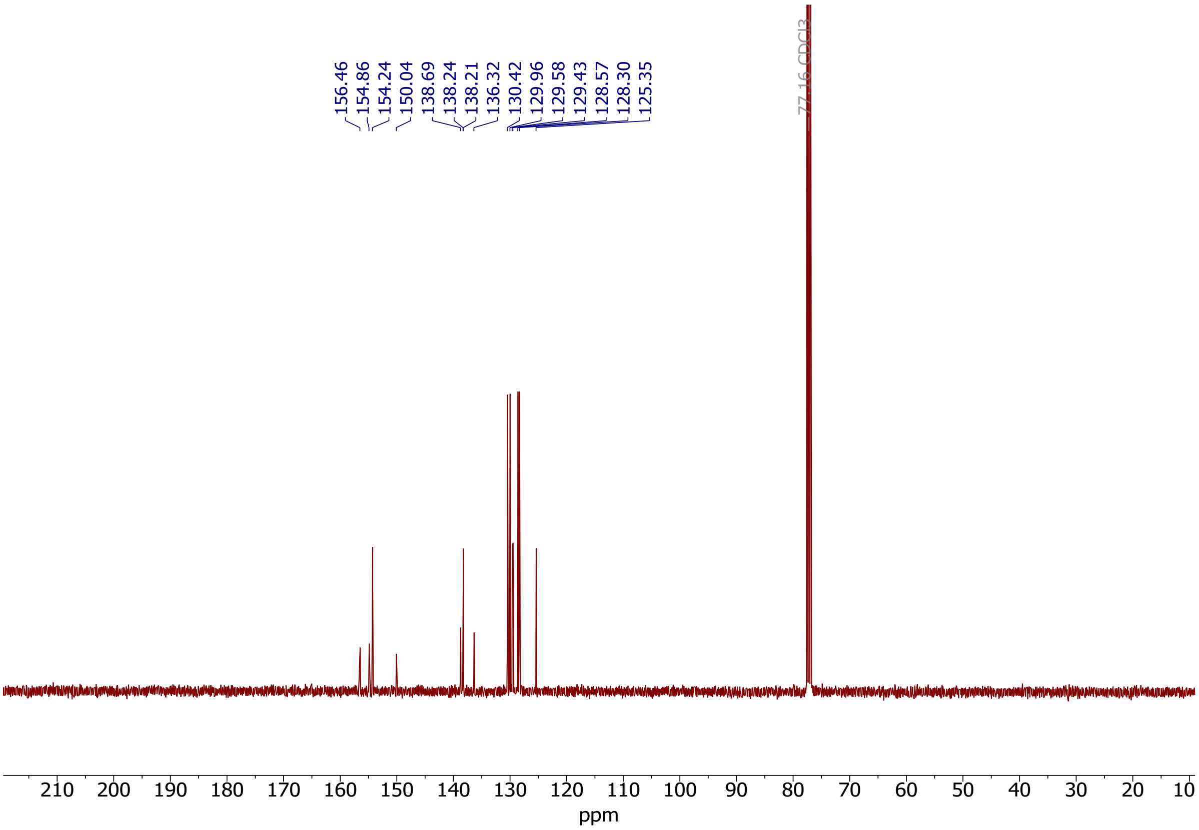

^{1}H NMR (400 MHz, $CDCl_3$) of **PC3** (*see procedure*)

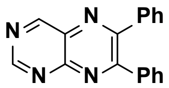

PC3

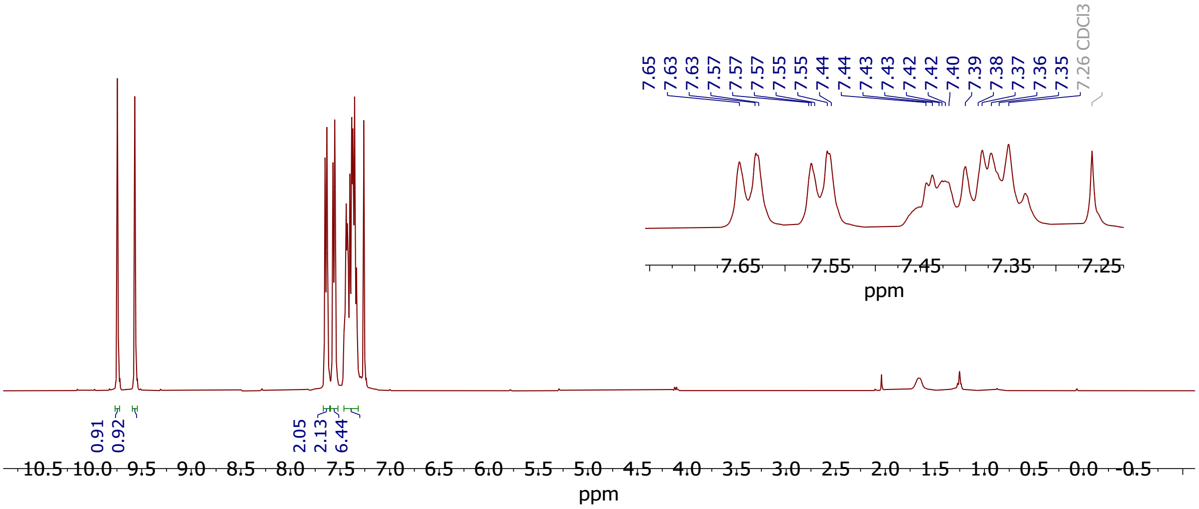

^{13}C NMR (101 MHz, $CDCl_3$) of **PC3**

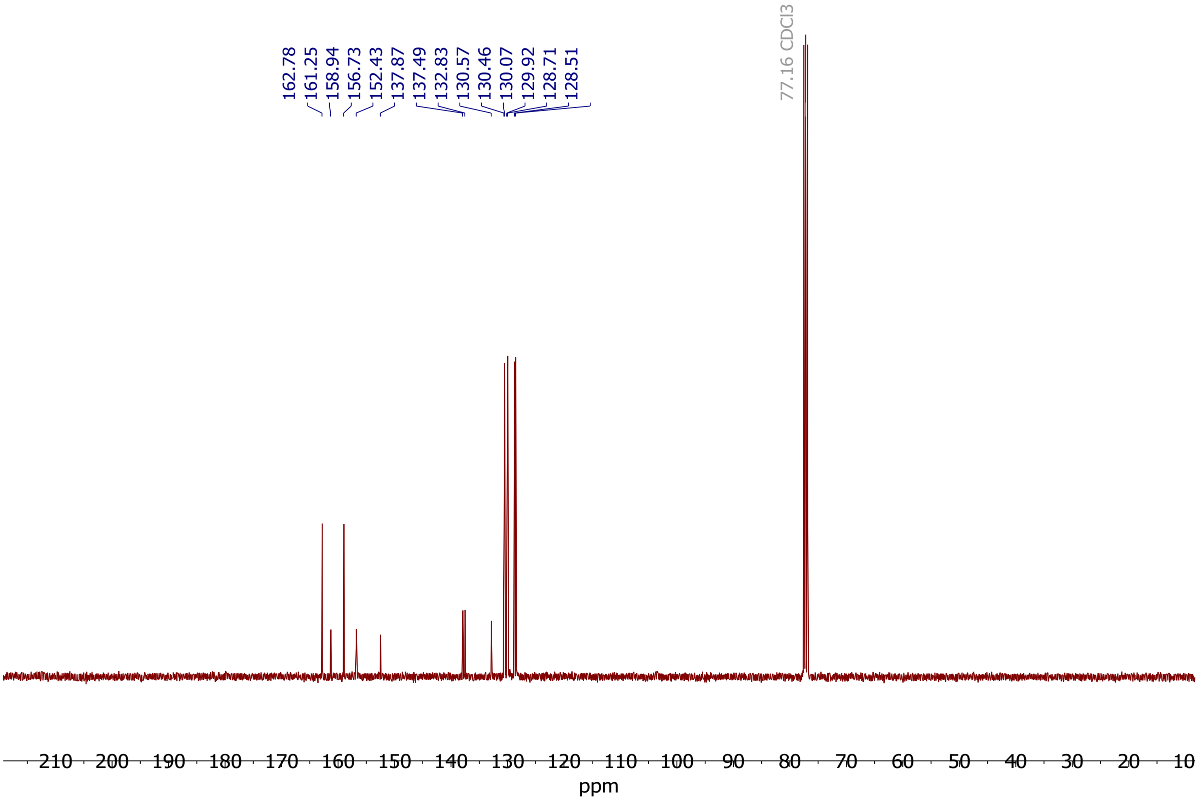

[1]**H NMR** (400 MHz, $CDCl_3$) of **PC4** (*see procedure*)

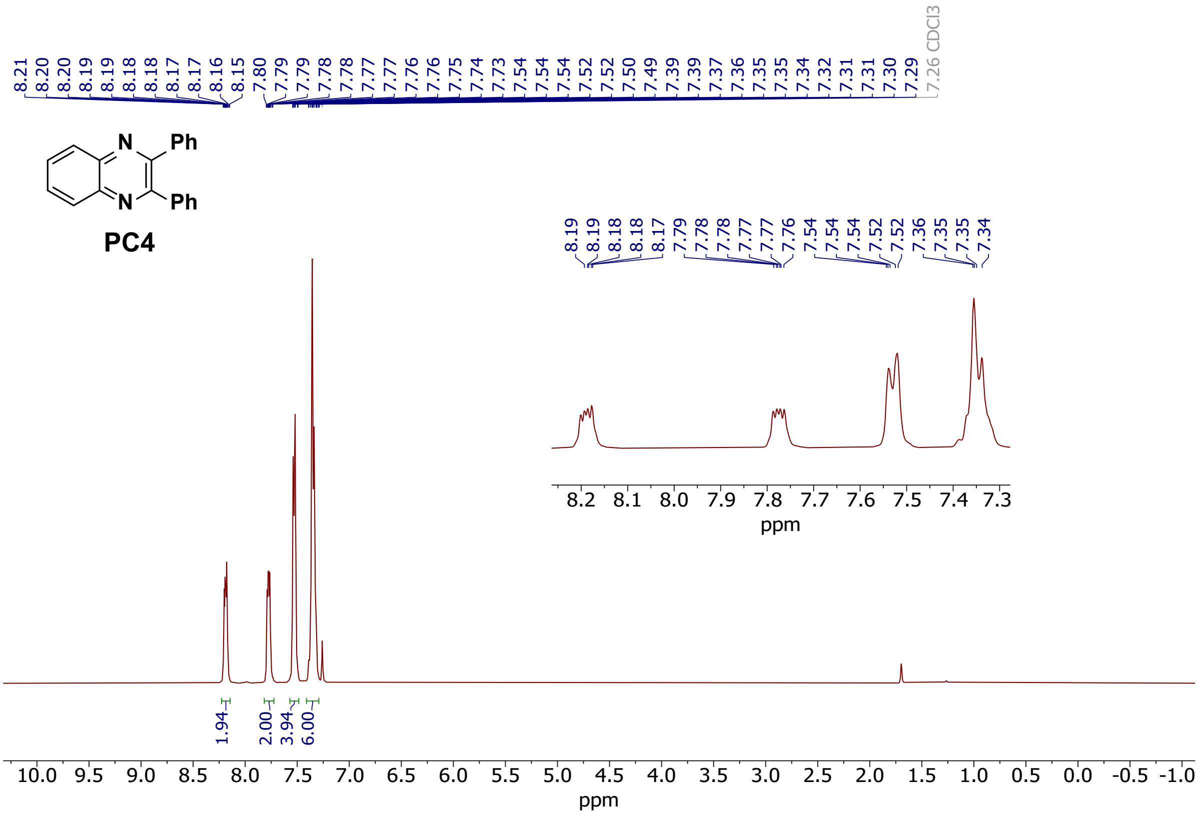

[13]**C NMR** (101 MHz, $CDCl_3$) of **PC4**

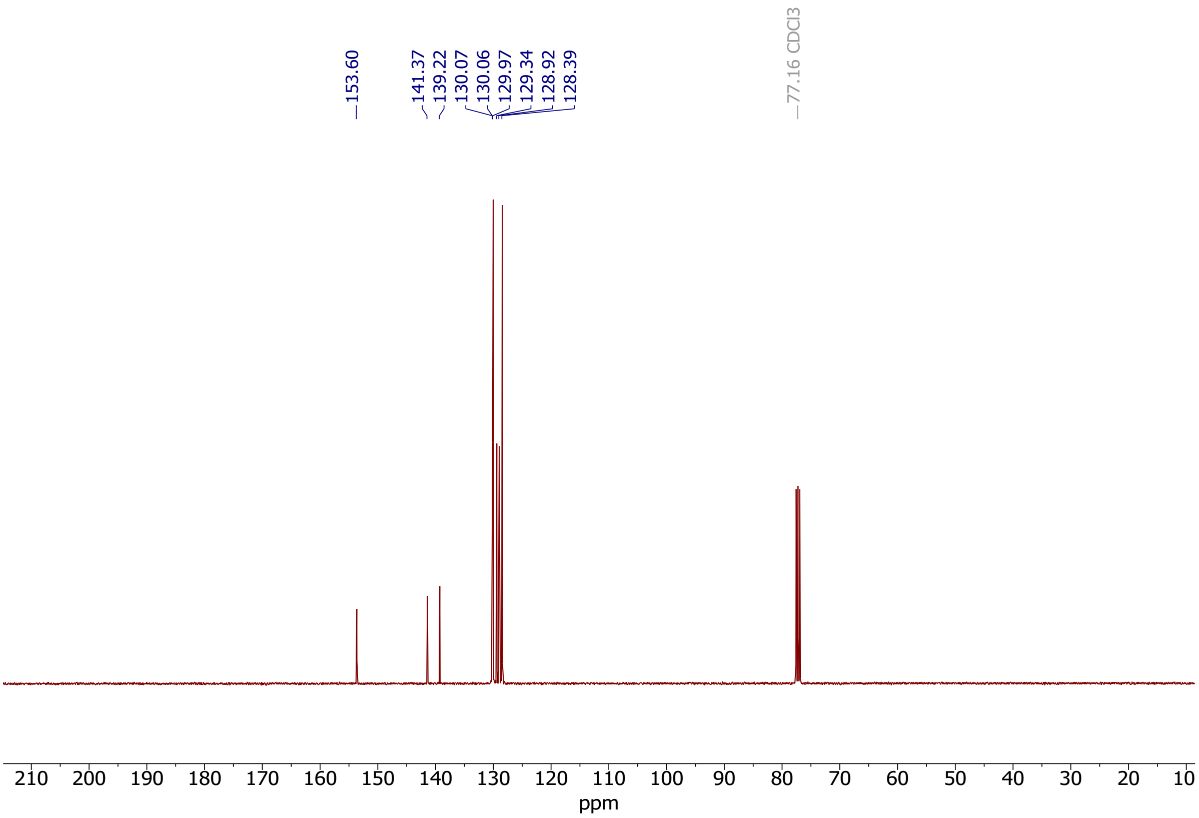

^{1}H NMR (400 MHz, $CDCl_3$) of **4a** (*see procedure*)

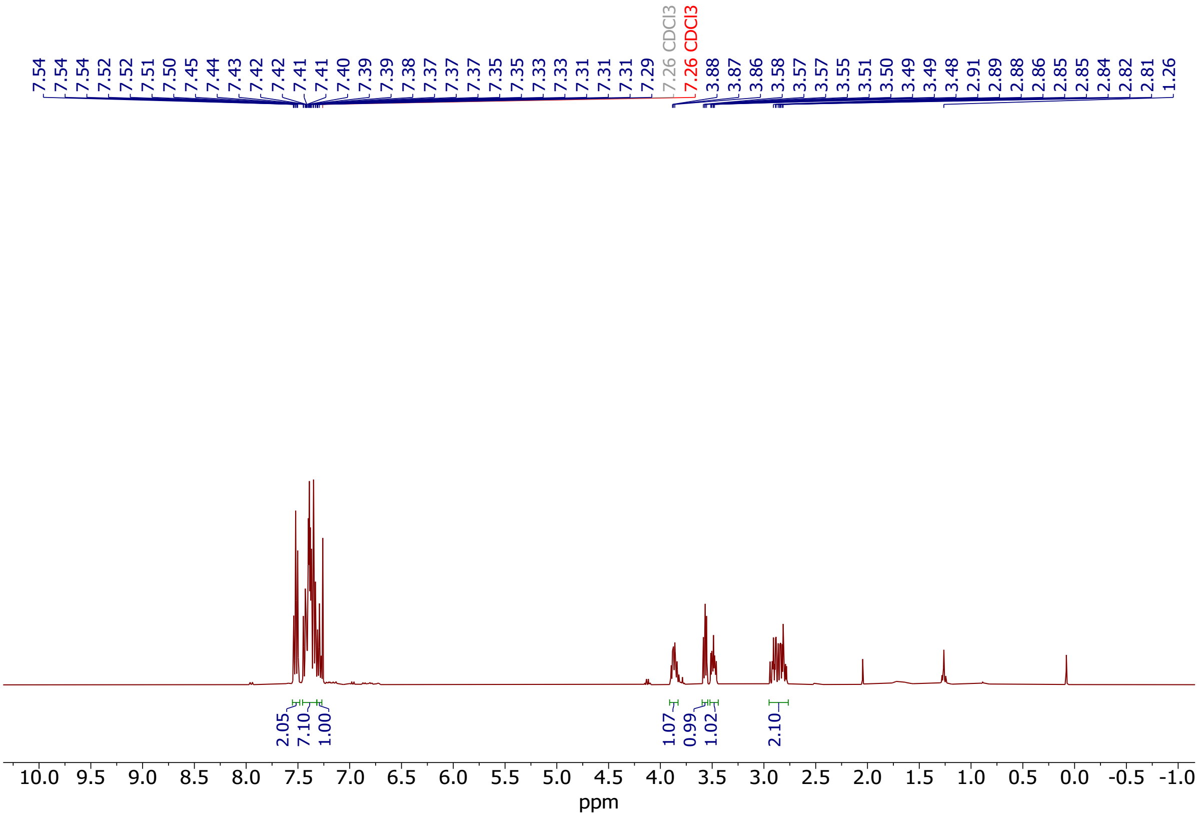

^{13}C NMR (101 MHz, $CDCl_3$) of **4a**

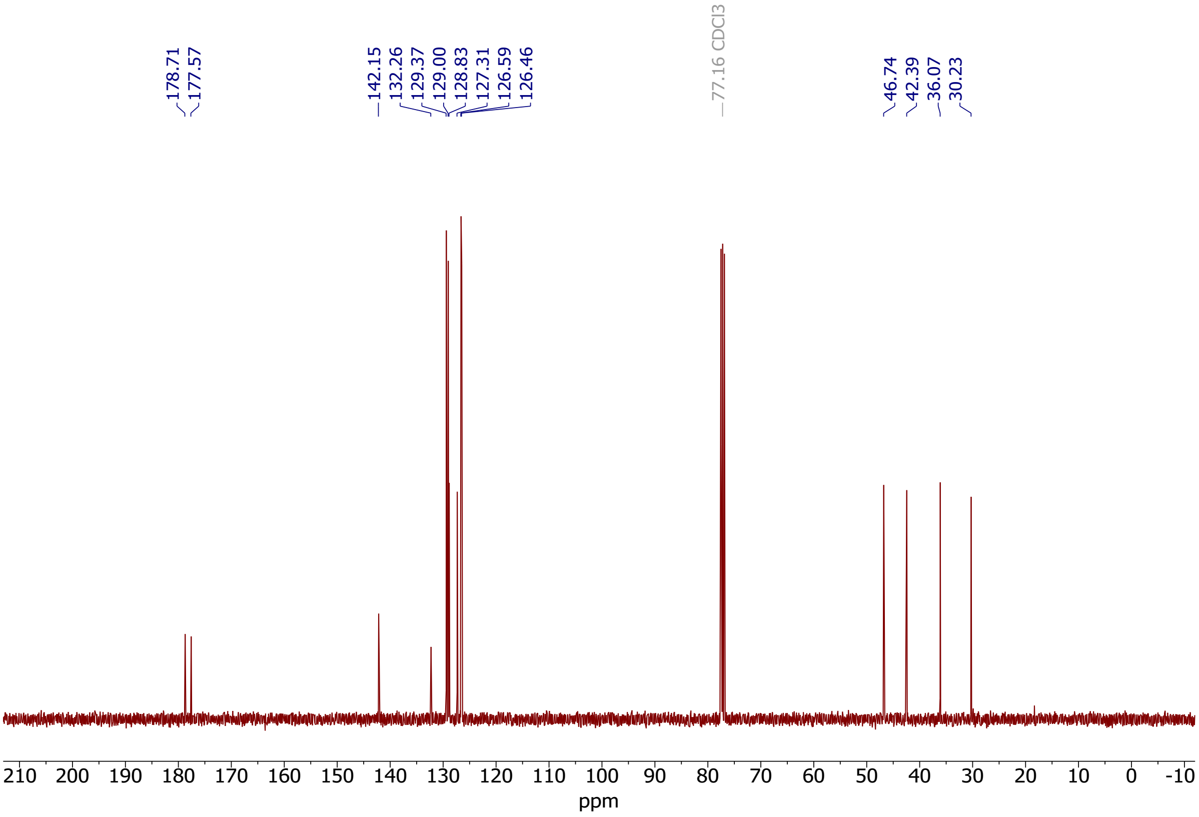

^{1}H NMR (400 MHz, $CDCl_3$) of **4b** (*see procedure*)

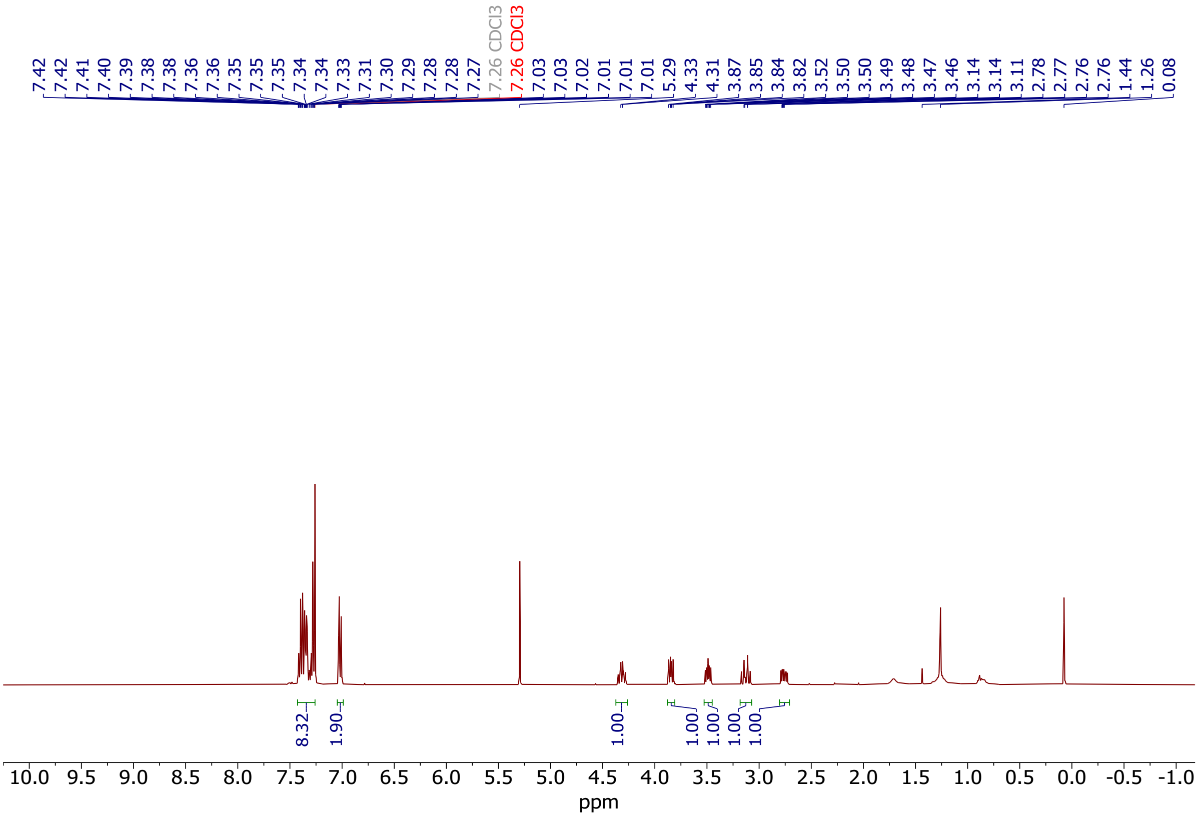

^{13}C NMR (101 MHz, $CDCl_3$) of **4b**

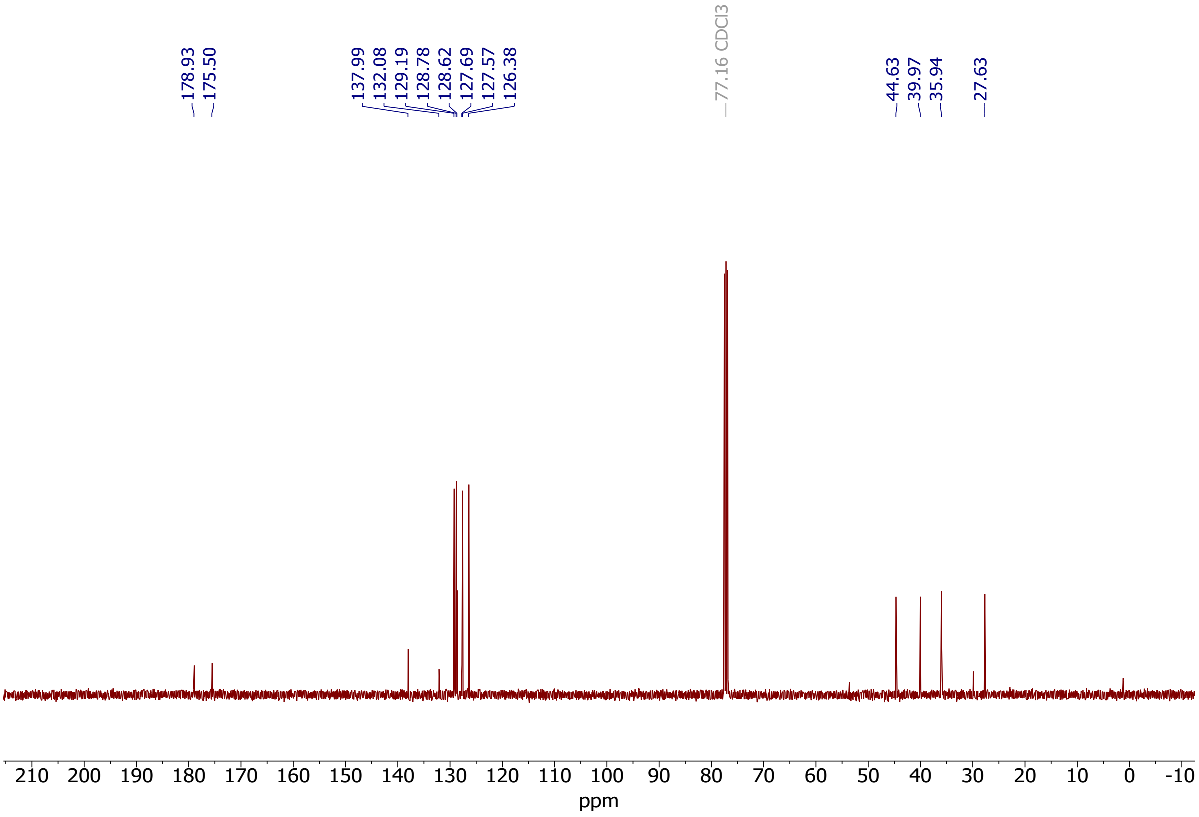

^{1}H NMR (400 MHz, CD_2Cl_2) of **7a** (see procedure)

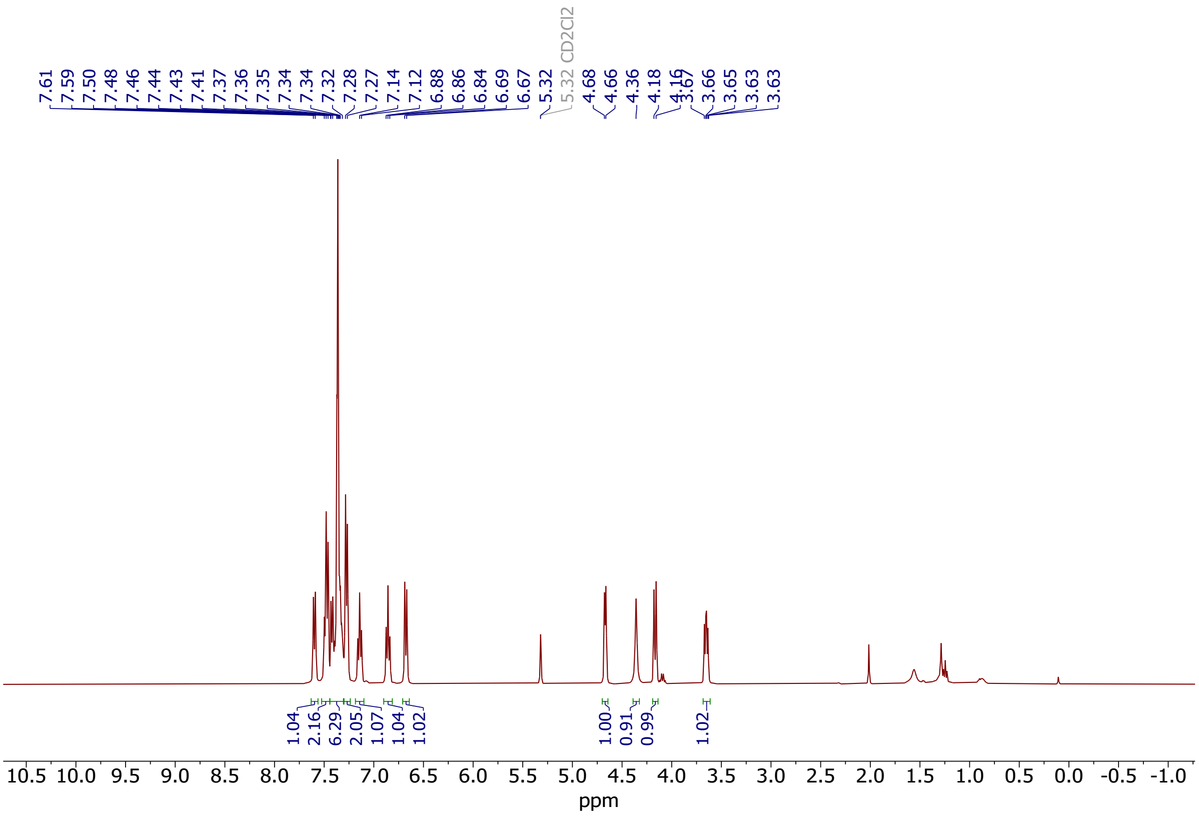

^{13}C NMR (101 MHz, CD_2Cl_2) of **7a**

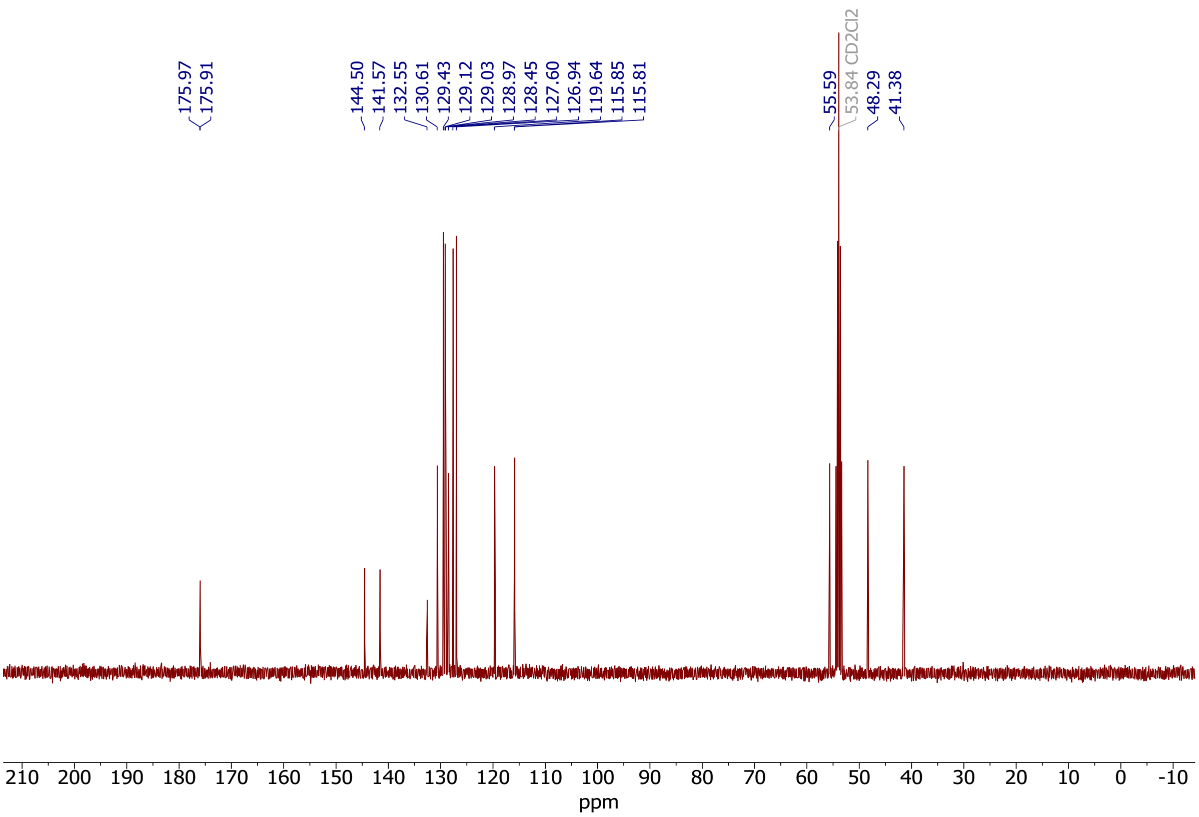

7. REFERENCES

(1) Loeffler, H. H.; He, J.; Tibo, A.; Janet, J. P.; Voronov, A.; Mervin, L. H.; Engkvist, O. Reinvent 4: Modern AI-driven generative molecule design. *J. Cheminform.* **2024**, *16*, 20.

(2) Tingle, B. I.; Tang, K. G.; Castanon, M.; Gutierrez, J. J.; Khurelbaatar, M.; Dandarchuluun, C.; Moroz, Y. S.; Irwin, J. J. ZINC-22—A Free Multi-Billion-Scale Database of Tangible Compounds for Ligand Discovery. *J. Chem. Inf. Model.* **2023**, *63*, 1166–1176.

(3) Huang, D.; Cole, J. M. A database of thermally activated delayed fluorescent molecules auto-generated from scientific literature with ChemDataExtractor. *Sci. Data* **2024**, *11*, 80.

(4) Li, X.; Che, Y.; Chen, L.; Liu, T.; Wang, K.; Liu, L.; Yang, H.; Pyzer-Knapp, E. O.; Cooper, A. I. Sequential closed-loop Bayesian optimization as a guide for organic molecular metallophotocatalyst formulation discovery. *Nat. Chem.* **2024**, *16*, 1286–1294.

(5) Schlosser, L.; Rana, D.; Pflüger, P.; Katzenburg, F.; Glorius, F. EnTdecker - A Machine Learning-Based Platform for Guiding Substrate Discovery in Energy Transfer Catalysis. *J. Am. Chem. Soc.* **2024**, *146*, 13266–13275.

(6) Montalti, M.; Credi, A.; Prodi, L.; Gandolfi, M. T. *Handbook of Photochemistry*; CRC Press, 2006.

(7) Lu, J.; Pattengale, B.; Liu, Q.; Yang, S.; Shi, W.; Li, S.; Huang, J.; Zhang, J. Donor–Acceptor Fluorophores for Energy-Transfer-Mediated Photocatalysis. *J. Am. Chem. Soc.* **2018**, *140*, 13719–13725.

(8) Hojo, R.; Bergmann, K.; Elgadi, S. A.; Mayder, D. M.; Emmanuel, M. A.; Oderinde, M. S.; Hudson, Z. M. Imidazophenothiazine-Based Thermally Activated Delayed Fluorescence Materials with Ultra-Long-Lived Excited States for Energy Transfer Photocatalysis. *J. Am. Chem. Soc.* **2023**, *145*, 18366–18381.

(9) Elliott, L. D.; Kayal, S.; George, M. W.; Booker-Milburn, K. Rational Design of Triplet Sensitizers for the Transfer of Excited State Photochemistry from UV to Visible. *J. Am. Chem. Soc.* **2020**, *142*, 14947–14956.

(10) https://github.com/le-schlo/InvEnT.

(11) Greenman, K. P.; Green, W. H.; Gomez-Bombarelli, R. *UVVisML*; Zenodo, 2021.

(12) Bannwarth, C.; Ehlert, S.; Grimme, S. GFN2-xTB-An Accurate and Broadly Parametrized Self-Consistent Tight-Binding Quantum Chemical Method with Multipole Electrostatics and Density-Dependent Dispersion Contributions. *J. Chem. Theory Comput.* **2019**, *15*, 1652–1671.

(13) Bannwarth, C.; Grimme, S. A simplified time-dependent density functional theory approach for electronic ultraviolet and circular dichroism spectra of very large molecules. *Comput. Theor. Chem.* **2014**, *1040-1041*, 45–53.

(14) Halgren, T. A. Merck molecular force field. I. Basis, form, scope, parameterization, and performance of MMFF94. *J. Comput. Chem.* **1996**, *17*, 490–519.

(15) Spicher, S.; Grimme, S. Robust Atomistic Modeling of Materials, Organometallic, and Biochemical Systems. *Angew. Chem. Int. Ed.* **2020**, *59*, 15665–15673.

(16) Greenman, K. P.; Green, W. H.; Gómez-Bombarelli, R. Multi-fidelity prediction of molecular optical peaks with deep learning. *Chem. Sci.* **2022**, *13*, 1152–1162.

(17) Micikas, R. J.; Ahmed, I. A.; Acharyya, A.; Smith, A. B.; Gai, F. Tuning the electronic transition energy of indole via substitution: application to identify tryptophan-based chromophores that absorb and emit visible light. *PCCP* **2021**, *23*, 6433–6437.

(18) Lu, T. A comprehensive electron wavefunction analysis toolbox for chemists, Multiwfn. *J. Chem. Phys.*

2024, *161*.
(19) Dansholm, C. N.; Junker, A. K. R.; Nielsen, L. G.; Kofod, N.; Pal, R.; Sørensen, T. J. π-Expanded Thioxanthones - Engineering the Triplet Level of Thioxanthone Sensitizers for Lanthanide-Based Luminescent Probes with Visible Excitation. *ChemPlusChem* **2019**, *84*, 1778–1788.
(20) Penfold, T. J. On Predicting the Excited-State Properties of Thermally Activated Delayed Fluorescence Emitters. *J. Phys. Chem. C* **2015**, *119*, 13535–13544.
(21) Kuila, S.; Miranda-Salinas, H.; Eng, J.; Li, C.; Bryce, M. R.; Penfold, T. J.; Monkman, A. P. Rigid and planar π-conjugated molecules leading to long-lived intramolecular charge-transfer states exhibiting thermally activated delayed fluorescence. *Nat. Commun.* **2024**, *15*, 9611.
(22) Xu, S.; Yuan, Y.; Cai, X.; Zhang, C.-J.; Hu, F.; Liang, J.; Zhang, G.; Zhang, D.; Liu, B. Tuning the singlet-triplet energy gap: a unique approach to efficient photosensitizers with aggregation-induced emission (AIE) characteristics. *Chem. Sci.* **2015**, *6*, 5824–5830.
(23) Samanta, P. K.; Kim, D.; Coropceanu, V.; Brédas, J.-L. Up-Conversion Intersystem Crossing Rates in Organic Emitters for Thermally Activated Delayed Fluorescence: Impact of the Nature of Singlet vs Triplet Excited States. *J. Am. Chem. Soc.* **2017**, *139*, 4042–4051.
(24) Lee, K.; Kim, D. Local-Excitation versus Charge-Transfer Characters in the Triplet State: Theoretical Insight into the Singlet–Triplet Energy Differences of Carbazolyl-Phthalonitrile-Based Thermally Activated Delayed Fluorescence Materials. *J. Phys. Chem. C* **2016**, *120*, 28330–28336.
(25) Huang, S.; Zhang, Q.; Shiota, Y.; Nakagawa, T.; Kuwabara, K.; Yoshizawa, K.; Adachi, C. Computational Prediction for Singlet- and Triplet-Transition Energies of Charge-Transfer Compounds. *J. Chem. Theory Comput.* **2013**, *9*, 3872–3877.
(26) Greg Landrum; Paolo Tosco; Brian Kelley; Ric; David Cosgrove; sriniker; Riccardo Vianello; gedeck; NadineSchneider; Gareth Jones; Eisuke Kawashima; Dan N; Andrew Dalke; Brian Cole; Matt Swain; Samo Turk; Aleksandr Savelev; Alain Vaucher; Maciej Wójcikowski; Ichiru Take; Vincent F. Scalfani; Daniel Probst; Kazuya Ujihara; guillaume godin; Axel Pahl; Rachel Walker; Juuso Lehtivarjo; Francois Berenger; strets123; jasondbiggs. *rdkit/rdkit: Release_2023.09.5*; Zenodo, 2024.
(27) Wicker, J. G. P.; Cooper, R. I. Beyond Rotatable Bond Counts: Capturing 3D Conformational Flexibility in a Single Descriptor. *J. Chem. Inf. Model.* **2016**, *56*, 2347–2352.
(28) Ertl, P.; Schuffenhauer, A. Estimation of synthetic accessibility score of drug-like molecules based on molecular complexity and fragment contributions. *J. Cheminform.* **2009**, *1*, 8.
(29) Pettersen, E. F.; Goddard, T. D.; Huang, C. C.; Meng, E. C.; Couch, G. S.; Croll, T. I.; Morris, J. H.; Ferrin, T. E. UCSF ChimeraX: Structure visualization for researchers, educators, and developers. *Protein Sci.* **2021**, *30*, 70–82.
(30) Wang, S.; Witek, J.; Landrum, G. A.; Riniker, S. Improving Conformer Generation for Small Rings and Macrocycles Based on Distance Geometry and Experimental Torsional-Angle Preferences. *J. Chem. Inf. Model.* **2020**, *60*, 2044–2058.
(31) Neese, F. Software Update: The ORCA Program System—Version 6.0. *WIREs Comput Mol Sci* **2025**, *15*.
(32) Neese, F. The SHARK integral generation and digestion system. *J. Comput. Chem.* **2023**, *44*, 381–396.
(33) Garcia-Ratés, M.; Neese, F. Efficient implementation of the analytical second derivatives of hartree-fock

and hybrid DFT energies within the framework of the conductor-like polarizable continuum model. *J. Comput. Chem.* **2019**, *40*, 1816–1828.

(34) Cossi, M.; Rega, N.; Scalmani, G.; Barone, V. Energies, structures, and electronic properties of molecules in solution with the C-PCM solvation model. *J. Comput. Chem.* **2003**, *24*, 669–681.

(35) Chai, J.-D.; Head-Gordon, M. Long-range corrected hybrid density functionals with damped atom-atom dispersion corrections. *PCCP* **2008**, *10*, 6615–6620.

(36) Schäfer, A.; Horn, H.; Ahlrichs, R. Fully optimized contracted Gaussian basis sets for atoms Li to Kr. *J. Chem. Phys.* **1992**, *97*, 2571–2577.

(37) Neese, F.; Wennmohs, F.; Hansen, A.; Becker, U. Efficient, approximate and parallel Hartree–Fock and hybrid DFT calculations. A ‘chain-of-spheres’ algorithm for the Hartree–Fock exchange. *Chem. Phys.* **2009**, *356*, 98–109.

(38) Izsák, R.; Neese, F. An overlap fitted chain of spheres exchange method. *J. Chem. Phys.* **2011**, *135*, 144105.

(39) Schäfer, A.; Huber, C.; Ahlrichs, R. Fully optimized contracted Gaussian basis sets of triple zeta valence quality for atoms Li to Kr. *J. Chem. Phys.* **1994**, *100*, 5829–5835.

(40) https://github.com/radi0sus/orca_uv.

(41) Souza, B. de; Neese, F.; Izsák, R. On the theoretical prediction of fluorescence rates from first principles using the path integral approach. *J. Chem. Phys.* **2018**, *148*, 34104.

(42) Souza, B. de; Farias, G.; Neese, F.; Izsák, R. Predicting Phosphorescence Rates of Light Organic Molecules Using Time-Dependent Density Functional Theory and the Path Integral Approach to Dynamics. *J. Chem. Theory Comput.* **2019**, *15*, 1896–1904.

(43) Paul, L.; Moitra, T.; Ruud, K.; Chakrabarti, S. Strong Duschinsky Mixing Induced Breakdown of Kasha's Rule in an Organic Phosphor. *J. Phys. Chem. Lett.* **2019**, *10*, 369–374.

(44) Aghapoor, K.; Darabi, H. R.; Mohsenzadeh, F.; Balavar, Y.; Daneshyar, H. Zirconium(IV) chloride as versatile catalyst for the expeditious synthesis of quinoxalines and pyrido[2,3-b]pyrazines under ambient conditions. *Transit. Met. Chem.* **2010**, *35*, 49–53.

(45) Darabi, H. R.; Mohandessi, S.; Aghapoor, K.; Mohsenzadeh, F. A recyclable and highly effective sulfamic acid/MeOH catalytic system for the synthesis of quinoxalines at room temperature. *Catal. Commun.* **2007**, *8*, 389–392.

(46) Yadav, J.; Reddy, B.; Premalatha, K.; Shiva Shankar, K. Bismuth(III)-Catalyzed Rapid Synthesis of 2,3-Disubstituted Quinoxalines in Water. *Synthesis* **2008**, *2008*, 3787–3792.

(47) Skolia, E.; Kokotos, C. G. Photochemical 2 + 2 Cycloaddition of Alkenes with Maleimides: Highlighting the Differences between N-Alkyl vs N-Aryl Maleimides. *ACS Org. Inorg. Au* **2023**, *3*, 96–103.
